# Halogenated *N*-phenylpiperazine and 2-(piperazin-1-yl)pyrimidine as novel cucurbit[7]uril guests: experimental and computational insights into supramolecular binding

**DOI:** 10.1039/d5ra07259j

**Published:** 2025-11-20

**Authors:** David A. Rincón, Ewelina Zaorska, Maura Malinska

**Affiliations:** a Faculty of Chemistry, University of Warsaw Pasteura 1 02-093 Warsaw Poland mmalinska@chem.uw.edu.pl

## Abstract

This study presents a new class of halogenated *N*-phenylpiperazine and 2-(piperazin-1-yl)pyrimidine derivatives as guests for cucurbit[7]uril (CB[7]), expanding the space of CB[7]-binding ligands. Combining isothermal titration calorimetry (ITC), X-ray crystallography, and computation (attach–pull–release, APR; symmetry-adapted perturbation theory, SAPT), we quantify how halogen identity and position modulate host–guest binding. We find that halogenation provides two position-specific levers for tuning affinity. At the *ortho* position, both F and Cl enhance dispersion (Cl more strongly), while *ortho*-F additionally confers pre-organization (intramolecular C–H⋯F) that reduces the entropic penalty. Across the series, the lowest free energies of binding (Δ*G*) are observed for ligands with *ortho*-F, consistent with entropy reduction *via* pre-organization. By contrast, para-substituent effects become significant mainly for larger halogens (Br, I), which can engage the carbonyl-lined portal and enhance enthalpic stabilization. These findings provide a rational strategy for optimizing ligand properties *via* supramolecular recognition, offering new perspectives for host–guest chemistry.

## Abstract 2

This study presents a new class of halogenated *N*-phenylpiperazine and 2-(piperazin-1-yl)pyrimidine derivatives as guests for cucurbit[7]uril (CB[7]), expanding the space of CB[7]-binding ligands.

## Graphical abstract

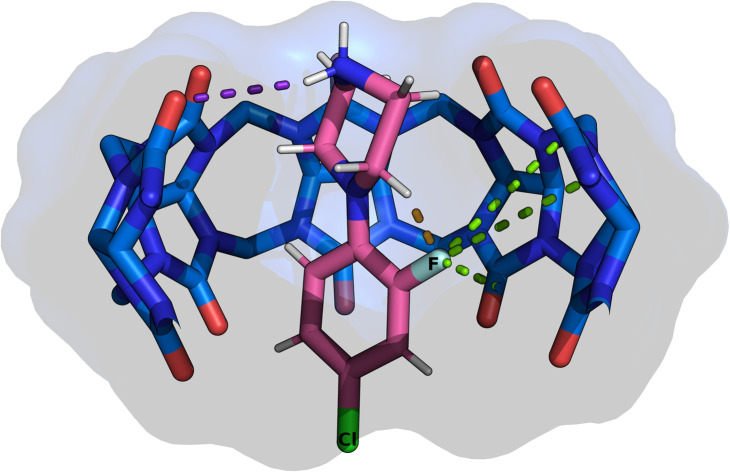

## Introduction

Cucurbit[7]uril (CB[7]) is a versatile supramolecular host with applications spanning various fields such as fluorescence enhancer of dyes,^1^ controlled drug delivery through molecular encapsulation,^2–4^ and analytical chemistry.^5–11^ Additionally, CB[7] has found use in supramolecular catalysis,^12–21^ supramolecular polymers,^22^ as a protein crystallization aid,^23,24^ and luminescent emissions arising from macrocyclic confinement.^25^ Despite these wide-ranging applications, relatively few studies have combined both experimental and computational approaches to investigate CB[7]'s interactions with different ligands,^16,26^ amino-acid-specific sequences and proteins.^23,27–29^

CB[7] is a non-toxic macrocycle with promising applications in drug delivery and sensing in biological samples (*e.g.*, blood and urine).^30^ A critical aspect of its effectiveness is selectivity, which depends on binding affinity differences. For effective analyte recovery and potential reuse of the sensor or sorbent, strong ligand binding is essential. CB[7] can form ultrahigh-affinity host–guest complexes with ferrocene and adamantane-based amines, reaching binding constant (*K*_a_) values in the range of 10^12^ to 10^15^—comparable to or even exceeding the biotin–streptavidin interaction (*K*_a_ ∼10^13^ M^−1^) values. Despite these impressive affinities, the origin of such stability remains not fully understood.^31^

Nevertheless, CB[7] has already been tested for various applications, and differences in ligand binding affinities are often attributed to ion–dipole interactions,^32^ London dispersion interactions,^33,34^ strong hydrophobic effects—such as high-energy cavity water release^35,36^ or cavitation energy.^34^ Binding entropy may also contribute significantly, though it remains challenging to assess both experimentally and theoretically.^33–36^ CB[7] thus serves as an excellent model system for exploring the complex relationship between thermodynamic profiles and complex formation.^31,36–39^ With its portals characterized by a negative electrostatic potential, hydrophobic cavity, and water solubility, CB[7] provides insights into the intricate balance of non-covalent interactions, ligand acidity, long-range interactions, conformational flexibility, and water dynamics in molecular binding. Applying well-established medicinal chemistry principles is essential for practical applications and a deeper understanding of CB[7]-ligand interactions.

An increasing number of studies utilizing isothermal titration calorimetry (ITC) have provided valuable thermodynamic profiles of CB[7] complexes. When crystal structures are available, correlations between thermodynamics and host–guest geometry can be explored. A rough link between buried apolar surface area and free energy has been noted in proteins.^40^ However, translating this concept into practical insights for supramolecular systems remains challenging. The combination of 3D structure analysis with binding free energy calculations does not provide a complete understanding of the energetic contributions of individual interactions. While it would be desirable to correlate observed interactions with specific energy terms, molecular interactions exhibit a highly non-additive nature.^33,34^

Nonetheless, there is a plethora of non-covalent interactions that can be utilized to optimize binding affinity, including hydrogen bonds, halogen bonds, π–π stacking, and dispersive interactions.^41–44^ The individual contributions of these interactions to binding affinity vary significantly and depend heavily on the specific system under investigation. Among these interactions, halogen bonds^45^ are particularly noteworthy as the second most important non-covalent interactions after hydrogen bonds, due to their versatility in biological and supramolecular systems. Halogen substitutions are widely implemented to improve solubility, bioavailability, and biological activity.^46,47^ A halogen bond forms between the σ-hole—an electron-deficient region on a halogen atom—and a Lewis base, such as the oxygen atom of a carbonyl group. The strength of the halogen bond depends on the size and electron-deficient character of the σ-hole, which varies across halogens. Iodine typically forms the strongest bonds, followed by bromine and chlorine, whereas fluorine usually does not participate in halogen bonding due to its small size and high electronegativity. Nonetheless, fluorine substitution is frequently employed in drug design to optimize properties such as lipophilicity, metabolic stability, and binding affinity.^48^ Fluorine can also engage in weak yet significant interactions with carbonyl groups, lowering free energy of binding and pharmacokinetics.^49–51^

**Fig. 1:**
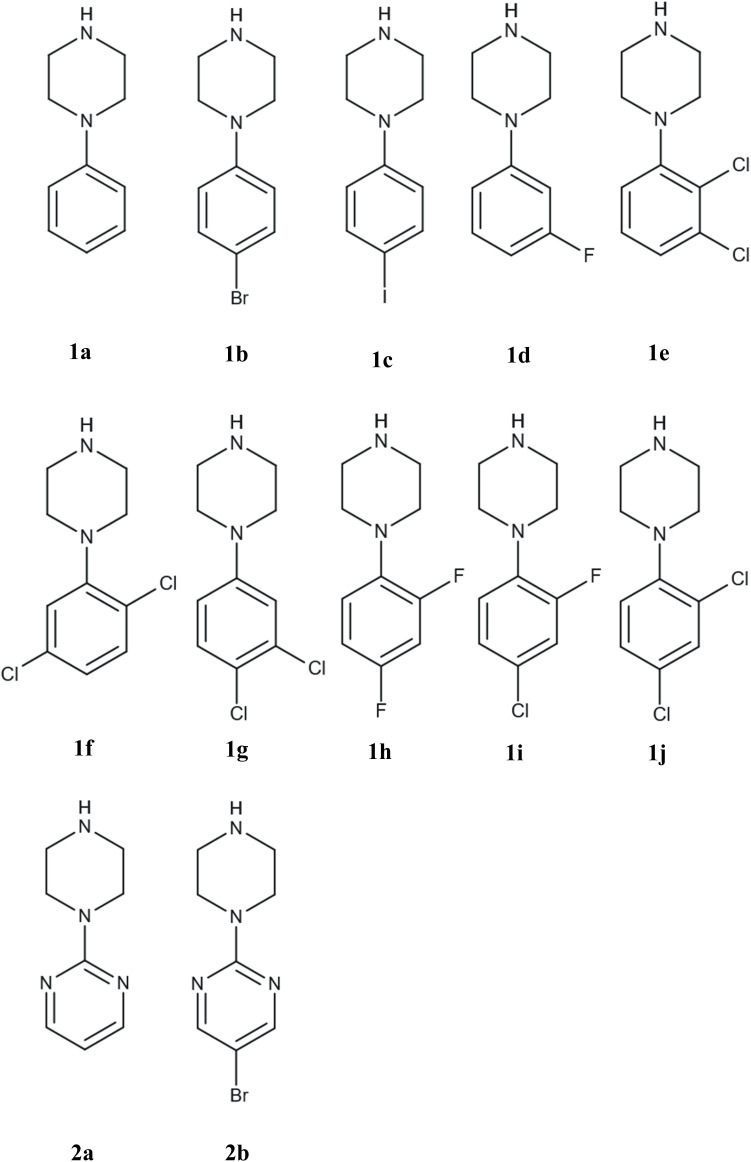
Molecular structure of selected 1-phenylpiperazine. Their names are: 1-phenylpiperazine (1a), 1-(4-bromophenyl)piperazine (1b), 1-(4-iodophenyl)piperazine (1c), 1-(3-fluorophenyl)piperazine (1d), 1-(2,3-dichlorophenyl)piperazine (1e), 1-(2,5-dichlorophenyl)piperazine (1f), 1-(3,4-dichlorophenyl)piperazine (1g), 1-(2,4-difluorophenyl)piperazine (1h), 1-(4-chloro-2-fluorophenyl)piperazine (1i), 1-(2,4-dichlorophenyl)piperazine (1j), 1-(2-pyrimidyl)piperazine (2a), and 5-bromo-2-(piperazin-1-yl)pyrimidine (2b).

This study focuses on the interactions between CB[7] and 1-phenylpiperazine (PhP) and 2-(piperazin-1-yl)pyrimidine (PyP) analogues (Fig. 1), which are widely used in pharmacological assays.^52–59^ Some of these compounds are metabolites of drugs and exhibit biological activity at adrenergic, dopaminergic, and serotonergic receptors.^54,60–63^ Particular attention is given to analogues with halogen substitutions (X = F, Cl, Br, and I). In contrast to benchmark CB[7] complexes with ultrahigh affinities (*e.g.*, adamantane or ferrocene derivatives), the present work deliberately focuses on drug-like scaffolds that form complexes of only moderate (micromolar) affinity. The novelty of this study lies in uncovering how halogen substitution influences binding thermodynamics, conformational entropy, and host–guest geometry, which are relevant for understanding CB[7] recognition of pharmacologically relevant fragments. To this end, we combined isothermal titration calorimetry (ITC), crystallization, and X-ray diffraction (XRD) with computational approaches: binding free energies from the Attach–Pull–Release^64^ (APR) method and interaction energy decomposition using Symmetry-Adapted Perturbation Theory^65^ (SAPT), and the Quantum Theory of Atoms in Molecules^66^ (QTAIM) analysis.

## Methodology

### Experimental methods

#### Materials

CB[7] was purchased from BLD Pharm. All PhP and PyP derivatives (Fig. 1) were purchased from Merck/Sigma Aldrich. All solvents used for crystallization were purchased from Chempur (Poland). All chemicals were of reagent grade and used without further purification.

#### ITC measurements

All the ITC experiments were performed using a MicroCal PEAQ-ITC (Malvern Panalytical Ltd) at pH 4.0. The acidic conditions (pH 4.0) were chosen to ensure full protonation of the piperazine nitrogen atoms and to maintain solubility and crystallization stability of the CB[7] complexes. While this pH is below physiological conditions, it provides reproducible conditions for dissecting the structural and thermodynamic role of halogen substitution. The biomedical relevance should therefore be viewed as conceptual rather than direct, and further studies at physiological pH would be needed.

The stock solutions of CB[7] and PhP or PyP derivatives were individually prepared using acetic acid–sodium acetate buffer (20 mM, pH 4.0) or phosphate–citrate buffer (20 mM, pH 4.0) and diluted as required. The 20 mM acetic acid–sodium acetate buffer (pH 4.0) was made by dissolving 10 mg sodium acetate trihydrate (CH_3_COONa·3H_2_O) and 30 mg acetic acid (CH_3_COOH) in 20 mL of distilled water. The final desired pH was adjusted with HCl or NaOH. A typical titration involved 20 injections at 2 min intervals consisting of 2 µL aliquots of solutions of phenylpiperazine derivatives (1.25–30.0 mM) into the sample cell containing CB[7] (0.25–4.0 mM). The solution in the titration cell was stirred at 750 rpm throughout the experiments, and its temperature was maintained at 25 °C. To determine the heat of dilution, blank experiments were also performed by titrating substrate into the buffer alone. The resulting data were then analyzed to determine the binding stoichiometry (*n*), affinity (*K*_D_), Gibbs free energy of binding (Δ*G*), enthalpy (Δ*H*) and entropy (Δ*S*) with the computer program MicroCal PEAQ-ITC Analysis Software v1.41 (Fig. S1–S13). The experimental conditions used for the ITC measurements are shown in Table S1.

#### Crystallization

Crystals of the complexes were obtained by crystallization from saturated solutions of the host (CB[7]) with guest molecules: 1a, 1h, 1i, 2a. All crystals were obtained by dissolving CB[7] and the chosen PhP or PyP derivatives in appropriate buffer solution at elevated temperature. The obtained solutions were then slowly cooled and evaporated to form crystals. The crystal growth period ranged from a few days up to about two weeks. The crystallization conditions are summarized in Table S2.

#### X-ray diffraction

X-ray diffraction (XRD) data were collected using colourless, transparent, and well-formed crystals on an Agilent SuperNova diffractometer, which was controlled by the CrysAlis PRO software and equipped with a CuKα micro-focus X-ray source (*λ* = 1.54 Å, 50.0 kV, and 0.8 mA) and a HyPix detector or Atlas detector. The experiments were carried out at 100.0(2) K controlled using an Oxford Cryosystems cooling device. The crystals were placed on a MiTeGen mount with a droplet of immersion oil and immediately cooled. Each crystal was positioned 54.0 mm from the detector. Indexing and integration were performed using the CrysAlis PRO software. The structures were solved using SHELXT^67^ and refined using SHELXL.^68^ Refinement was based on *F*^2^ for all reflections, except those with negative intensities. Weighted *R*-factors (w*R*) and all goodness-of-fit values (*S*) were based on *F*^2^, whereas conventional *R*-factors were based on amplitudes, with *F* set to zero for negative *F*^2^. The atomic scattering factors were obtained from the International Tables for Crystallography.^69^ Data collection and processing statistics are summarized in Table S3. Figures S14–S17 show the asymmetric units (side and top views) with ADPs for non-hydrogen atoms, together with representative difference-Fourier residual maps. Residual features within the CB[7] cavity are consistent with an additional, low-occupancy guest position that could not be modeled at the available data quality and resolution.

### Computational methods

The geometries of the complexes were obtained from XRD structures and were optimized with the r^2^SCAN^70^ density functional and the cc-pVDZ Dunning's basis set.^71–75^ Then, a single-point calculation was carried out at the TPSS0 (ref. 76) density functional level with the cc-pVTZ basis set. All optimizations were performed with ORCA.^77,78^ The solvent effects of water were considered by using the conductor-like polarizable continuum model (C-PCM).^79^

#### Theoretical Gibbs free energy

The equation for estimation of the binding Gibbs free energy^80^ is:

1 Δ*G*_calcd_ = Δ*E*_int_ + ΔD3_2body_ + Δ*G*^COSMO^_solv_ − *T*Δ*S* + *E*_(def,host)_ + *E*_(def,guest)_

The first term is the interaction energy, the second corresponds to the two-body dispersion energy, which corresponds to a term in the SAPT calculation, the third term is the Gibbs free energy of solvation from COSMO-RS which stands for COnductor-like Screening MOdel – for Real Solvents^81^ implemented in the software of Amsterdam Modeling Suite^82^ and its referenced level of theory is BP86 (ref. 83)/TZP,^84^ the fourth term is minus the temperature times the AMBER entropy energy, and the fifth and sixth term correspond to the deformation energies for the host and guest upon ligand binding.

#### Other thermodynamic relations

The systems under examination herein demonstrate the presence of disorder in the position of the ligand within the crystal structure. The total thermodynamic property is calculated as the sum of all obtained thermodynamic properties for each binding conformation (from 1 to *N*), as follows:2
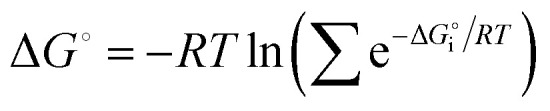
where *R* is the gas constant and *T* is the absolute temperature in K.

The interaction energy is defined as the difference between the energy of the complex and the energy of the isolated monomers in the geometry of the complex.^85^

3 Δ*E*_int_ = *E*_(complex)_ − (*E*_(host with the geometry in the complex)_ + *E*_(guest with the geometry in the complex)_)

The deformation energy was calculated as:

4 Δ*E*_def_ = *E*_(geometry in the complex)_ − *E*_(geometry in the unbound state)_

The geometry of the host and guest in the complex was calculated using a single point. In the unbound state, the geometry of the host and guest was optimized. The aforementioned calculations were conducted utilizing the TPSS0 density functional, the cc-pVTZ Dunning's basis set, and in gas phase. The software employed was ORCA.^77,78^

#### Symmetry-adapted perturbation theory

The host–guest interactions between CB[7], PhP and PyP were studied using SAPT. The closed shell SAPT calculations were carried on using Psi4 program.^86^ The SAPT method can be used to determine the energies of non-covalent intermolecular interactions between molecules by decomposing them into their individual components, such as electrostatic, inductive, dispersion and exchange interactions.^65,86^

5 *E*_SAPT0_ = *E*^(10)^_elst_ + *E*^(10)^_exch_ + *E*^(20)^_ind, resp_ + *E*^(20)^_exch-ind,resp_ + *E*^(20)^_disp_ + *E*^(20)^_exch-disp_ + δ*E*^(2)^_HF_

The first two terms in the equation above represent the electrostatic energy and its exchange counterpart. The following two elements are the induction energy and its exchange equivalent. The subscript resp indicates the response of a monomer's Hartree–Fock orbitals to the electrostatic potential of another monomer. The only terms in this equation that represent the actual electronic correlation effects are the fifth and sixth terms. The dispersion energy, which is the fifth term, provides a reliable approximation of the van der Waals dispersion energies. The dispersion energy's exchange counterpart is represented by the sixth term. The third and higher-order induction energy corrections are taken into account in the final term.^65,86^

The simplest SAPT method corresponds to SAPT0. This method was employed in this paper in conjunction with the cc-pVTZ basis set on the previously optimized complexes.

#### Molecular dynamics simulations

The geometry of the systems was obtained from crystallographic data. Moreover, MD simulations were performed using parallel threaded computations with the Intel compiler suite and the Intel MPI version of the pmemd. MPI program implemented in AMBER16.^87^ The MD simulation box was treated with periodic boundary conditions and contained the host, the guest, 6 sodium and 7 chlorine counterions, and ∼2000 water molecules (TIP3P).^88^ Simulations were performed at constant temperature of 298.15 K, using a Langevin thermostat,^89–91^ and pressure of 1 bar, using a Berendsen barostat.^92^ A cutoff of 12 Å was used for non-bonded interactions, with long-range electrostatic interactions accounted for by the PME method.^93^ A time step of 2 fs was used in MD simulations according to recommendations from the literature.^94^

The absolute Gibbs energies of binding (Δ*G*_bind_) of each ligand were calculated using the APR method. This method allows accurate calculations of supramolecular host–guest binding. Three dummy atoms were used to set up the restraints required by the APR method, along with two atoms of the guest and three of the host atoms. The distance force constant was set to 5.0 kcal (mol Å^2^)^−1^, while the angle and torsional force constants were set to 100.0 kcal (mol rad^2^)^−1^. The distance between a guest atom and a dummy atom placed at the bottom of the cage with the ligand was increased by 0.4 Å during the simulation from 0 Å (ligand inside the cage) to 18 Å (ligand outside the cage). For each simulated window, equilibration and accumulation included a 2 ps step size for equilibration at 298.15 K *NPT* equilibration, and 10 ns *NPT* production per window. Hydrogen mass repartitioning was not allowed.^94^

The applied approach to compute binding enthalpies within the APR scheme is termed single-box and computes binding enthalpies of the bound state and the unbound state. The difference of the potential energies is then obtained between those two states.^95^

#### Quantum theory of atoms in molecules

QTAIM analysis of the electron density *ρ*(*r*_c_) for all optimized systems was conducted using the AIMALL software.^96^ This analysis enables the identification of atomic basins and the associated properties, including the atomic charge, *q*(*Ω*), and atomic energy, *E*(*Ω*). In addition, topological properties at bond critical points (BCP), such as the electron density, *ρ*(*r*_c_), Laplacian of the electron density, *∇*^2^*ρ*(*r*_c_), local potential energy density, *V*(*r*_c_), local kinetic energy density, *G*(*r*_c_), and local energy density, *H*(*r*_c_), were analyzed. This final topological property is derived from the Cremer–Kraka relationship.^97^

Hayashi *et al.*^98^ introduced a procedure to classify bonds based on the Cremer–Kraka relationship:

6 *H*(*r*_c_) = *V*(*r*_c_) + *G*(*r*_c_)

7
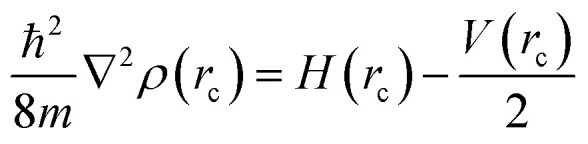


The analysis is based on previous equations, where the first equation relates the local potential energy density, *V*(*r*_c_), with the local kinetic energy density, *G*(*r*_c_), to obtain the local energy density, *H*(*r*_c_). The second equation relates a constant *ℏ*/8*m*, where *ℏ* is the reduced Planck constant and *m* is the electron mass, times the Laplacian *∇*^2^*ρ*(*r*_c_) with the local potential and total energy densities. Based on these relationships, bonds are classified according to their respective values of *H*(*r*_c_) and *∇*^2^*ρ*(*r*_c_).

Furthermore, the energy of the intermolecular interactions was determined using the equation proposed by Afonin *et al.*^99^

8 *E* = −172.5 × *V*_(BCP)_ + 0.33

where *V* is the electron potential energy density in the hydrogen bond critical point.

## Results

### Experimental studies

#### Binding affinity of PhP and PyP derivatives with CB[7]

**Fig. 2:**
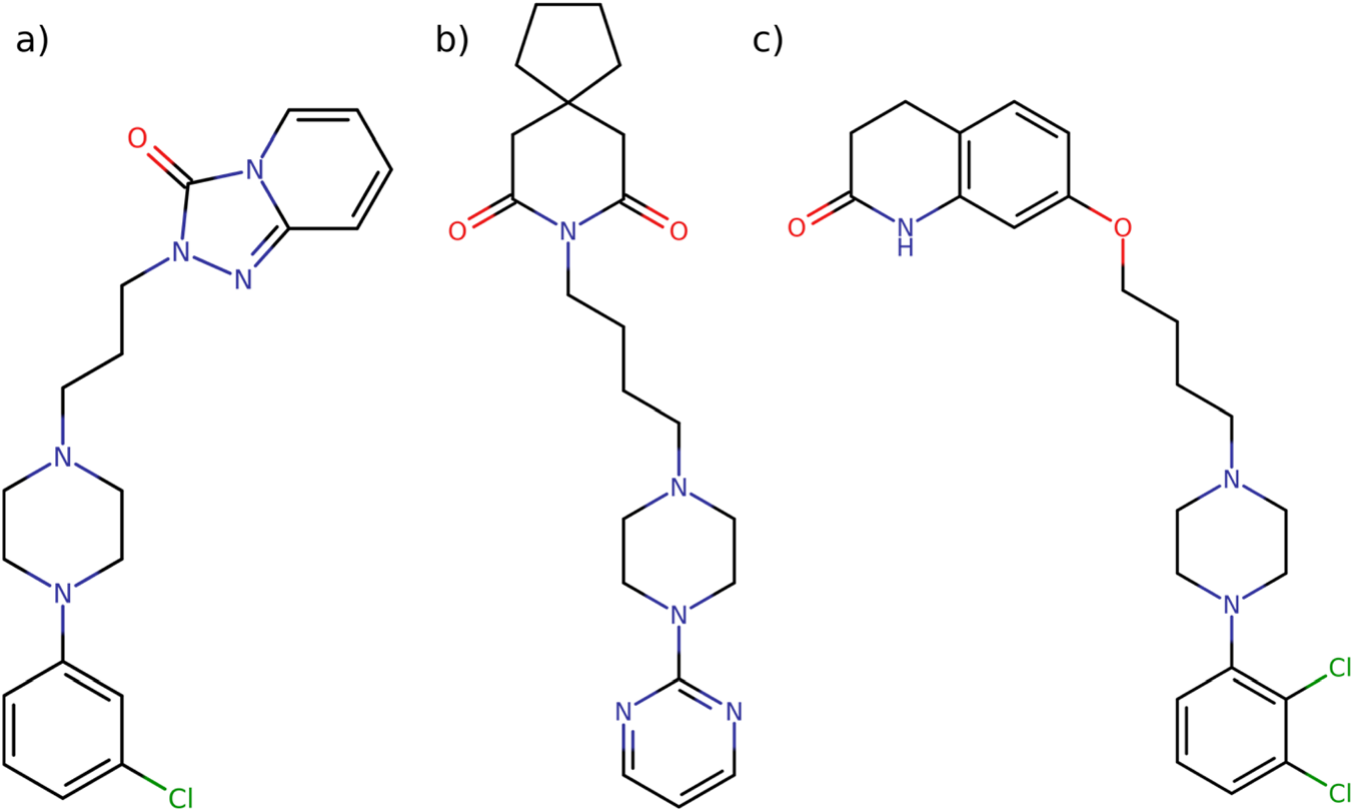
Chemical structures of three drugs containing *N*-phenylpiperidynyl or 1-(2-pyrimidyl)piperazinyl functional groups: (a) antidepressant trazodone, (b) anxiolytic buspirone, and (c) antipsychotic aripiprazole.

Understanding the thermodynamic parameters that govern CB[7] binding interactions can offer valuable insights into how structural modifications in ligand molecules influence their affinity for CB[7], which can, in turn, affect their pharmacological behavior. In this context, a series of experiments was conducted to evaluate the binding interactions between CB[7] and two different types of compounds: commonly used pharmaceuticals, trazodone, buspirone, and aripiprazole (Fig. 2), and a set of PhP and PyP derivatives (Fig. 1). All of these molecules contain a piperazine ring that exhibit p*K*_a_ ∼ 9,^100^ therefore, at physiological pH the nitrogen atoms are protonated and can establish hydrogen bonds and ion–dipole interactions with carbonyl groups of CB[7]. The analysis focuses on comparing the binding affinities, enthalpic and entropic contributions, and the influence of structural elements such as halogenation on the strength of the CB[7]–guest complexes.

The binding interactions between CB[7] and the pharmaceutical compounds trazodone, buspirone, and aripiprazole (Table 1) reveal distinct differences in affinity, largely influenced by the molecular characteristics of each drug. Trazodone exhibits the strongest binding affinity with a dissociation constant (*K*_D_) of 2.37 × 10^−6^ M, supported by a highly favorable free energy change (Δ*G* = −32.15 kJ mol^−1^). This interaction is characterized by a strongly exothermic enthalpic contribution (Δ*H* = −40.75 kJ mol^−1^), suggesting significant favorable interactions between trazodone and the CB[7] cavity, albeit with an unfavorable entropic contribution (−*T*Δ*S* = 8.56 kJ mol^−1^).

**Table 1:** Thermodynamic data for the association between CB[7] and trazodone, buspirone, and aripiprazole. All data were obtained *via* ITC at 298 K in 20 mM sodium acetate buffer (pH 3.0). Individual titration plots are provided in the ESI. Each titration was repeated at least three times, and the errors shown in the table were calculated from independent ITC measurements. *K*_D_ values were derived using a one-site binding model (*n* = 1). Due to the weak binding affinity of aripiprazole, it was not possible to fit all model parameters; thus, the *n* value was fixed at 1, and reliable determination of the enthalpic and entropic contributions could not be achieved

	*n* (sites)	*n* error (sites)	*K*_D_ (M)	*K*_D_ error (M)	Δ*G* (kJ mol^−1^)	Δ*H* (kJ mol^−1^)	Δ*H* error (kJ mol^−1^)	−*T*Δ*S* (kJ mol^−1^)
Trazodone	1.08	0.05	2.37 × 10^−6^	1.19 × 10^−7^	−32.15	−40.75	1.08	8.56
Buspirone	0.99	0.03	4.26 × 10^−6^	3.13 × 10^−7^	−28.70	−33.27	0.06	4.58
Aripiprazole	1.00	—	1.36 × 10^−2^	5.49 × 10^−3^	−10.83	—	—	—

Buspirone, which contains the structural fragment of 2a, exhibits a slightly weaker binding affinity to CB[7] (*K*_D_ = 4.26 × 10^−6^ M) with a Δ*G* of −28.70 kJ mol^−1^. The enthalpic contribution (Δ*H* = −33.27 kJ mol^−1^) is less exothermic than that of trazodone, indicating weaker interactions with CB[7]. Nevertheless, the interaction remains enthalpically driven, as reflected by a smaller entropic contribution, which is unfavorable (−*T*Δ*S* = 4.58 kJ mol^−1^).

In contrast, aripiprazole, which shares the 1e fragment, exhibits much weaker binding affinity with a *K*_D_ of 1.36 × 10^−2^ M and a corresponding Δ*G* of −10.83 kJ mol^−1^. Due to the weak binding, reliable determination of the enthalpic and entropic contributions was not possible. This weak binding may stem from the steric and electronic effects of the chlorine substituents on the PhP fragment, which, while leading to favorable interactions in some cases, appears to impede strong binding with CB[7] in the case of aripiprazole.

Turning to the PhP derivatives (1a–1i) and the PyP derivatives (2a–2b), the binding affinities span a range from −22.0 kJ mol^−1^ to −33.6 kJ mol^−1^ (Table 2). Compounds 1h and 1i exhibit the strongest affinities for CB[7], with *K*_D_ values of 1.34 × 10^−6^ M (Δ*G* = −33.6 kJ mol^−1^) and 1.75 × 10^−6^ M (Δ*G* = −32.9 kJ mol^−1^), respectively, indicating high binding affinities comparable to that of Trazodone. Notably, these compounds feature halogen substitutions on the aromatic ring. In contrast, weaker binding is observed for compounds such as 1f and 2a, with *K*_D_ values in the range of 10^−4^ M, underscoring the influence of halogen substitution on binding. For instance, 1f, with a *K*_D_ of 1.22 × 10^−4^ M (Δ*G* = −22.6 kJ mol^−1^), contains two chlorine substitutions at the 2- and 5-positions, which alter the volume of the aromatic moiety. Across the series, ligands bearing an *ortho*-F substituent exhibit the lowest free energies of binding (Δ*G*).

**Table 2:** Thermodynamic data for the association between CB[7] and selected PhP derivatives (1a–1i) and PyP derivatives (2a–2b). All data were obtained *via* ITC at 298 K in 20 mM phosphate–citrate buffer (pH 4.0). Individual titration plots are provided in the ESI. Each titration was repeated at least three times, and the errors shown in the table were calculated from independent ITC measurements. *K*_D_ values were derived using a one-site binding model (*n* = 1). Due to the weak binding affinity of guest 1f and 2a, it was not possible to fit all model parameters; thus, the *n* value was fixed at 1, and reliable determination of the enthalpic and entropic contributions could not be achieved

	*n* (sites)	*n* error (sites)	*K*_D_ (M)	*K*_D_ error (M)	Δ*G* (kJ mol^−1^)	Δ*H* (kJ mol^−1^)	Δ*H* error (kJ mol^−1^)	−*T*Δ*S* (kJ mol^−1^)
1a	1.00	0.01	2.07 × 10^−5^	9.55 × 10^−7^	−26.9	−15.2	2.6	−11.6
1b	0.94	0.09	1.17 × 10^−5^	1.60 × 10^−6^	−28.2	−10.2	0.1	−18.1
1c	0.96	0.05	1.72 × 10^−5^	1.67 × 10^−6^	−27.3	−21.9	1.3	−5.4
1d	0.98	0.05	2.16 × 10^−5^	7.07 × 10^−8^	−26.7	−21.6	0.6	−5.1
1e	0.93	0.10	8.39 × 10^−6^	2.66 × 10^−6^	−29.1	−37.4	1.7	8.3
1f	1.00	—	1.22 × 10^−4^	6.70 × 10^−5^	−22.6	—	—	—
1g	0.94	0.14	8.92 × 10^−6^	9.95 × 10^−7^	−28.9	−17.3	1.8	−11.6
1h	1.00	0.00	1.34 × 10^−6^	2.65 × 10^−8^	−33.6	−17.0	1.0	−16.6
1i	0.98	0.03	1.75 × 10^−6^	1.00 × 10^−7^	−32.9	−14.6	0.3	−18.3
2a	1.00	—	1.45 × 10^−4^	4.19 × 10^−5^	−22.0	—	—	—
2b	1.00	0.00	6.09 × 10^−5^	2.44 × 10^−5^	−24.2	−4.2	0.1	−20.0

The comparison between these two sets of data highlights the critical role of molecular structure in determining binding affinity. For all molecules containing the PyP and PhP fragments, halogenation—particularly fluorination—enhances binding affinity, likely due to favorable enthalpic interactions with CB[7], such as ion–dipole interactions, hydrogen bonding or halogen bonding involving the electronegative halogens. Nonetheless, the placement of halogen atoms can also diminish binding affinity, particularly when substituents are bulky or less polar. This is evident in compounds such as aripiprazole and 1f, which exhibit weaker binding, likely due to steric hindrance or reduced compatibility with the binding pocket, as reflected by their higher *K*_D_ values.

#### XRD structure

The crystal structures provide valuable insights into the hydrogen bonding interactions and binding modes of ligands within the CB[7] cavity. In all four crystal structures (1–4), the piperazine is monocationic with the aliphatic nitrogen protonated, forming N–H⋯O contacts to carbonyls at the CB[7] portal (Table 4). Two distinct ligand positions are observed, which result from the flexibility of the piperazine ring and its ability to adopt different conformations. For the PhP derivatives, the major and minor components exhibit refined occupancy factors of 0.6 and 0.4, respectively (Table 3). In the case of the 2a molecules, these occupancy factors are slightly different, at 0.8 and 0.2, respectively.

**Table 3:** Geometric parameters obtained for the major and minor positions of ligands 1a, 1h, 1i, and 2a from crystal structures 1–4, detailing the position of the positively charged nitrogen atom (N29). The atom names N29 and N30 represent the nitrogen atoms from the piperazine ring in the PhP and PyP derivatives, with N29 closer to the portal ring and N30 located deeper within the CB[7] cavity

	Occupancy	*d*(N29⋯Plane1)	∠(Cen1-Cen2-N29)	∠(Phenyl··Plane2)
**Major (yellow)**				
1a	0.6	0.09	5.33	61.87
1h	0.6	0.74	20.68	78.66
1i	0.6	0.19	16.97	83.13
2a	0.5	0.30	2.12	86.03

**Minor (pink)**				
1a	0.4	0.64	15.96	61.87
1h	0.4	1.36	1.00	78.66
1i	0.4	0.52	3.90	83.13
2a	0.5	0.31	6.78	85.98

**Fig. 3:**
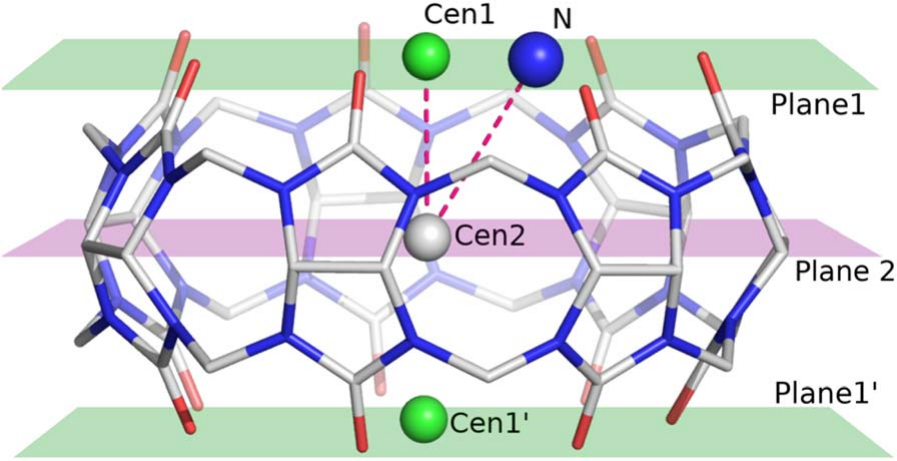
Visualization of calculated planes and centroids used to describe the features of ligand positions. Centroid 1 (Cen1) and plane 1 are calculated based on the upper oxygen atoms of CB[7], corresponding to the portal closer to the nitrogen atoms of the piperazine ring. Centroid 2 (Cen2) and plane 2 are calculated based on the equatorial carbon atoms of the CB[7] molecule.

One of the most significant interactions in these complexes is the ion–dipole interaction between the positively charged nitrogen atom of the piperazine ring and the dipole formed by the carbonyl groups of the CB[7] host. This interaction is a key factor in stabilizing the ligand within the CB[7] cavity. To facilitate binding pose analysis, several geometric parameters were defined (Fig. 3).

Among all ligand positions, the nitrogen atoms consistently occupy the middle of the portal to efficiently interact with the oxygen atoms of the carbonyl portals. As a result, the angle between Cen1, Cen2, and the >NH_2_^+^ (N29) nitrogen atoms is smaller than 20°, except in the case of 1h (Table 4). Another important parameter is the distance between the portal plane and the >NH_2_^+^ nitrogen atoms, which shows that the position of the nitrogen atom remains close to the portal plane for all ligands. An interesting observation is the difference in fluorine (1h) and chlorine (1i) substitution at the *para* positions of the phenyl ring. Fluorine, being smaller and more electronegative, fits easily within the CB[7] cavity, allowing for a larger variations in the distance between the <svg xmlns="http://www.w3.org/2000/svg" version="1.0" width="10.400000pt" height="16.000000pt" viewBox="0 0 10.400000 16.000000" preserveAspectRatio="xMidYMid meet"><metadata> Created by potrace 1.16, written by Peter Selinger 2001-2019 </metadata><g transform="translate(1.000000,15.000000) scale(0.011667,-0.011667)" fill="currentColor" stroke="none"><path d="M80 1160 l0 -40 40 0 40 0 0 -40 0 -40 40 0 40 0 0 -40 0 -40 40 0 40 0 0 -40 0 -40 40 0 40 0 0 -40 0 -40 40 0 40 0 0 -40 0 -40 40 0 40 0 0 -40 0 -40 40 0 40 0 0 80 0 80 -40 0 -40 0 0 40 0 40 -40 0 -40 0 0 40 0 40 -40 0 -40 0 0 40 0 40 -40 0 -40 0 0 40 0 40 -40 0 -40 0 0 40 0 40 -80 0 -80 0 0 -40z M560 520 l0 -40 -40 0 -40 0 0 -40 0 -40 -40 0 -40 0 0 -40 0 -40 -40 0 -40 0 0 -40 0 -40 -40 0 -40 0 0 -40 0 -40 -40 0 -40 0 0 -40 0 -40 -40 0 -40 0 0 -40 0 -40 80 0 80 0 0 40 0 40 40 0 40 0 0 40 0 40 40 0 40 0 0 40 0 40 40 0 40 0 0 40 0 40 40 0 40 0 0 40 0 40 40 0 40 0 0 80 0 80 -40 0 -40 0 0 -40z"/></g></svg> NH_2_^+^ nitrogen atom and the portal plane. In contrast, the larger chlorine atom with its different electrostatic potential distribution protrudes out of the CB[7] cavity. The distance between Portal Centroid 1′ and the chlorine atom is 2.150 Å, whereas for the fluorine atom at *para* position is only 1.490 Å, forcing the nitrogen atom of the piperazine ring to remain in the portal plane. The question remains what the optimal position of the ion is to obtain the most significant stabilization energy.

**Table 4:** Hydrogen Bonds for crystal structure 1–4

Crystal structure	D	H	A	*d*(D–A)/Å	D–H–A/°
1	O3W	H3WB	O6	2.82(1)	110.0
	N29A	H29D	O2W	2.91(1)	162.8
	N29B	H29F	O1	3.10(1)	177.0
2	N29A	H29F	O7	3.035(8)	126.4
	N29A	H29F	O1	3.031(9)	129.9
3	N29A	H29C	O6	3.12(2)	150.0
	N29	H29E	O1	3.94(2)	159.0
4	N29A	H29C	O4W	3.38(2)	146.5
	C43A	H43A	O2	3.15(2)	149.8
	N29B	H29E	O10	3.53(2)	111.1

As the piperazine ring undergoes conformational changes, it alters the hydrogen bonding patterns observed in the crystal structures. In the first structure, three prominent hydrogen bonds are identified. The first involves a water molecule, O3W–H3WB⋯O6, contributing to the stabilization of the system. The ligand's position is further stabilized by hydrogen bonds: N29A–H29D⋯O2W for conformer 1 and N29B–H29F⋯O1 for conformer 2. The O3W⋯O6 hydrogen bond, with a donor–acceptor distance of 2.82(1) Å, indicates a strong interaction. The ligand's first position is stabilized by a short hydrogen bond of 2.91(1) Å. The second position features an N29B⋯O1 hydrogen bond, with a distance of 3.10(1) Å and a favorable D–H–A angle of 177.7°, further contributing to the stabilization of the ligand.

In the second crystal structure, the key hydrogen bonds are N29A–H29F⋯O7 and N29A–H29F⋯O1, with donor–acceptor distances of 3.035(8) Å and 3.031(9) Å, respectively. The hydrogen atoms point toward the midpoint between the two oxygen atoms of the CB[7] portal. The second pose does not form classical hydrogen bonds, as the distance to closest O3 oxygen atom is 4.31(1) Å. The phenyl ring remains in a consistent position across these poses, while the piperazine ring adopts two orientations, suggesting flexibility in the ligand's interaction with CB[7].

In the third crystal structure, two hydrogen bonds are present: N29A–H29C⋯O6 and N29–H29E⋯O1. The N29A⋯O6 bond has a donor–acceptor distance of 3.12(2) Å and a D–H–A angle of 150.0°, indicating a relatively strong interaction. The second bond, N29⋯O1, with a distance of 3.94(2) Å, is significantly longer, implying that this interaction may be weaker or more transient in nature. The phenyl ring remains static, while the piperazine ring exhibits variability, with one orientation forming longer contacts and the N29 nitrogen atom positioned higher above the CB[7] portal plane.

The fourth crystal structure (Fig. 7) shows a unique hydrogen bonding pattern, with N29A–H29C⋯O4W and C43A–H43A⋯O2 as the key interactions. The N29A⋯O4W bond has a donor–acceptor distance of 3.38(2) Å. The position is also stabilized by C–H⋯π interaction. Additionally, a weaker interaction, N29B–H29F⋯O10, with a distance of 3.53(2) Å, suggests some degree of ligand flexibility in this structure as well. As in the previous structures, the nitrogen atom in the piperazine ring maintains ion–dipole interactions with the CB[7] carbonyl groups, playing a central role in maintaining the ligand's position within the host. This structure, like the others, shows multiple occupancy positions for the ligand, further emphasizing the dynamic nature of the interaction between CB[7] and the ligand. However, the ligand disorder in this structure is different, showing an 180° rotation.

Across all four structures, the hydrogen bonds are generally within typical bonding distances, with shorter donor–acceptor distances corresponding to stronger interactions. The nitrogen atom of the piperazine ring consistently plays a pivotal role in the binding by interacting with the carbonyl oxygens of CB[7], forming strong ion–dipole interactions that stabilize the ligands within the host cavity. The chlorine atom at the *para* position (1i) not only changes the ion–dipole interactions but also affects the orientation of the phenyl ring. The lack of substituents for 1a and 2a allows the ligands to change conformation within the cavity more freely, likely because there is enough space for such adjustments. In contrast, the substitution of a small fluorine atom forces the phenyl ring to adopt more perpendicular orientation, as indicated by the measured angle between the equatorial plane (plane 2) and the phenyl ring plane (78.66°). The chlorine atom, on the other hand, further increases this perpendicular orientation to 83.13°. Thus, the progression from H to F to Cl causes the phenyl ring to adopt a more perpendicular orientation, accommodating the increasing size of the substituent.

### Computational studies

The molecular interactions between CB[7] and a series of PhP and PyP derivatives have been studied from the thermodynamic and structural points of view. In the case of the thermodynamic data, it will be analyzed within the framework of Hostaš's relation and molecular dynamic simulations. In the case of the structural parameters, this will be complemented with results obtained from the SAPT and the QTAIM theory.

#### Theoretical Gibbs free energy

Hostaš's relation (eqn (1)) includes several terms, each with distinct physical significance.^104^ The first of these is interaction energy (eqn (3)). Notably, the CB[7]·1h and CB[7]·1i complexes exhibit some of the strongest interactions among the ligands studied, ranging from −271.3 to −278.2 kJ mol^−1^ (Table 5). In contrast, the interaction energies of other conformers are more widely distributed. This pronounced difference can be attributed to the presence of a halogen atom in the *ortho* position in 1h and 1i, which promotes stabilizing contacts and orients the ligand optimally within the CB[7] cavity. The *ortho*-fluorine substituent, in particular, engages in weak dispersion-type contacts with the hydrophobic wall, consistent with its inward orientation (Fig. 5 and 6). Such interactions are absent in the other systems, including 1a, which shows the weakest interaction energy in the series. To verify the robustness of these findings, various DFT functionals were tested (Table S4).

**Table 5:** The following table presents the various energy terms applicable to a given complex. Δ*E*^TPSS0/cc-pVTZ^_int_ is the interaction energy, Δ*E*_disp_ is the SAPT dispersion energy, Δ*G*_solv_ is the desolvation free energy, *T* is the temperature, Δ*S*_APR_ is the entropy obtained from APR method, Δ*E*_def_ is the deformation energy for the host and guest, Δ*G*_exp_ is the experimental Gibbs free energy obtained from ITC studies, Δ*G*_calcd_ is the Hostaš’s Gibbs free energy. All the energy values are in [kJ mol^−1^] except *T* is in [K] and Δ*S* in [kJ mol^−1^ K^−1^]

Complex	Conformer	Δ*E*^TPSS0/cc-pVTZ^_int_	Δ*E*_disp_	Δ*G*_solv_	−*T*Δ*S*_APR_	Δ*E*_def_ (host)	Δ*E*_def_ (guest)	Δ*G*_calcd_	Δ*G*_exp_
CB[7]·1a	1	−228.3	−180.4	268.6	40.6	130.1	10.1	40.7	−27.5
CB[7]·1a	2	−270.7	−169.6		−64.4	146.9	23.4	−65.7	
CB[7]·1b	—	−268.1	−174.6	281.3	−18.2	137.0	14.7	−27.9	−23.2
CB[7]·1h	1	−274.2	−173.9	262.5	−52.4	145.6	19.0	−73.4	−33.6
CB[7]·1h	2	−277.6	−177.8		−39.9	145.8	18.6	−68.3	
CB[7]·1i	1	−278.2	−181.3	265.4	−74.4	145.2	19.0	−104.3	−32.9
CB[7]·1i	2	−271.3	−178.7		−53.7	136.8	15.0	−86.4	
CB[7]·2a	1	−283.2	−154.4	267.7	−18.9	145.5	21.5	−21.9	−21.4
CB[7]·2a	2	−281.3	−157.7		−0.6	158.8	21.9	8.9	

**Fig. 4:**
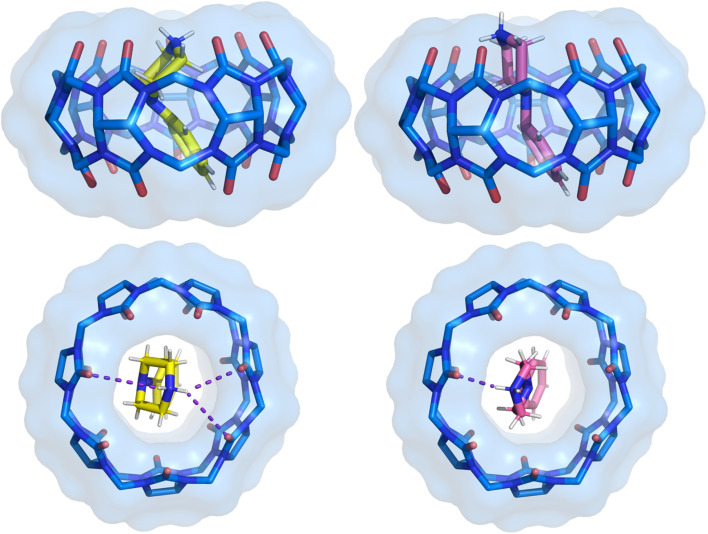
Side and top view of the binding pose from crystal structure 1 with separated two positions of the 1a ligand. The ligand pose shown with carbon atoms in yellow has a refined occupancy factor of approximately 0.6, while the second pose has an occupancy factor of approximately 0.4. The phenyl ring position remains the same, with only the piperazine ring showing variation. CB[7] is shown as sticks with a van der Waals surface. Water molecules and hydrogen atoms of CB[7] have been removed for clarity.

**Fig. 5:**
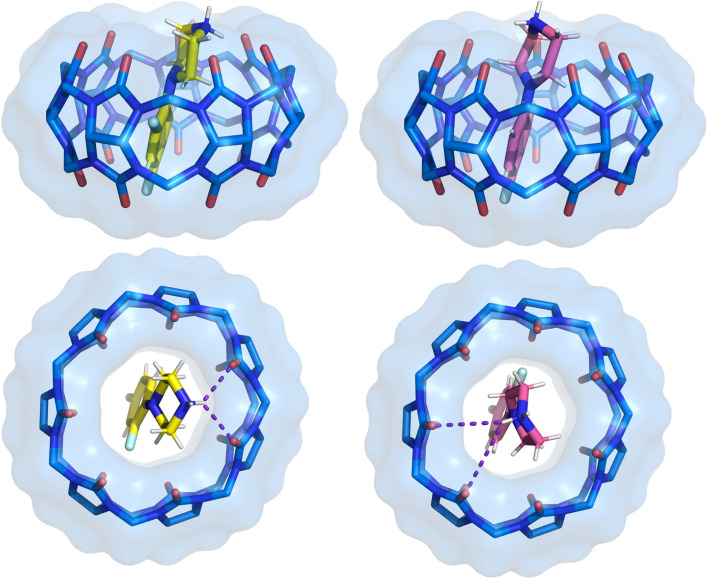
Side and top view of the binding pose from crystal structure 2 with separated two positions of the 1h ligand. The ligand pose shown with carbon atoms in yellow has a refined occupancy factor of approximately 0.6, while the second pose has an occupancy factor of approximately 0.4. CB[7] is shown as sticks with a van der Waals surface. Water molecules and hydrogen atoms of CB[7] have been removed for clarity.

**Fig. 6:**
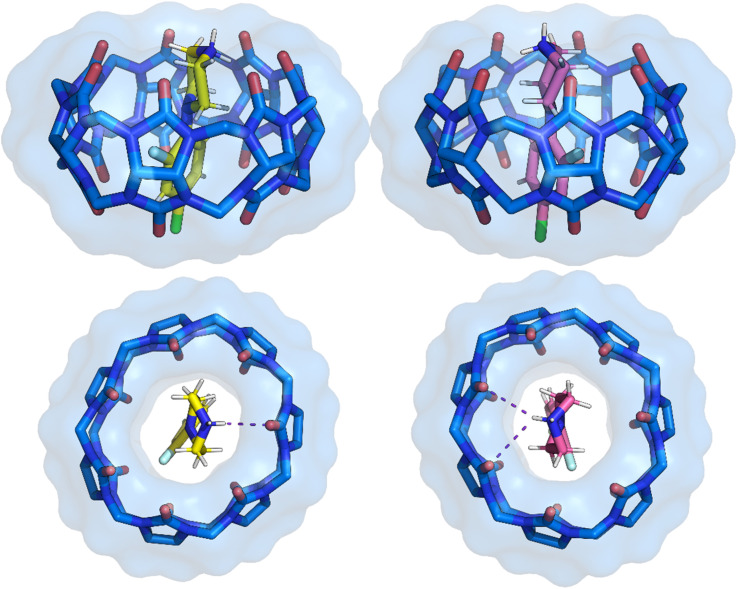
Side and top view of the binding pose from crystal structure 3 with separated two positions of the 1i ligand. The ligand pose shown with carbon atoms in yellow has a refined occupancy factor of approximately 0.6, while the second pose has an occupancy factor of approximately 0.4. The phenyl ring position remains the same, with only the *N*-phenylpiperazine ring showing variation. CB[7] is shown as sticks with a van der Waals surface. Water molecules and hydrogen atoms of CB[7] have been removed for clarity.

**Fig. 7:**
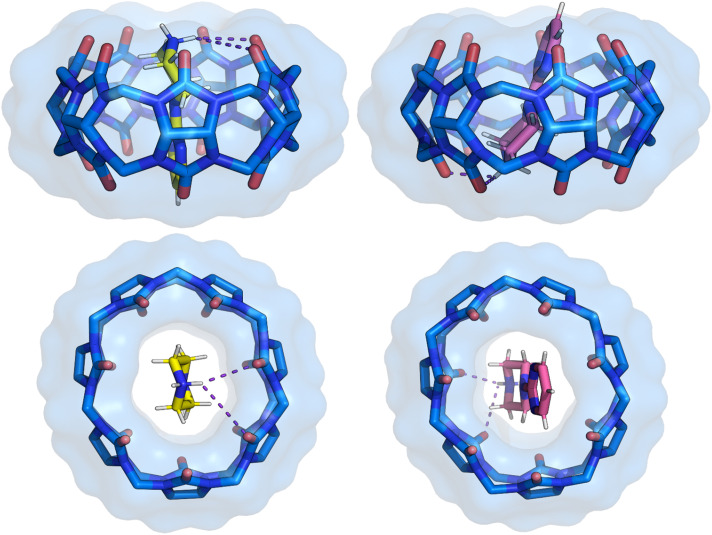
Side and top view of the binding pose from crystal structure 4 with separated two positions of the 2a ligand. The ligand pose shown with carbon atoms in yellow has a refined occupancy factor of approximately 0.5, while the second pose has an occupancy factor of approximately 0.5. The phenyl ring position remains the same, with only the *N*-phenylpiperazine ring showing variation. CB[7] is shown as sticks with a van der Waals surface. Water molecules and hydrogen atoms of CB[7] have been removed for clarity.

The second term in eqn (1) corresponds to the dispersion energy, which arises from the instantaneous correlation of fluctuating multipole moments between the host and guest—resulting in attractive London dispersion forces. This term was calculated using the SAPT approach. As shown in Table 5, the most favorable dispersion interactions are observed in the CB[7]·1i conformers, with values exceeding −178.7 kJ mol^−1^. This can be attributed to the presence of halogen atoms in these complexes (Fig. 6), which enhance dispersion interactions.

Interestingly, there is a notable correlation between the bound ligand conformation and the magnitude of the dispersion energy. For instance, conformer 1 of CB[7]·1a displays a significantly stronger dispersion energy of −180.4 kJ mol^−1^. Although this ligand lacks halogen atoms, its conformation promotes close contact with the CB[7] cavity (Fig. 4), thereby maximizing London dispersion interactions.

The third term in eqn (1) represents the free energy of desolvation, calculated using the COSMO-RS method. This term reflects the energetic penalty for transferring the ligand from aqueous solution into the hydrophobic CB[7] cavity.^80^ In this context, ligand 1b exhibits the least favorable desolvation energy (Table 5). However, no clear trend was observed across the set, and the obtained values are consistent with those reported in the literature.^80^

The fourth term is associated with variations in entropy and temperature. This term can be understood as the difference in the number of degrees of freedom of the ligand in CB[7] cavity *versus* in bulk solution. An illustration of flexibility loss is evident in both the ligand and the CB[7] system in solution. This is observed in conformer 1 of CB[7]·1a, which has a positive −*T*Δ*S* term of 40.6 kJ mol^−1^ K^−1^ (Fig. 4). This indicates that the conformer exhibits higher flexibility in the solvent but becomes locked in the CB[7] cavity upon binding. In contrast, conformer 2 in the same complex has a stabilizing entropy term (−64.4 kJ mol^−1^ K^−1^), indicating that this conformer does not lose its conformational freedom upon binding with CB[7], unlike conformer 1. Similarly, the conformers of 1h and 1i exhibit favorable entropic term values, ranging from −39.9 to −74.4 kJ mol^−1^ K^−1^. This suggests that ligands 1h and 1i also retain their conformational freedom in the CB[7] cavity. The observed rigidity of the second conformer in ligands 1h and 1i inside CB[7] is attributed to fluorine contacts with the hydrogen of the piperazine ring. Consequently, the displayed −*T*Δ*S* term for the second conformer of both ligands is more positive in comparison with the first conformer. In these ligands, intramolecular C–H⋯F interactions restrict conformational flexibility in solution, so fewer degrees of freedom are lost upon binding. This pre-organization reduces the entropic penalty relative to more flexible ligands such as 1a.

The final two terms of eqn (1) represent the deformation energy *i.e.* the difference in conformational energy of the host and the guest upon complex formation. The deformation energy of the host is greater than that of the ligands. This is a consequence of the CB[7] molecule undergoing a change in shape around the ligands. Furthermore, in concordance to the entropy values obtained for conformer 1 of CB[7]·1a, the deformation energy of 1a is the lowest among the compounds under consideration. This is a consequence of the molecule's rigidity. In comparison with other ligands, which exhibit larger values (Table 5).

**Fig. 8:**
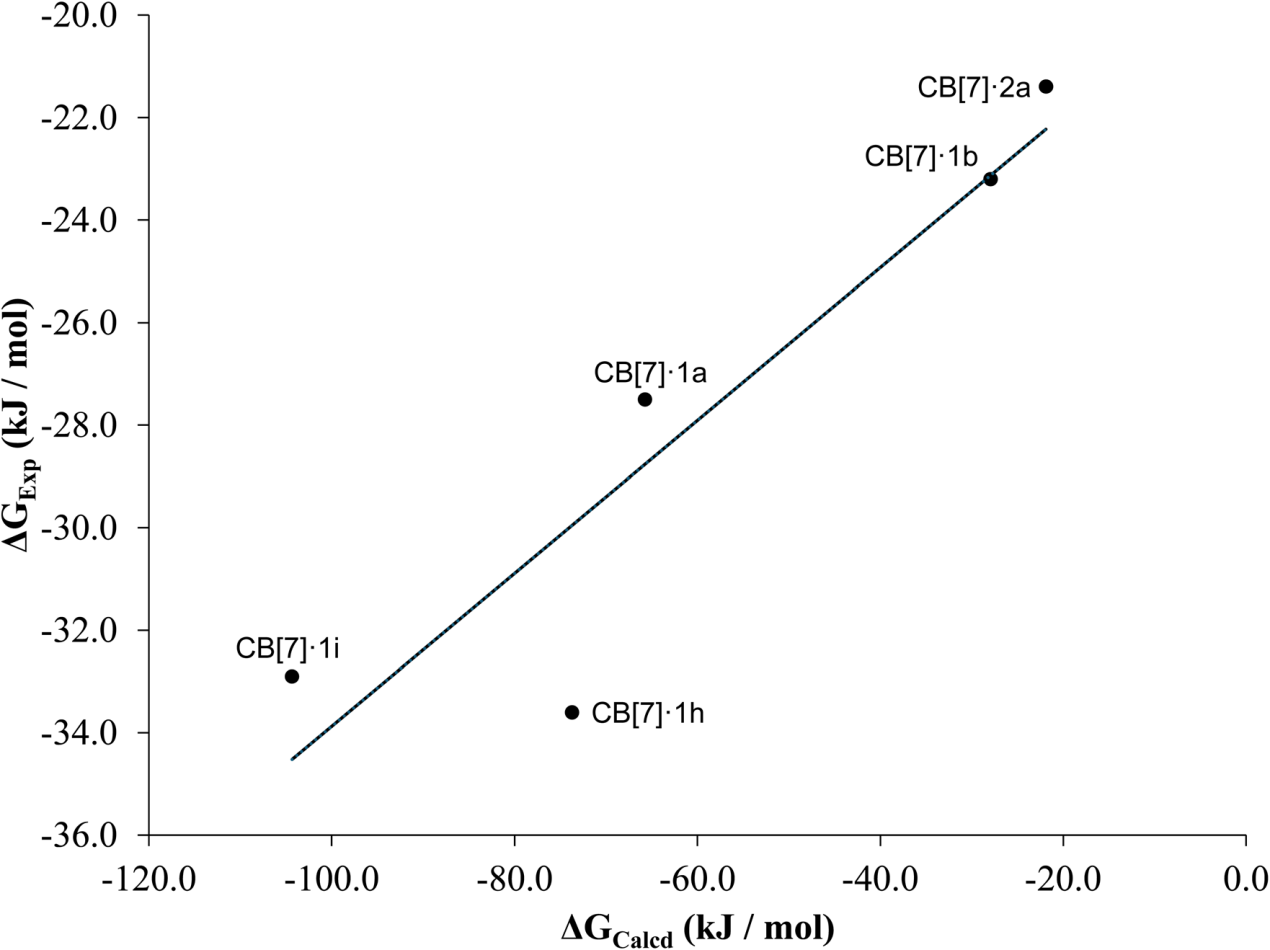
Experimental Gibbs free energy *versus* estimated Gibbs free energy based on eqn (1). The equation for the adjusted tendency (continuous line) is *Y* = 0.1491× −18.9618 and *R*^2^ = 0.85.

The calculated binding free energy (Δ*G*_calcd_) was obtained by applying eqn (2) followed by eqn (1), with the resulting values summarized in Table S5. These values were then correlated with the experimental binding free energies Δ*G*_exp_, yielding a reasonably strong correlation coefficient of 0.85 (Fig. 8). This agreement supports the reliability of the computational approach. This trend indicates that CB[7]·1h and CB[7]·1i are the most stable complexes that can be formed as it can be seen by their *K*_D_ values (Table 2).

#### SAPT interaction energy

**Fig. 9:**
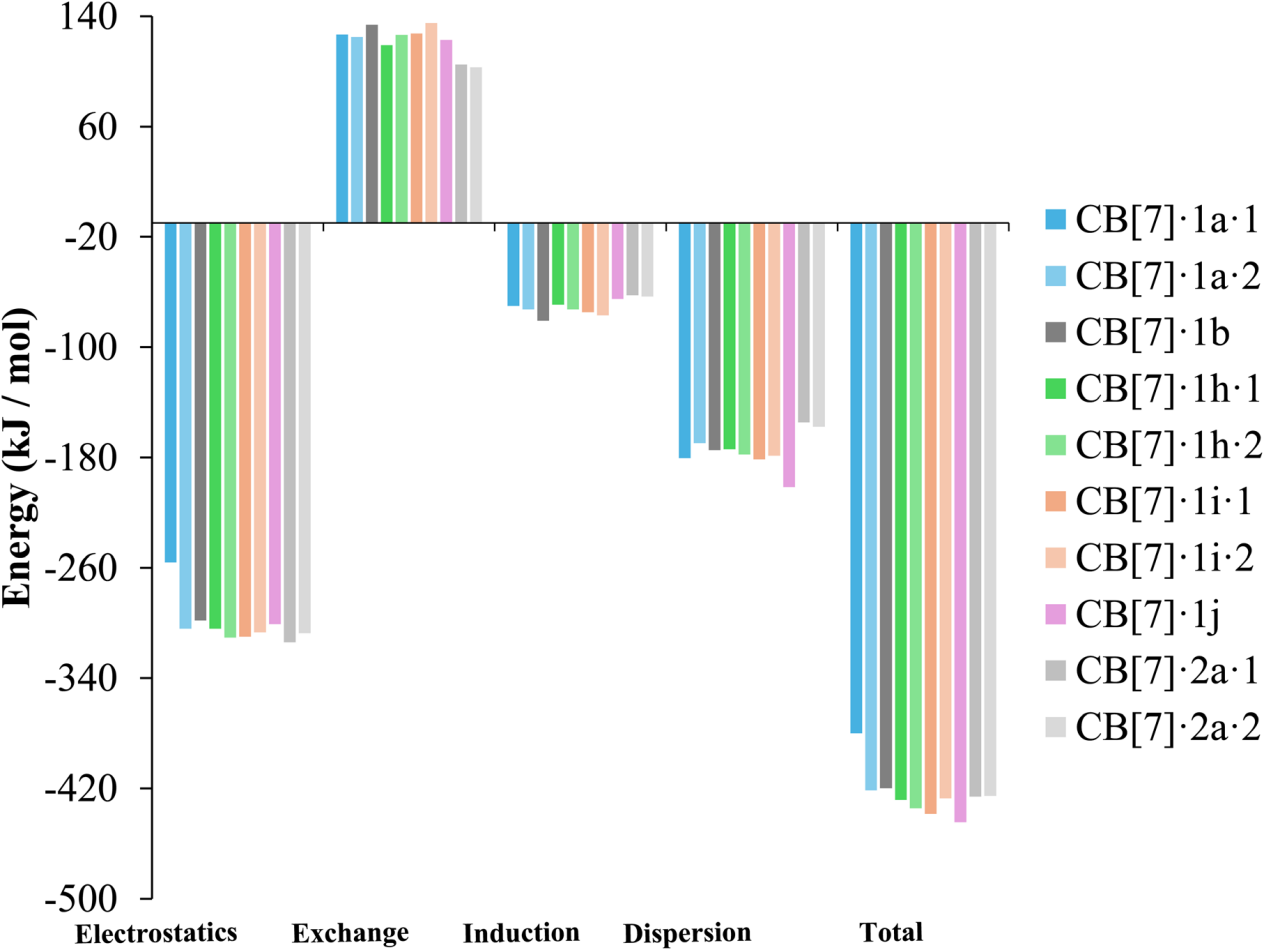
SAPT interaction energy components for each different complex ligand conformer.

SAPT analysis (Fig. 9, Table S6) shows that CB[7]–guest interactions are dominated by electrostatics and dispersion. Electrostatics arise from ion–dipole interactions and hydrogen bonding between the protonated piperazine groups and the carbonyl-lined portals of CB[7], which act as an anchor for all complexes. Dispersion makes the second-largest stabilizing contribution, particularly in halogenated ligands where London dispersion forces reinforce binding. At the *ortho* position, both F and Cl enhance dispersion; Cl yields a more stabilizing (more negative) dispersion term than F. Induction terms are comparatively minor but more favorable for phenyl than pyrimidine scaffolds. Overall, fluorinated ligands (1h, 1i) gain additional stabilization from electrostatics when the *ortho*-fluorine atom is oriented toward the cavity, while bromine-substituted ligands benefit from their greater polarizability, consistent with stronger dispersion and induction contributions. Full SAPT energy decompositions for each complex are provided in Table S6.

#### Molecular dynamics

APR free energy calculations (Δ*G*_APR_)^64,101,102^ confirm that complexation of the studied ligands with CB[7] is predominantly enthalpy-driven (Table S7). Among the studied complexes, CB[7]·1h and CB[7]·1i exhibit the most favorable enthalpy contributions (<−270.0 kJ mol^−1^), and Gibbs free energy (<−310.0 kJ mol^−1^), in line with their strong experimental affinities and the interaction energy analysis. This stability reflects a dual effect: pre-organization *via* intramolecular C–H⋯F contacts lowers the entropic penalty, while the *ortho*-fluorine substituents also enable additional weak but cooperative contacts in the CB[7] cavity.

**Fig. 10:**
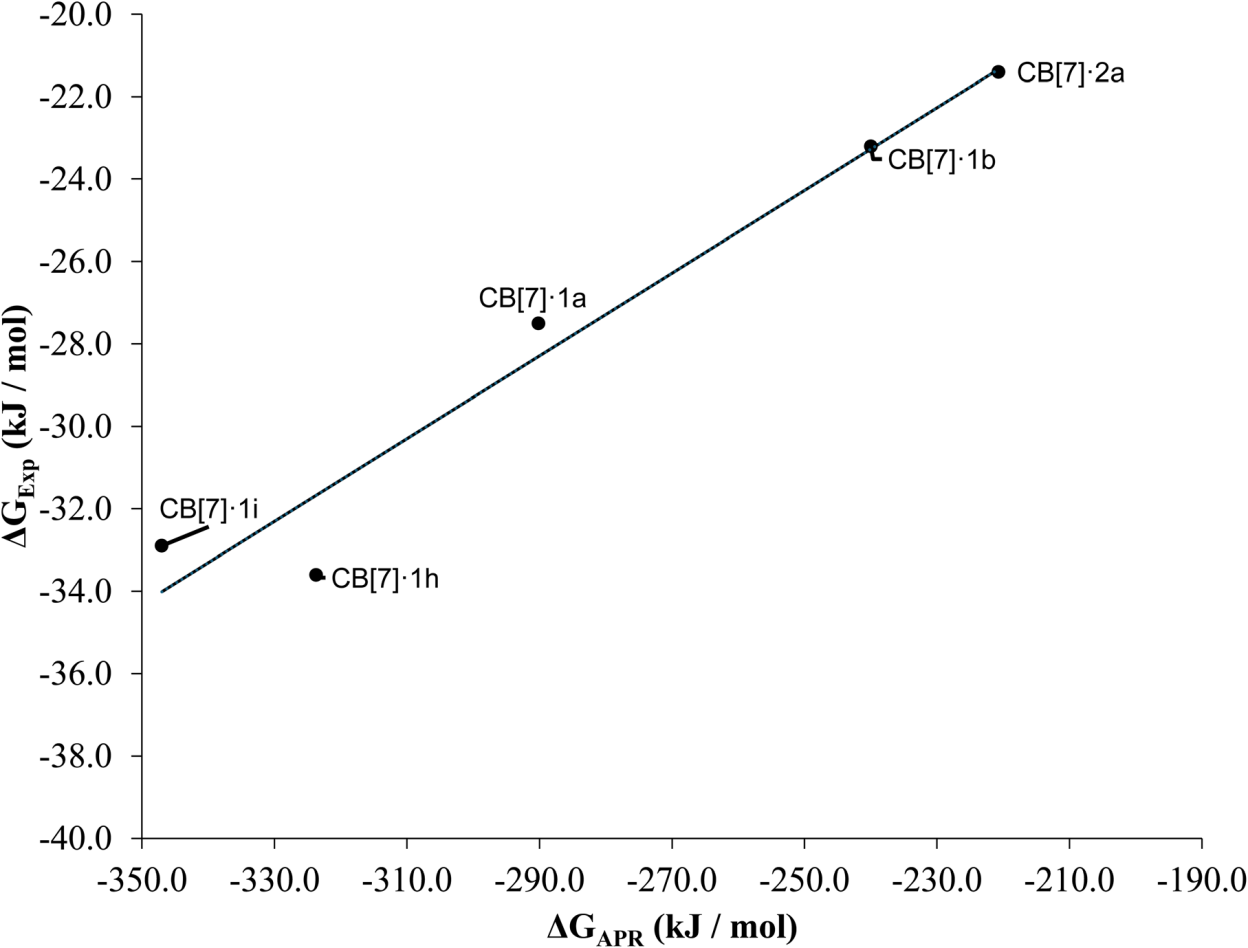
Experimental Gibbs free energy *versus* APR Gibbs free energy. The equation for the adjusted tendency (continuous line) is *y* = 0.1004*x* + 0.8343 and *R*^2^ = 0.95.

More flexible ligands in solution such as 1a display less favorable binding free energies due to larger entropic costs upon complexation. Notably, the APR-derived free energies show excellent correlation with ITC results (*R*^2^ = 0.95, Fig. 10), supporting the robustness of the method.

#### Inter-molecular interactions

QTAIM analysis identified nearly 200 intermolecular interactions across all complexes, classified into hydrogen bonds, weak fluorine contacts, and *n* → π* interactions. Classification was performed using two complementary approaches: one based on geometric and energetic criteria proposed by Jeffrey^103^ and the other on electronic parameters as described by Hayashi^98^ (Tables 6 and 7, S9 and S10).

**Table 6:** Geometrical parameters, QTAIM properties at the bond critical points (in au ×10^3^), *E*(*r*_c_) is in kJ mol^−1^, and classification of all intramolecular hydrogen bonds. GC is geometric classification, EC corresponds to energy classification and HC stands for Hayashi classification. The number in square brackets corresponds to the glycoluril unit in CB[7]. pCS stands for pure Closed-Shell and rCS is regular Closed Shell

Complex	Conformer	Bond	∠(X–H⋯X)	*d*(Å)	*ρ*(*r*_c_)	*∇* ^2^ *ρ*(*r*_c_)	*V*(*r*_c_)	*H*(*r*_c_)	*E*(*r*_c_)	GC	EC	HC
CB[7]·1a	1	[O1]1⋯HN4	89.53	2.42	9.04	35.62	−5.20	1.86	5.12	Weak	Weak	pCS
CB[7]·1a	1	[O1]2⋯HN4	126.94	2.42	9.17	36.18	−5.30	1.89	5.18	Weak	Weak	pCS
CB[7]·1a	2	[O1]1⋯HN4	171.27	1.90	26.17	89.78	−21.70	0.40	17.01	Moderate	Moderate	pCS
CB[7]·1b	—	[O1]1⋯HN4	178.53	1.88	28.24	92.25	−23.90	−0.40	18.63	Moderate	Moderate	rCS
CB[7]·1h	1	[O1]1⋯HN4	145.56	2.15	15.02	59.24	−9.70	2.55	8.39	Moderate	Weak	pCS
CB[7]·1h	2	[O1]1⋯HN4	147.88	2.05	19.18	72.46	−13.50	2.33	11.09	Moderate	Weak	pCS
CB[7]·1i	1	[O1]1⋯HN4	150.21	2.03	20.02	74.35	−14.30	2.14	11.71	Moderate	Weak	pCS
CB[7]·1i	2	[O1]1⋯HN4	160.34	1.95	23.63	83.73	−18.40	1.25	14.68	Moderate	Weak	pCS
CB[7]·2a	1	[O1]1⋯HN4	145.48	2.16	15.01	58.29	−9.60	2.48	8.32	Moderate	Weak	pCS
CB[7]·2a	2	[O1]1⋯HN4	141.83	2.15	15.64	60.81	−10.10	2.57	8.65	Moderate	Weak	pCS

**Table 7:** Geometrical parameters, QTAIM properties at the bond critical points (in au ×10^3^), *E*(*r*_c_) is in kJ mol^−1^, and classification of all fluorine contacts. EC corresponds to energy classification and HC stands for Hayashi classification. The number in square brackets corresponds to the glycoluril unit in CB[7]. *e* stands for equatorial. pCS stands for pure closed-shell

Complex	Conformer	Bond	∠(X–H⋯X)	*d*(Å)	*ρ*(*r*_c_)	*∇* ^2^ *ρ*(*r*_c_)	*V*(*r*_c_)	*H*(*r*_c_)	*E*(*r*_c_)	EC	HC
CB[7]·1h	1	[O1]3⋯FC2′	116.4	3.063	6.2	26.9	−4.2	1.3	4.4	Weak	pCS
CB[7]·1h	1	[O1]4⋯FC2′	93.6	3.146	5.4	23.4	−3.6	1.1	4.0	Weak	pCS
CB[7]·1h	1	C2′F⋯HeC2	95.3	2.178	15.8	73.2	−11.6	3.4	9.7	Weak	pCS
CB[7]·1h	2	[O1]3⋯FC2′	107.6	3.181	4.9	20.3	−3.2	1.0	3.7	Weak	pCS
CB[7]·1h	2	[C3]4⋯FC2′	113.1	2.940	6.8	30.7	−5.0	1.4	5.0	Weak	pCS
CB[7]·1h	2	C2′F⋯HeC2	92.1	2.239	14.0	64.2	−10.1	3.0	8.7	Weak	pCS
CB[7]·1i	1	[O1]3⋯FC2′	107.6	3.187	4.8	20.1	−3.1	1.0	3.6	Weak	pCS
CB[7]·1i	1	[C3]4⋯FC2′	113.5	2.924	7.0	31.6	−5.2	1.4	5.1	Weak	pCS
CB[7]·1i	1	C2′F⋯HeC2	92.8	2.216	14.6	67.6	−10.6	3.1	9.1	Weak	pCS
CB[7]·1i	2	[O1]5⋯FC2′	107.3	3.158	5.5	24.0	−3.7	1.1	4.1	Weak	pCS
CB[7]·1i	2	[O1]6⋯FC2′	96.8	3.214	4.6	19.7	−3.0	0.9	3.6	Weak	pCS
CB[7]·1i	2	C2′F⋯HeC6	94.1	2.236	14.0	64.3	−10.1	3.0	8.7	Weak	pCS

The majority are weak hydrogen bonds (Table 6) involving the piperazine scaffold, typically electrostatic^104^ in nature and closed-shell according to topological criteria. A few stronger hydrogen bonds (notably in CB[7]·1b) display partial covalent character, but most remain in the weak-to-moderate range. The remaining hydrogen bonds can be characterized as dispersion-like interactions^105^ (Table S9).

Fluorine-based interactions^107,108^ were observed exclusively in the *ortho*-fluorinated ligands (1h, 1i), confirming their unique behavior. These contacts between the ligands and the inner part of CB[7] are long (>3.0 Å) and weak (*ρ*(*r*_c_) < 0.01 au, *E*(*r*_c_) ≈ 4–6 kJ mol^−1^), consistent with dispersion-dominated closed-shell interactions rather than classical halogen bonds (Fig. S18–S24).^109,110^ Intramolecular C–H⋯F contacts^106^ (Table 7), however, contribute to pre-organization in solution, lowering the entropic penalty of binding. As it was observed in the second conformers of CB[7]·1h and CB[7]·1i (Table 5).

*n* → π* interactions^111^ between the aromatic scaffolds and carbonyl oxygen atoms were also detected (typically three per complex), contributing weak dispersion-like stabilization.^112^

In summary, QTAIM reveals that binding stabilization arises from a dense network of weak interactions^113^—electrostatic hydrogen bonds at the portals, fluorine contacts and *n* → π* contacts inside the cavity, and intramolecular C–H⋯F pre-organization for *ortho*-fluorinated ligands. Taken together with the APR and SAPT analyses, these results converge with ITC experiments to provide a consistent interpretation: the stability of *ortho*-fluorinated complexes originates primarily from ligand pre-organization, complemented by cooperative networks of weak non-covalent interactions inside CB[7].

## Discussion

CB[7] molecules form inclusion complexes with drug molecules and small molecules bearing PhP and PyP fragments, owing to hydrogen bonding interactions with the carbonyl oxygens portals of CB[7]. Although stabilizing, these hydrogen bonds do not rigidly constrain the piperazine ring, allowing for shifts in its puckering conformation. In the crystal structures, at least two orientations of the piperazine ring are observed, both of which form stabilizing hydrogen bonds. The strongest hydrogen bonds are formed between the hydrogen atom of the positively charged amine group on the piperazine scaffold and the carbonyl group of CB[7] (Tables 4 and 6). These bonds are essential for ensuring the guest molecule remains inside the host (Tables S6, S7 and Fig. 9). This interaction is classified as an ionic hydrogen bond due to the electron density at the bond critical point not being fully localized (Table 6), following the Cremer–Kraka definition.^97^

A conserved feature among the ligands in the crystal structures is the phenyl ring position. The torsion angle with the CB[7] equatorial plane becomes increasingly perpendicular with larger substituents. Substitution at the *para* position of the phenyl ring exerts different effects depending on the halogen's size. Compared with the unsubstituted compound (1a), bromine substitution (1b) leads to a loss in binding free energy, whereas the iodine *para* substituent (1c) provides a favorable enthalpic contribution (ΔΔ*H*(1a–1c) = −6.7 kJ mol^−1^). It appears that bromine is too small to form additional stabilizing contacts, whereas the larger iodine atom probably forms halogen bonds and lowers the enthalpy during complex formation. This also explains one of the smallest entropic term contribution observed for 1c, suggesting that iodine is “stuck” in the portal.

A similar stabilizing entropic contribution is observed for fluorine at the *meta* position (1d), which also lowers the enthalpy (ΔΔ*H*(1a–1d) = −6.4 kJ mol^−1^). This indicates that the fluorine atom forms stabilizing interactions without hindering its rotational freedom within the cavity. A parallel observation emerges from comparing derivatives bearing two chlorine atoms on the phenyl ring (1e, 1f, and 1g): compounds with chlorine atoms in the *meta* position show the most advantageous enthalpy among the tested compounds, suggesting that this arrangement is crucial for stabilizing noncovalent interactions. Notably, 1e exhibits the lowest Δ*H* (−37.4 kJ mol^−1^); however, this is offset by a positive entropic term (−*T*Δ*S* = +8.3 kJ mol^−1^)—the only positive value observed. Slightly less but still favorable enthalpic contributions are seen for 1h and 1i, consistent with interaction energy calculations (Tables 2 and 5), which both parameters have a correlation coefficient of 0.80 (Fig. S25). The stability of these ligands (1h and 1i) arises from a combination of effects: (i) reduced entropic penalty due to intramolecular C–H⋯F contacts that pre-organize the ligand in solution, and (ii) additional weak cooperative contacts inside CB[7] (dispersion, *n* → π, occasional F⋯H/O). These interactions are individually weak but act collectively to support the observed high stability, rather than resulting from a single strong fluorine–cavity interaction. Consequently, CB[7] deforms from circular to elliptical to accommodate these ligands within its structure.

A key observation is that a fluorine atom at the *ortho* position (1h, 1i) lowers the entropic penalty of binding which is consistent with previous findings in ligand–protein complexes.^48^ Intramolecular C–H⋯F contacts already restrict the conformational space of these ligands in solution, effectively pre-organizing them for complexation. To quantify this, we compared rotational profiles of 1i and 1j, which show a stabilization for 1i of ∼5 kJ mol^−1^ (Fig S22). As a result, fewer degrees of freedom are lost upon binding, which manifests as a more favorable –*T*Δ*S* term. Furthermore, the mean distance between the fluorine atoms at the PhP scaffold and CB[7] is 3.083(5) Å for 1h and 3.120(8) Å for 1i, consistent with weak, long-range contacts. The difference arises from the phenyl ring orientations, which must adopt a more perpendicular arrangement when a chlorine atom is located at the *para* position.

In certain instances, the conformational preference of the ligand is not thermodynamically favored for complex formation in comparison with the other ligands studied herein. This phenomenon is illustrated by conformer 1 of ligand 1a (Fig. 4, Tables S6 and S7). This molecule is characterized by its insolubility in water, and its propensity to bind to CB[7] due to the presence of strong London dispersion forces (Table S6). Conversely, the second conformer of the same ligand exhibits a more favorable interaction energy, thereby stabilizing the complex formation (Tables 2, 5, S6 and S7), and this conformational preference allows it to form intermolecular interactions between this ligand within CB[7] (Tables 6, S9, and S10). The observed differences in these conformers can be attributed to the preferential conformation of the ligand (Tables S6 and S7). A comparison of this trend with the experimental results (Table 2) reveals that its binding strength, *K*_D_, is in the middle of the rest dividing the bunch on the strong and weaker binding ligands.

Furthermore, the computational thermodynamic data corroborates experimental observations concerning fluorine contacts. The majority of complexes that have a fluorine atom in the *ortho* position are the most stable (Tables 2, 5, S6, and S7). This phenomenon can be attributed to an intermolecular weak fluorine contacts. Specifically, intramolecular fluorine contacts in 1h and 1i restrict conformations already in solution, lowering entropy loss on binding. This pre-organization also favors additional weak contacts with CB[7] (Fig. 9 and S18–S24), which, while modest in strength, complement the entropy effect. This finding underscores the pivotal role of fluorine atoms in *ortho* positions in the design of novel ligands for achieving robust interactions with CB[7].

The underlying rationale for the stability of these complexes can be elucidated through a comprehensive examination of the interactions. A detailed examination will be conducted on CB[7]·1a, CB[7]·1h, and CB[7]·1i. In both conformers of 1a, the presence of non-typical hydrogen interactions like the dihydrogen bond between the piperazine and aromatic scaffolds have been observed (Table S9). These intramolecular interactions restrict the accessible conformational space for these conformers. Furthermore, consistent with the conformational preferences of 1a (Table 5), the conformer 1 exhibits reduced flexibility. Consequently, it can be observed that this ligand displays a reduced propensity to form intermolecular hydrogen bonds within CB[7] in comparison to its counterpart, the second conformer (Table S9). Conversely, within the complexes of CB[7]·1h and CB[7]·1i, there is a concordance between the preferred conformations (Table 5) and the favorable intramolecular fluorine contacts (Table 7). This pre-organization allows 1h and 1i to engage in a wider array of intermolecular interactions with CB[7], including hydrogen bonds (Table S9), fluorine contacts (Table 7 and Fig. S18–S24), and *n* → π* interactions (Table S10). While individually weak, these contacts act cooperatively and complement the reduced entropic penalty from ligand pre-organization.

Our measured affinities (micromolar range) are modest compared to benchmark CB[7] systems (*e.g.*, adamantane, ferrocene). Rather than record-breaking binders, this study provides mechanistic insight into halogen effects in drug-like scaffolds. *Ortho*-fluorine derivatives (1h, 1i) illustrate how pre-organization reduces entropy loss, complemented by weak cavity contacts. These findings show that even moderate–affinity complexes can reveal principles for rational cucurbituril–guest design.

## Conclusions

This work provides an integrated thermodynamic, crystallographic, and computational analysis of cucurbit[7]uril complexes with halogenated phenylpiperazine and pyrimidylpiperazine derivatives. By combining ITC, single-crystal X-ray diffraction, and molecular simulations (APR, SAPT, QTAIM), we examined how halogen substitution influences host–guest stability through both enthalpic and entropic contributions.

Among the ligands studied, *ortho*-fluorinated derivatives (1h, 1i) display the highest stability. Their favorable binding arises primarily from intramolecular C–H⋯F contacts that pre-organize the ligand in solution, thereby reducing the entropic penalty of complexation. Inside the CB[7] cavity, these ligands also form a cooperative network of weak contacts (dispersion, *n* → π*, and occasional F⋯H/O interactions), which further support binding. Other halogen substituents contribute mainly through enthalpic effects related to size and polarizability, with iodine showing the strongest tendency toward halogen-bond-like interactions.

Taken together, these findings highlight how even moderate–affinity complexes can reveal valuable mechanistic principles of cucurbituril recognition. The convergence of experimental thermodynamics, crystallographic evidence, and computational analysis provides a consistent picture of how halogen substitution—and particularly *ortho*-fluorine pre-organization—modulates the balance of enthalpy and entropy in host–guest binding.

## Author contributions

Conceptualization: M. M. Methodology: M. M. and D. R. Investigation: M. M., E. Z. and D. R. Visualization: M. M. and D. R. Funding acquisition: M. M. Project administration: M. M. Supervision: M. M. Writing – original draft: M. M. and D. R. Writing – review & editing: M. M. and D. R.

## Conflicts of interest

There are no conflicts to declare.

## Supplementary Material

RA-015-D5RA07259J-s001

RA-015-D5RA07259J-s002

## Data Availability

CCDC 2468857–2468859, and 2469564 (1, 2, 3, and 4) contain the supplementary crystallographic data for this paper.^114*a*–*d*^ All data needed to evaluate the conclusions in the paper are present in the paper and/or the supplementary information (SI). Supplementary information is available. See DOI: https://doi.org/10.1039/d5ra07259j.
