# Supplementary material for: Halogenated N-phenylpiperazine and 2-(piperazin-1-yl)pyrimidine as novel cucurbit[7]uril guests: experimental and computational insights into supramolecular binding
Source: RSC Adv. 2025 Nov 20;15(53):45555–72. doi: 10.1039/d5ra07259j (PMC12632625; doi:10.1039/d5ra07259j)
Supplement: RA-015-D5RA07259J-s001 [file RA-015-D5RA07259J-s001.pdf]

Supplementary Information (SI) for RSC Advances.
This journal is © The Royal Society of Chemistry 2025

Halogenated N-Phenylpiperazine and 2-(Piperazin-1-yl)pyrimidine as Novel Cucurbit[7]uril Guests: Experimental and Computational Insights into Supramolecular Binding

David A. Rincón[1], Ewelina Zaorska[1] and Maura Malinska[1]

[1]Faculty of Chemistry, University of Warsaw, Pasteura1, 02-093, Warsaw, Poland

Supplementary Information

Contents

Fig. S1 Results of two selected isothermal titration calorimetry (ITC) experiments, showing the raw heat release data and the integrated heat released per injection for the titration of CB7 by Trazodone at 298 K in sodium acetate buffer (pH 3.0). The data points represent the experimental values, while the fitted curve corresponds to the best-fit binding model used for thermodynamic analysis. 6
Fig. S2 Results of two selected isothermal titration calorimetry (ITC) experiments, showing the raw heat release data and the integrated heat released per injection for the titration of CB7 by Busiprone at 298 K in sodium acetate buffer (pH 3.0). The data points represent the experimental values, while the fitted curve corresponds to the best-fit binding model used for thermodynamic analysis. 7
Fig. S3 Results of two selected isothermal titration calorimetry (ITC) experiments, showing the raw heat release data and the integrated heat released per injection for the titration of CB7 by Aripiprazole at 298 K in sodium acetate buffer (pH 3.0). The data points represent the experimental values, while the fitted curve corresponds to the best-fit binding model used for thermodynamic analysis. 8
Fig. S4 Results of two selected isothermal titration calorimetry (ITC) experiments, showing the raw heat release data and the integrated heat released per injection for the titration of CB7 by 1-Phenylpiperazine (**1a**) at 298 K in phosphate-citrate buffer (pH 4.0). The data points represent the experimental values, while the fitted curve corresponds to the best-fit binding model used for thermodynamic analysis. 9
Fig. S5 Results of two selected isothermal titration calorimetry (ITC) experiments, showing the raw heat release data and the integrated heat released per injection for the titration of CB7 by 1-(4-bromophenyl)piperazine (**1b**) at 298 K in phosphate-citrate buffer (pH 4.0). The data points represent the experimental values, while the fitted curve corresponds to the best-fit binding model used for thermodynamic analysis. 10
Fig. S6 Results of two selected isothermal titration calorimetry (ITC) experiments, showing the raw heat release data and the integrated heat released per injection for the titration of CB7 by 1-(4-iodophenyl)piperazine(**1c**) at 298 K in phosphate-citrate buffer (pH 4.0). The data points represent the experimental values, while the fitted curve corresponds to the best-fit binding model used for thermodynamic analysis. 11
Fig. S7 Results of two selected isothermal titration calorimetry (ITC) experiments, showing the raw heat release data and the integrated heat released per injection for the titration of CB7 by 1-(3-fluorophenyl)piperazine(**1d**) at 298 K in phosphate-citrate buffer (pH 4.0). The data points represent the experimental values, while the fitted curve corresponds to the best-fit binding model used for thermodynamic analysis. 12
Fig. S8 Results of two selected isothermal titration calorimetry (ITC) experiments, showing the raw heat release data and the integrated heat released per injection for the titration of CB7 by 1-(2,3-Dichlorophenyl)piperazine(**1e**) at 298 K in phosphate-citrate buffer (pH 4.0). The data points represent the experimental values, while the fitted curve corresponds to the best-fit binding model used for thermodynamic analysis. 13
Fig. S9 Results of two selected isothermal titration calorimetry (ITC) experiments, showing the raw heat release data and the integrated heat released per injection for the titration of CB7 by 1-(2,5-Dichlorophenyl)piperazine(**1f**) at 298 K in phosphate-citrate buffer (pH 4.0). The data points represent the experimental values, while the fitted curve corresponds to the best-fit binding model used for thermodynamic analysis. 14

Fig. S10 Results of two selected isothermal titration calorimetry (ITC) experiments, showing the raw heat release data and the integrated heat released per injection for the titration of CB7 by 1-(3,4-Dichlorophenyl)piperazine(**1g**) at 298 K in phosphate-citrate buffer (pH 4.0). The data points represent the experimental values, while the fitted curve corresponds to the best-fit binding model used for thermodynamic analysis. 15
Fig. S11 Results of two selected isothermal titration calorimetry (ITC) experiments, showing the raw heat release data and the integrated heat released per injection for the titration of CB7 by 1-(2,4-Difluorophenyl)piperazine(**1h**) at 298 K in phosphate-citrate buffer (pH 4.0). The data points represent the experimental values, while the fitted curve corresponds to the best-fit binding model used for thermodynamic analysis. 16
Fig. S12 Results of two selected isothermal titration calorimetry (ITC) experiments, showing the raw heat release data and the integrated heat released per injection for the titration of CB7 by 1-(2-Pyrimidyl)piperazine(**2a**) at 298 K in phosphate-citrate buffer (pH 4.0). The data points represent the experimental values, while the fitted curve corresponds to the best-fit binding model used for thermodynamic analysis. 17
Fig. S13 Results of two selected isothermal titration calorimetry (ITC) experiments, showing the raw heat release data and the integrated heat released per injection for the titration of CB7 by 5-bromo-2-(piperazin-1-yl)pyrimidine(**2b**) at 298 K in phosphate-citrate buffer (pH 4.0). The data points represent the experimental values, while the fitted curve corresponds to the best-fit binding model used for thermodynamic analysis. 18
Table S1 Summary of experiment conditions for Isothermal Titration Calorimetry (ITC)..........18
Table S2 Summary of crystallization conditions. 20
Table S3 Experimental details for XRD experiments. Experiments were carried out at 100 K. H-atom parameters were constrained...20
Fig. S14 Asymmetric unit of **1** shown in two orientations. (a,c) side view; (b,d) top view. (a,b) Refined model with anisotropic displacement parameters (ADPs) for non-H atoms (H atoms omitted for clarity). (c,d) Residual electron-density (difference-Fourier) maps for the same orientations, contoured at ±0.25 e Å^{-3} (green, +0.25 e Å^{-3}; red, −0.25 e Å^{-3}). 22
Fig. S15 Asymmetric unit of **2** shown in two orientations. (a,c) side view; (b,d) top view. (a,b) Refined model with anisotropic displacement parameters (ADPs) for non-H atoms (H atoms omitted for clarity). (c,d) Residual electron-density (difference-Fourier) maps for the same orientations, contoured at ±0.20 e Å^{-3} (green, +0.20 e Å^{-3}; red, −0.20 e Å^{-3}). 23
Fig. S16 Asymmetric unit of **3** shown in two orientations. (a,c) side view; (b,d) top view. (a,b) Refined model with anisotropic displacement parameters (ADPs) for non-H atoms (H atoms omitted for clarity). (c,d) Residual electron-density (difference-Fourier) maps for the same orientations, contoured at ±0.20 e Å^{-3} (green, +0.20 e Å^{-3}; red, −0.20 e Å^{-3}). 24
Fig. S17 Asymmetric unit of **4** shown in two orientations. (a,c) side view; (b,d) top view. (a,b) Refined model with anisotropic displacement parameters (ADPs) for non-H atoms (H atoms omitted for clarity). (c,d) Residual electron-density (difference-Fourier) maps for the same orientations, contoured at ±0.80 e Å^{-3} (green, +0.80 e Å^{-3}; red, −0.80 e Å^{-3}). 25
Table S4 Interaction energies calculated with different density functionals, basis sets and dielectric constants (DC) 26
Table S5 This table presents the various energy terms applicable to a given complex. The energy values for the observed disorder in the position of the ligand in the crystal structure are provided in Table 5. ΔEInt is the interaction energy, ΔEDisp is the SAPT dispersion energy, T is the temperature, ΔSAPR is the entropy obtained from APR method, ΔEDef is the deformation energy for the host and guest, ΔGExp is the experimental Gibbs free energy obtained from ITC

studies, ΔGCalcd is the Hostaš's Gibbs free energy. All the energy values are in [kj/mol] except T is in [K] and ΔS in [kJ/mol·K]. 26
Table S6 All SAPT energy components. All units are in [kJ/mol] 27
Table S7 Thermodynamic parameters for bonding reaction: ΔG is the Gibbs free energy [kJ / mol], ΔH is the enthalpy [kJ / mol], T is temperature of 298K, and ΔS is the entropy [kJ / mol·K] obtained using APR approach. 27
Table S8 All these parameters were obtained with molecular dynamics simulations by using APR method. ΔG is the Gibbs free energy [kJ / mol], ΔH is the enthalpy [kJ / mol], T is temperature [K] and ΔS is the entropy [kJ / mol·K]..27
Table S9 Geometrical parameters, QTAIM properties at the bond critical points (in a.u. x 10^3), $E(r_c)$ is in kJ/mol, and classification of all inter- and intramolecular hydrogen bonds. GC is geometric classification, EC corresponds to energy classification and HC stands for Hayashi classification. The number in square brackets corresponds to the glycolurill unit in CB7. 27
Fig. S18 Molecular graph for CB7·**1h** conformer 1. Dashed lines indicate a fluorine interaction. The small green dots indicate the bond critical points. White atoms are hydrogen, gray atoms are carbon, red atoms are oxygen, blue atoms are nitrogen, and green atoms are fluorine.................32
Fig. S19 Contour plot of the Laplacian of the electron density for CB7·**1h** conformer 1. The selected plane is formed between those atoms that belong to the intramolecular bond C2H···FC2'. Regions of charge-concentration and charge-depletion are respectively solid lines in red, $\nabla^2\rho(r_c) < 0$, and solid lines in blue, $\nabla^2\rho(r_c) > 0$. Bond paths between each atom pair of two nuclei painted in solid and dashed lines in atom color represent the shared- and closed-shell interactions, respectively, whereas the tiny spheres painted in green represent bond critical points. 33
Fig. S20 Contour plot of the Laplacian of the electron density for CB7·**1h** conformer 1. The selected plane is formed between those atoms that belong to the intramolecular bond [O1]3···FC2'. Regions of charge-concentration and charge-depletion are respectively solid lines in red, $\nabla^2\rho(r_c) < 0$, and solid lines in blue, $\nabla^2\rho(r_c) > 0$. Bond paths between each atom pair of two nuclei painted in solid and dashed lines in atom color represent the shared- and closed-shell interactions, respectively, whereas the tiny spheres painted in green represent bond critical points.33
Fig. S21 Contour plot of the Laplacian of the electron density for CB7·**1h** conformer 1. The selected plane is formed between those atoms that belong to the intramolecular bond [O1]4···FC2'. Regions of charge-concentration and charge-depletion are respectively solid lines in red, $\nabla^2\rho(r_c) < 0$, and solid lines in blue, $\nabla^2\rho(r_c) > 0$. Bond paths between each atom pair of two nuclei painted in solid and dashed lines in atom color represent the shared- and closed-shell interactions, respectively, whereas the tiny spheres painted in green represent bond critical points.34
Fig. S22 Relief maps of the Laplacian of the density for conformer 1 of CB7·**1h** conformer 1. The selected plane is formed between those atoms that belong to the intramolecular bond C2H···FC2'. The color ranges are: blue, more positive than 1; cyan, between 1 and 0.5; green, between −0.5 and 0.5; yellow, between −1 and −0.5; red, more negative than −1 au. 35
Fig. S23 Relief maps of the Laplacian of the density for conformer 1 of CB7·**1h** conformer 1. The selected plane is formed between those atoms that belong to the intramolecular bond [O1]3···FC2'. The color ranges are: blue, more positive than 1; cyan, between 1 and 0.5; green, between −0.5 and 0.5; yellow, between −1 and −0.5; red, more negative than −1 au. 35
Fig. S24 Relief maps of the Laplacian of the density for conformer 1 of CB7·**1h** conformer 1. The selected plane is formed between those atoms that belong to the intramolecular bond [O1]4···FC2'. The color ranges are: blue, more positive than 1; cyan, between 1 and 0.5; green, between −0.5 and 0.5; yellow, between −1 and −0.5; red, more negative than −1 au. 36

Table S10 Geometrical parameters, QTAIM properties at the bond critical points (in a.u. x 10^3), $E(r_c)$ is in kJ/mol, and classification of n → π* interactions. EC corresponds to energy classification and HC stands for Hayashi classification. The number in square brackets corresponds to the glycolurill unit in CB7· 36
Fig. S25 Experimental enthalpy energy (Table 2) versus interaction energy. The equation for the adjusted tendency (Continuous line) is y = 0.2295 x + 24.8810 and R^2=0.80..............................38
Fig. S26 Potential energy surfaces for the dihedral angle C2'-C1'-N1-C1 are shown for 1i in (●) and 1j in (●) with the structures at the minima overlaid...39

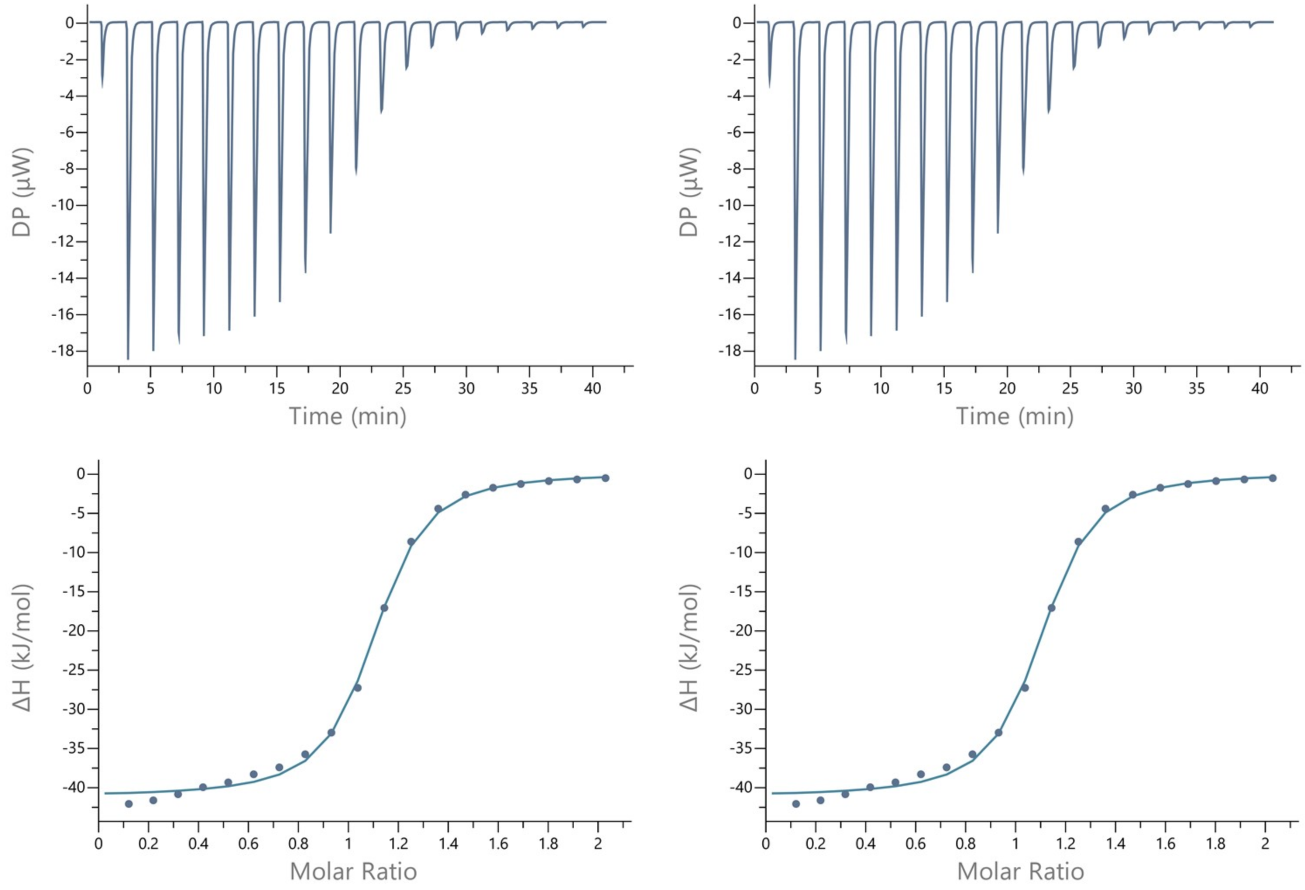

Fig. S1 Results of two selected isothermal titration calorimetry (ITC) experiments, showing the raw heat release data and the integrated heat released per injection for the titration of CB7 by Trazodone at 298 K in sodium acetate buffer (pH 3.0). The data points represent the experimental values, while the fitted curve corresponds to the best-fit binding model used for thermodynamic analysis.

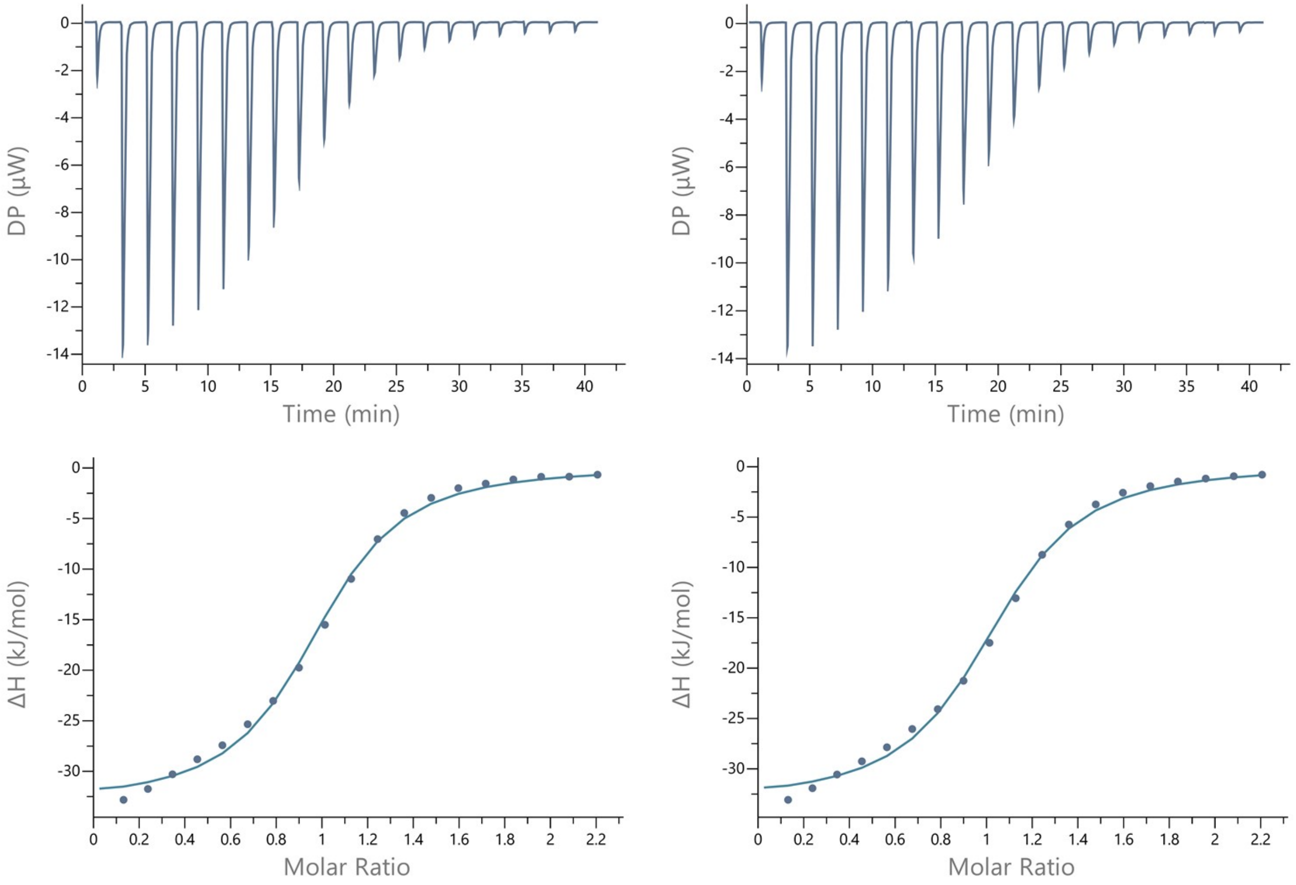

Fig. S2 Results of two selected isothermal titration calorimetry (ITC) experiments, showing the raw heat release data and the integrated heat released per injection for the titration of CB7 by Busiprone at 298 K in sodium acetate buffer (pH 3.0). The data points represent the experimental values, while the fitted curve corresponds to the best-fit binding model used for thermodynamic analysis.

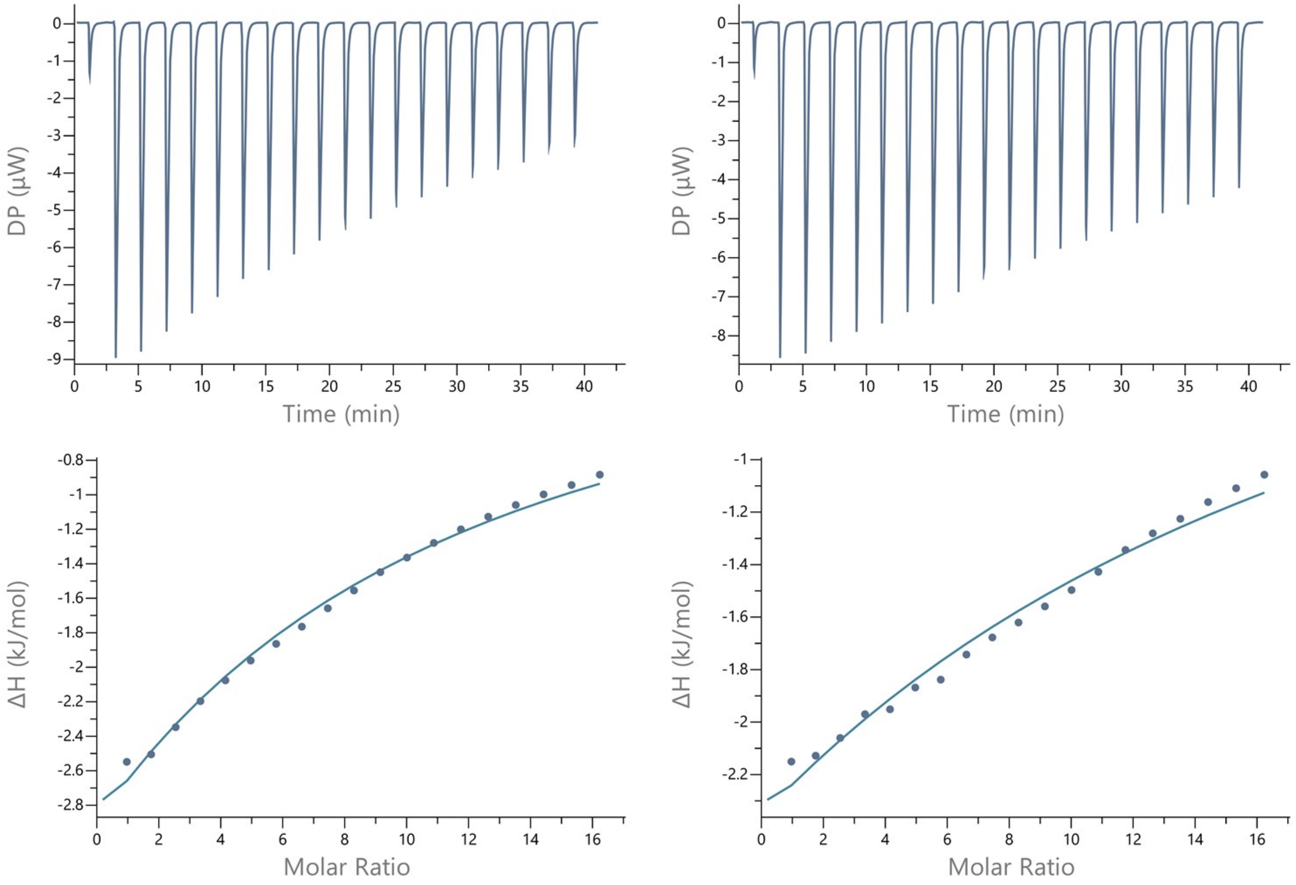

Fig. S3 Results of two selected isothermal titration calorimetry (ITC) experiments, showing the raw heat release data and the integrated heat released per injection for the titration of CB7 by Aripiprazole at 298 K in sodium acetate buffer (pH 3.0). The data points represent the experimental values, while the fitted curve corresponds to the best-fit binding model used for thermodynamic analysis.

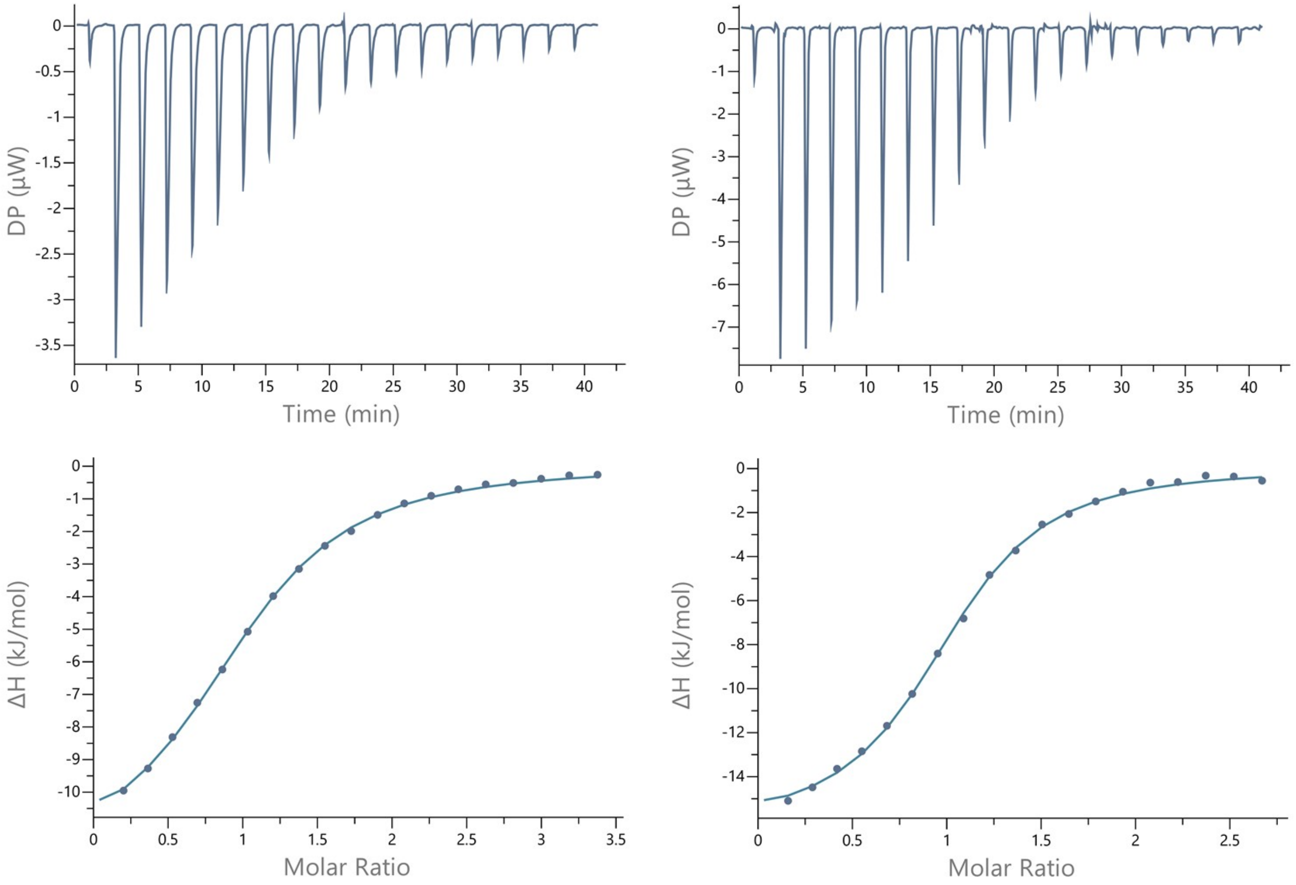

Fig. S4 Results of two selected isothermal titration calorimetry (ITC) experiments, showing the raw heat release data and the integrated heat released per injection for the titration of CB7 by 1-Phenylpiperazine (**1a**) at 298 K in phosphate-citrate buffer (pH 4.0). The data points represent the experimental values, while the fitted curve corresponds to the best-fit binding model used for thermodynamic analysis.

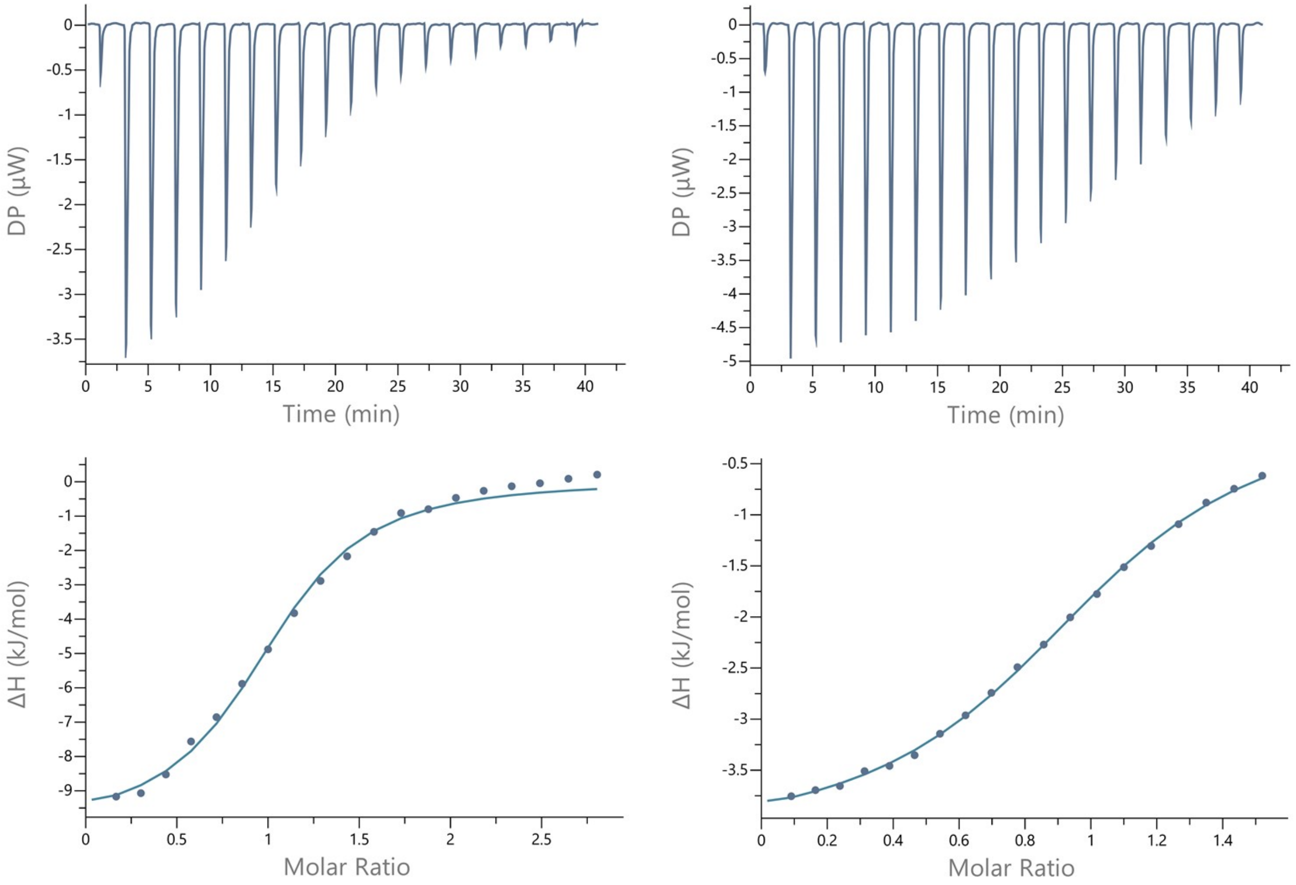

Fig. S5 Results of two selected isothermal titration calorimetry (ITC) experiments, showing the raw heat release data and the integrated heat released per injection for the titration of CB7 by 1-(4-bromophenyl)piperazine (**1b**) at 298 K in phosphate-citrate buffer (pH 4.0). The data points represent the experimental values, while the fitted curve corresponds to the best-fit binding model used for thermodynamic analysis.

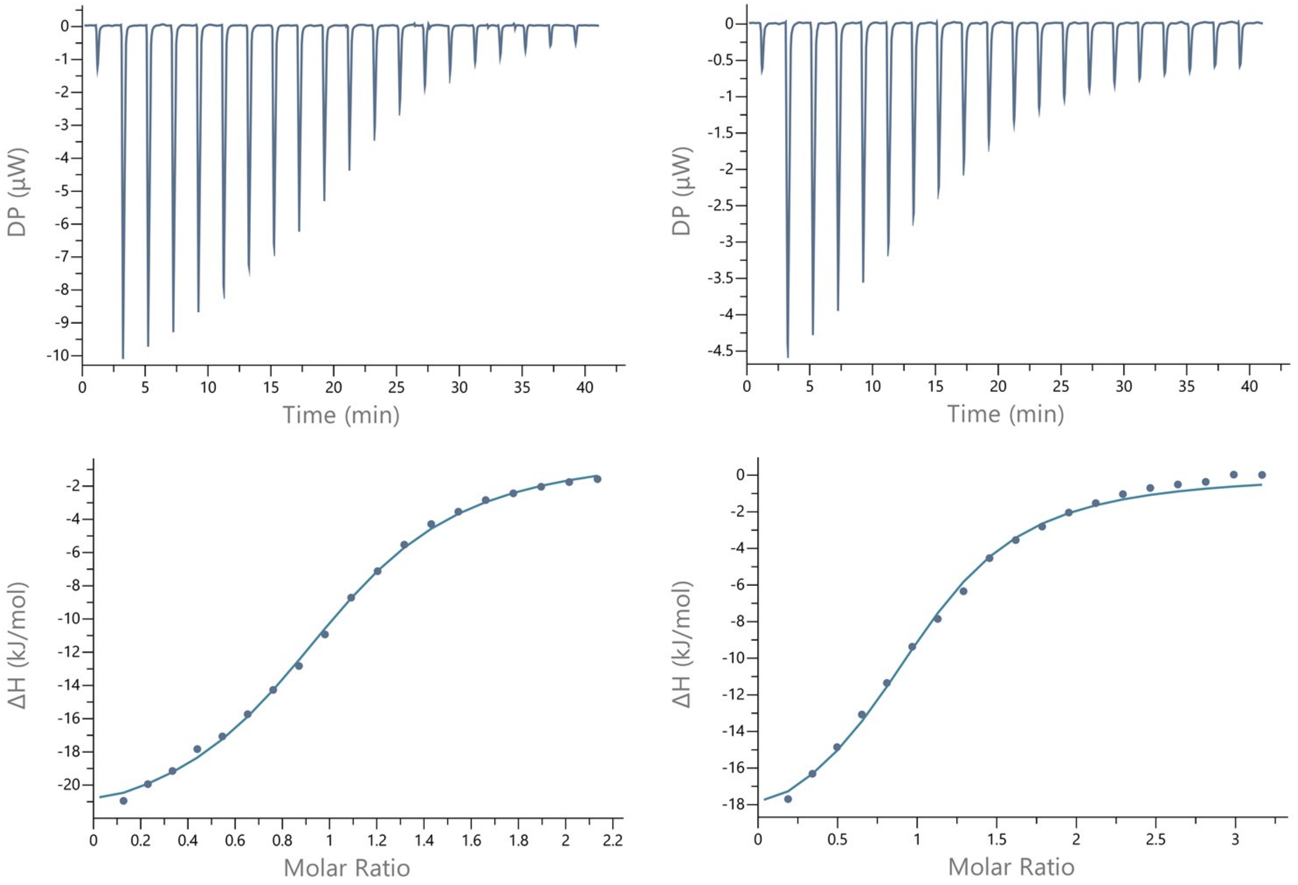

Fig. S6 Results of two selected isothermal titration calorimetry (ITC) experiments, showing the raw heat release data and the integrated heat released per injection for the titration of CB7 by 1-(4-iodophenyl)piperazine(**1c**) at 298 K in phosphate-citrate buffer (pH 4.0). The data points represent the experimental values, while the fitted curve corresponds to the best-fit binding model used for thermodynamic analysis.

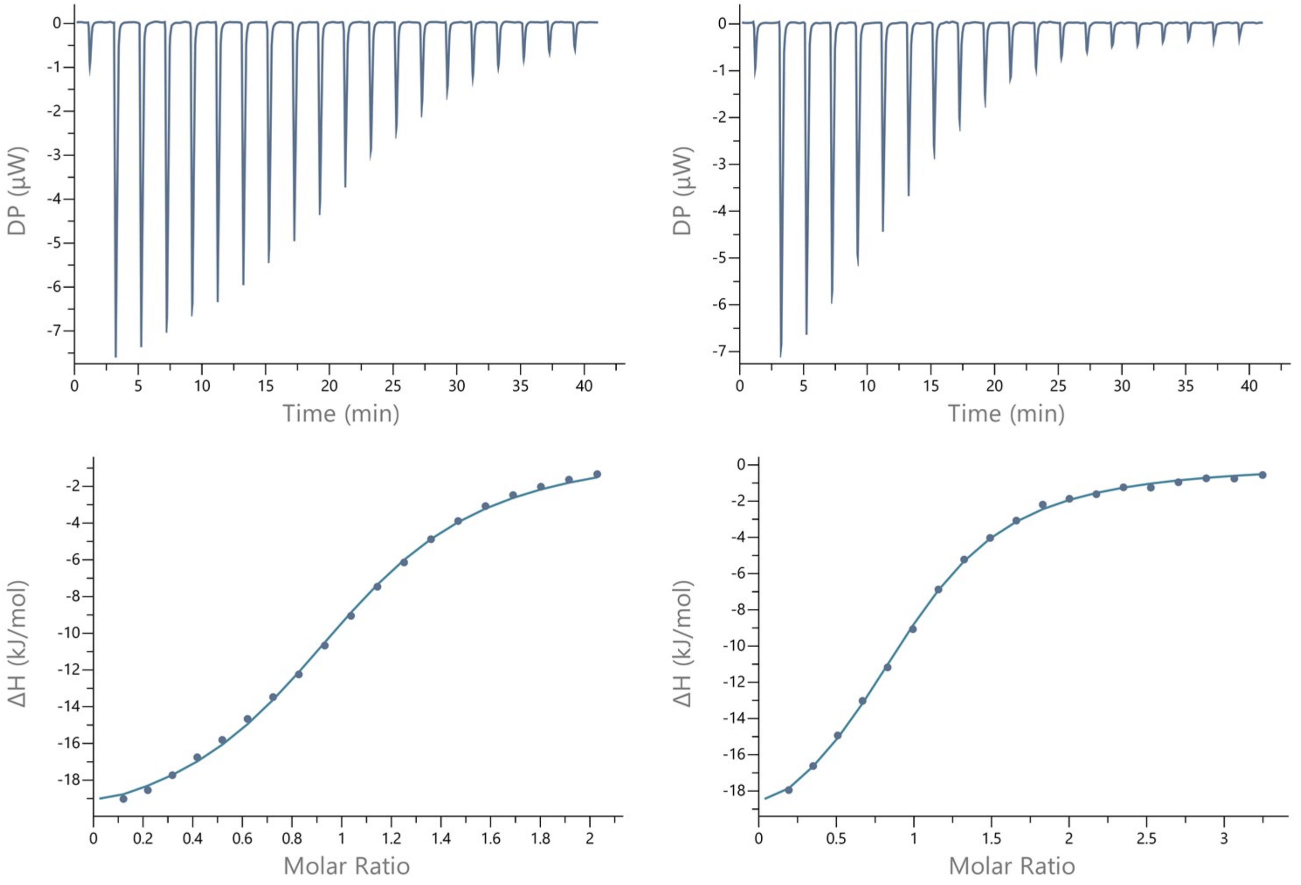

Fig. S7 Results of two selected isothermal titration calorimetry (ITC) experiments, showing the raw heat release data and the integrated heat released per injection for the titration of CB7 by 1-(3-fluorophenyl)piperazine(**1d**) at 298 K in phosphate-citrate buffer (pH 4.0). The data points represent the experimental values, while the fitted curve corresponds to the best-fit binding model used for thermodynamic analysis.

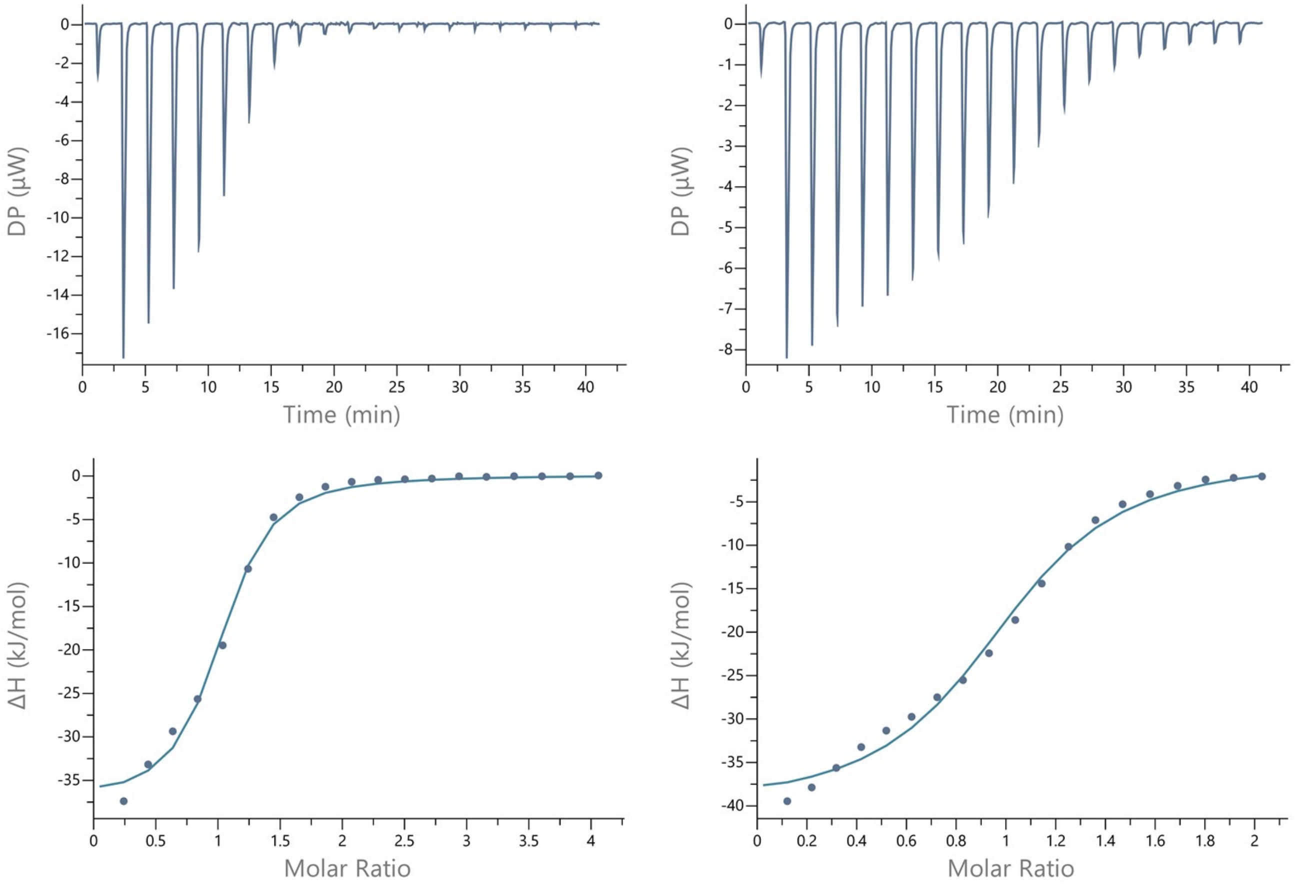

Fig. S8 Results of two selected isothermal titration calorimetry (ITC) experiments, showing the raw heat release data and the integrated heat released per injection for the titration of CB7 by 1-(2,3-Dichlorophenyl)piperazine(**1e**) at 298 K in phosphate-citrate buffer (pH 4.0). The data points represent the experimental values, while the fitted curve corresponds to the best-fit binding model used for thermodynamic analysis.

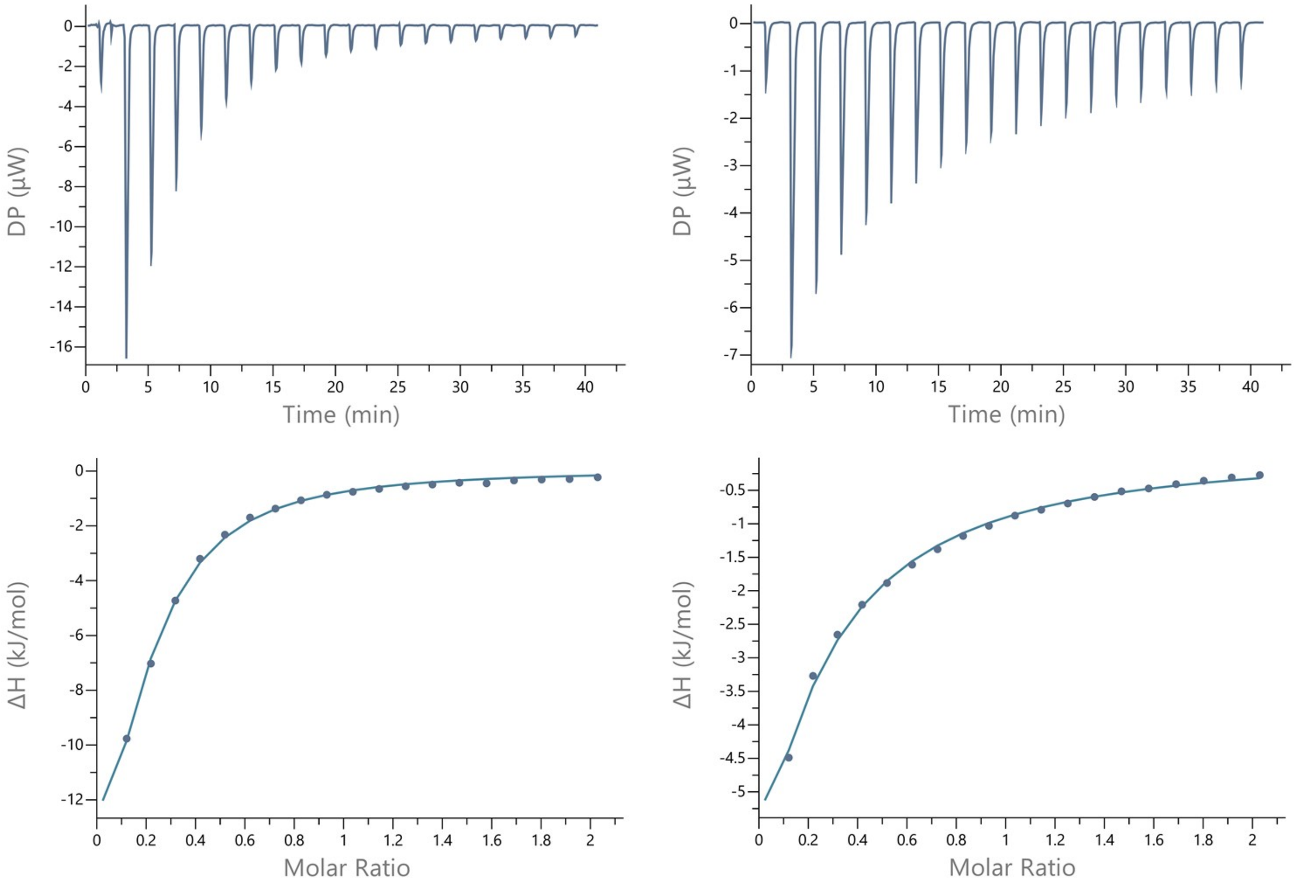

Fig. S9 Results of two selected isothermal titration calorimetry (ITC) experiments, showing the raw heat release data and the integrated heat released per injection for the titration of CB7 by 1-(2,5-Dichlorophenyl)piperazine(**1f**) at 298 K in phosphate-citrate buffer (pH 4.0). The data points represent the experimental values, while the fitted curve corresponds to the best-fit binding model used for thermodynamic analysis.

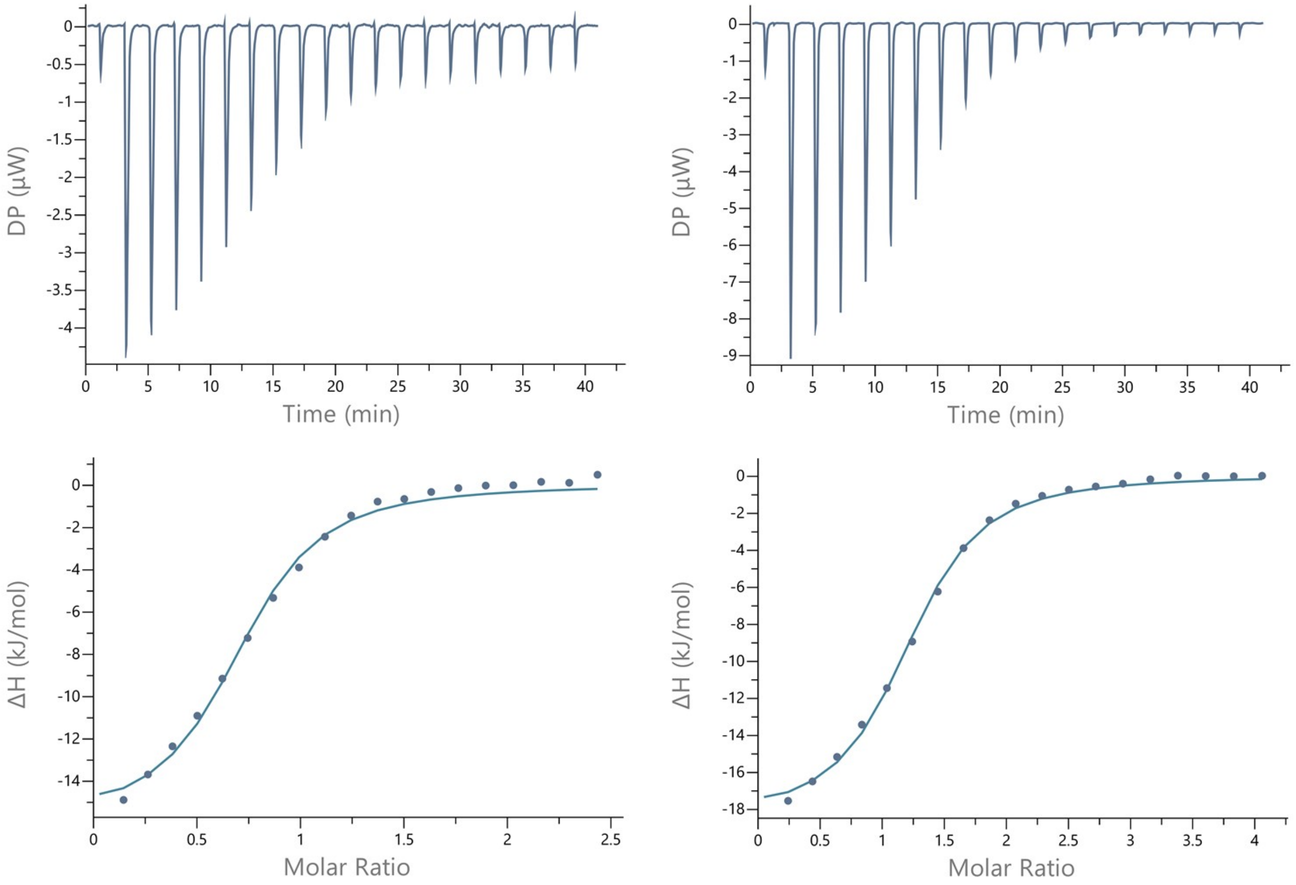

Fig. S10 Results of two selected isothermal titration calorimetry (ITC) experiments, showing the raw heat release data and the integrated heat released per injection for the titration of CB7 by 1-(3,4-Dichlorophenyl)piperazine(**1g**) at 298 K in phosphate-citrate buffer (pH 4.0). The data points represent the experimental values, while the fitted curve corresponds to the best-fit binding model used for thermodynamic analysis.

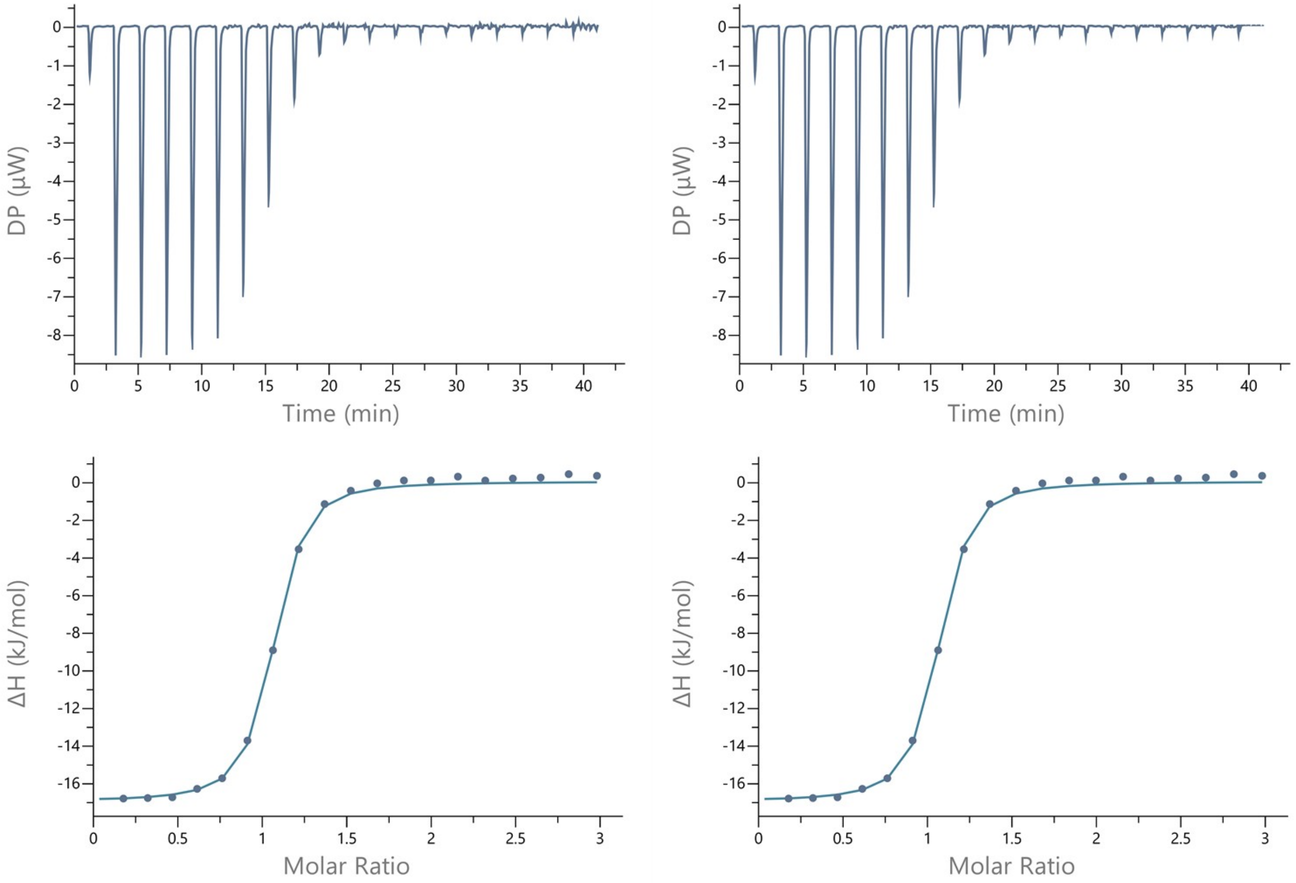

Fig. S11 Results of two selected isothermal titration calorimetry (ITC) experiments, showing the raw heat release data and the integrated heat released per injection for the titration of CB7 by 1-(2,4-Difluorophenyl)piperazine(**1h**) at 298 K in phosphate-citrate buffer (pH 4.0). The data points represent the experimental values, while the fitted curve corresponds to the best-fit binding model used for thermodynamic analysis.

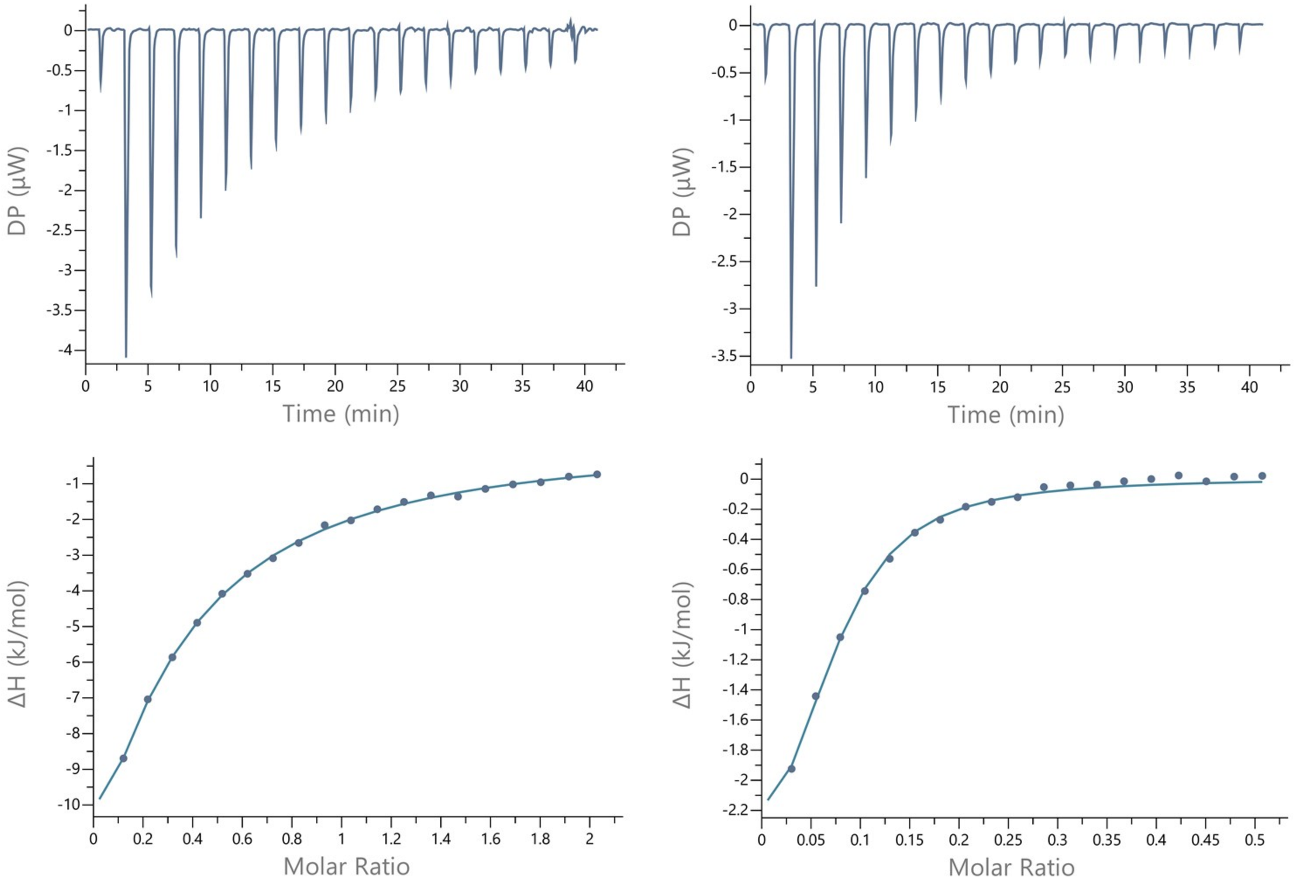

Fig. S12 Results of two selected isothermal titration calorimetry (ITC) experiments, showing the raw heat release data and the integrated heat released per injection for the titration of CB7 by 1-(2-Pyrimidyl)piperazine(**2a**) at 298 K in phosphate-citrate buffer (pH 4.0). The data points represent the experimental values, while the fitted curve corresponds to the best-fit binding model used for thermodynamic analysis.

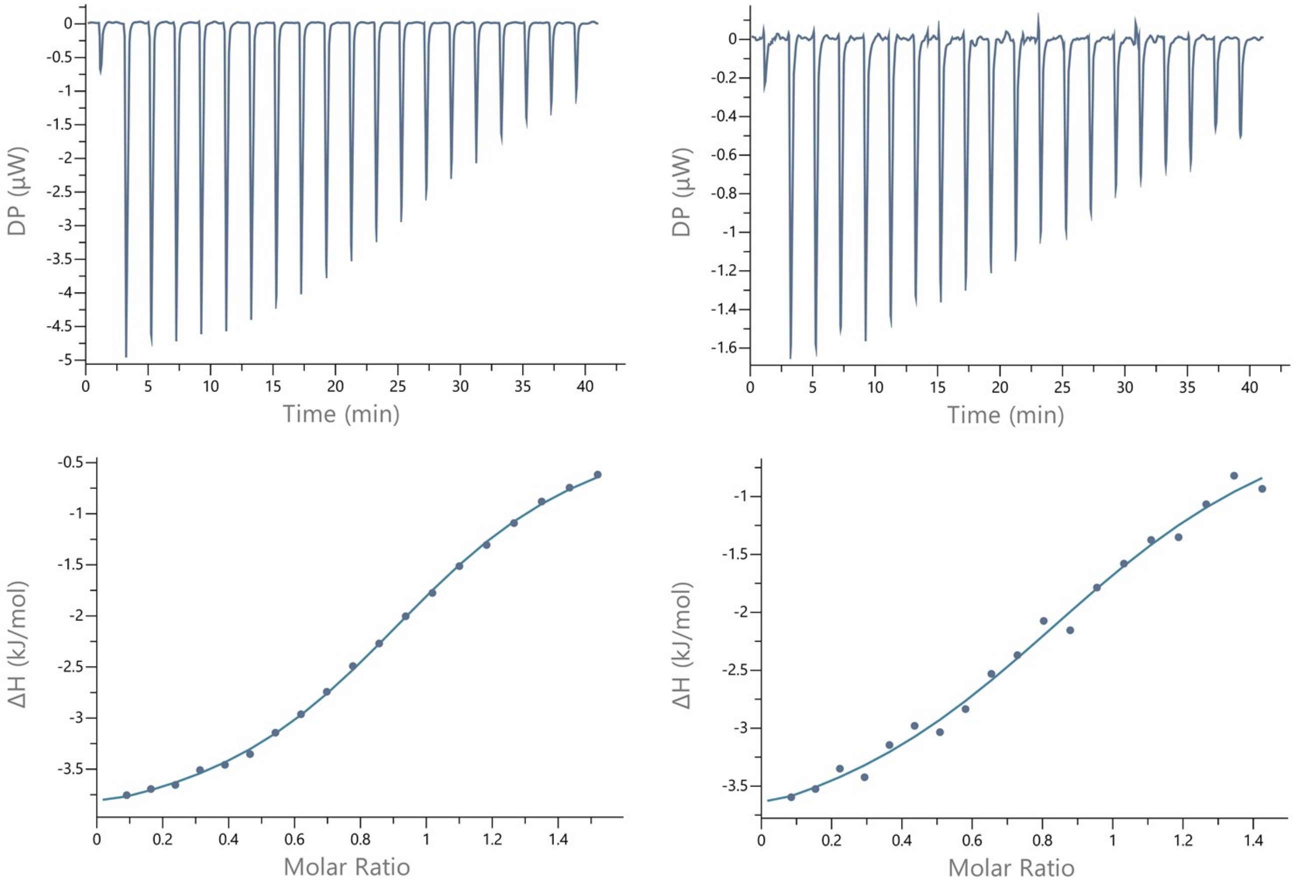

Fig. S13 Results of two selected isothermal titration calorimetry (ITC) experiments, showing the raw heat release data and the integrated heat released per injection for the titration of CB7 by 5-bromo-2-(piperazin-1-yl)pyrimidine(**2b**) at 298 K in phosphate-citrate buffer (pH 4.0). The data points represent the experimental values, while the fitted curve corresponds to the best-fit binding model used for thermodynamic analysis.

Table S1 Summary of experiment conditions for Isothermal Titration Calorimetry (ITC).

**Entry**	**Cucurbit [7] uril (mM)**	**Phenylpiperazine derivatives**	**Phenylpiperazine derivatives (mM)**	**Buffer**	**pH**	**Temperature (°C)**
1.	0.25	**1a**	2.5	Phosphate-Citrate	4.0	25
2.	0.25<br>1.0<br>2.0	**2a**	2.5<br>5.0<br>10.0	Phosphate-Citrate	4.0	25

Entry	Cucurbit [7] uril (mM)	Phenylpiperazine derivatives	Phenylpiperazine derivatives (mM)	Buffer	pH	Temperature (^{o}C)
	4.0		10.0			
	4.0		15.0			
	4.0		30.0			
	0.5		2.0			
3.	0.5	**1b**	2.5	Phosphate-Citrate	4.0	25
	1.5		2.5			
	0.25		2.5			
4.	0.5	**1c**	1.25	Phosphate-Citrate	4.0	25
	1.5		2.5			
	0.25		1.25			
5.	0.25	**1e**	2.5	Phosphate-Citrate	4.0	25
	0.5		2.5			
	0.25		1.5			
6.	0.25	**1g**	2.5	Phosphate-Citrate	4.0	25
	0.5		2.5			
	0.25		2.5			
7.	1.0	**1f**	2.0	Phosphate-Citrate	4.0	25
	2.0		10.0			
	0.25		2.5			
8.	0.5	**1d**	2.0	Phosphate-Citrate		25
	1.0		2.0			
9.	0.25	**1h**	2.5	Phosphate-Citrate	4.0	25
10.	0.25	**1i**	2.5	Phosphate-Citrate	4.0	25
12.	0.5	**2b**	5.0	Phosphate-Citrate	4.0	25
	0.5		7.5			

Table S2 Summary of crystallization conditions.

Entry	Host	Ligand	Ligand / Host ratio	Solvent	pH	Temperature (^{0}C)
1.	CB[7]	1-Phenylpiperazine (**1a**)	1:4	H_2SO_4	2	25
2.	CB[7]	1-(2,4-difluorophenyl)piperazine (**1f**)	1:4	H_2SO_4	4	25
3.	CB[7]	1-(4-chloro-2-fluorophenyl)piperazine (**1i**)	1:4	H_2SO_4	2	25
4.	CB[7]	1-(2-Pyrimidyl)piperazine (**2a**)	1:4	H_2SO_4	4	25
^{a} Reaction conditions: Cucurbit [7] uril (CB[7], 0.007 mmol, 1 eqiv.).						

Table S3 Experimental details for XRD experiments. Experiments were carried out at 100 K. H-atom parameters were constrained.

	(1)	(2)	(3)	(4)
Crystal data				
Chemical formula	$C_{42}H_{42}N_{28}O_{14}\cdot 2(H_2O)\cdot C_{10}H_{15}N_2\cdot O\cdot 19[H2O]$	$2(Cl)\cdot C_{42}H_{42}N_{28}O_{14}\cdot C_{10}H_{13}F_2N_2\cdot 16[H2O]$	$C_{10}H_{13}ClFN_2\cdot 1.5(C_{42}H_{42}N_{28}O_{14})\cdot 53[H2O]$	$C_{42}H_{42}N_{28}Na_{3.5}O_{27}\cdot C_8H_{13}N_4\cdot 8[H2O]$
M_r	1720.61	1721.41	2915.07	1760.85
Crystal system, space group	Tetragonal, *I*4	Tetragonal, *I*4	Orthorhombic, $C222_1$	Orthorhombic, *Pnn*2
a, *b*, *c* (Å)	28.306 (4), 28.306 (4), 18.617 (4)	28.6126 (1), 28.6126 (1), 18.3038 (2)	32.4607 (6), 39.2830 (8), 20.2199 (3)	28.9179 (5), 26.6083 (4), 10.6290 (1)
α, β, γ (°)	90, 90, 90	90, 90, 90	90, 90, 90	90, 90, 90
V (Å^3)	14916 (5)	14984.97 (19)	25783.5 (8)	8178.5 (2)
Z	8	8	8	4
Radiation type	Synchrotron, λ = 0.500 Å	Cu *K*α	Cu *K*α	Cu *K*α
m (mm^{-1})	0.06	1.74	1.35	1.21
Crystal size (mm)	0.05 × 0.02 × 0.02	0.23 × 0.17 × 0.16	0.4 × 0.25 × 0.23	0.81 × 0.14 × 0.05
Data collection				
Diffractometer	Synchrotron	SuperNova, Dual, Cu at home/near, Atlas	SuperNova, Dual, Cu at home/near, Atlas	SuperNova, Dual, Cu at home/near, HyPix
Absorption correction	–	Multi-scan *CrysAlis PRO* 1.171.40.84a (Rigaku Oxford Diffraction, 2020) Empirical absorption	Multi-scan *CrysAlis PRO* 1.171.39.46 (Rigaku Oxford Diffraction, 2018) Empirical absorption	Gaussian *CrysAlis PRO* 1.171.42.36a (Rigaku Oxford Diffraction, 2021) Numerical

		correction using spherical harmonics, implemented in SCALE3 ABSPACK scaling algorithm.	correction using spherical harmonics, implemented in SCALE3 ABSPACK scaling algorithm.	absorption correction based on gaussian integration over a multifaceted crystal model Empirical absorption correction using spherical harmonics, implemented in SCALE3 ABSPACK scaling algorithm.
T_{min}, T_{max}	–	0.840, 1.000	0.802, 1.000	0.583, 1.000
No. of measured, independent and observed [$I > 2\sigma(I)$] reflections	262240, 17128, 13710	142724, 14038, 13014	88456, 20353, 17074	40141, 12470, 7865
R_{int}	0.056	0.026	0.075	0.058
$(\sin \theta/\lambda)_{max}$ (Å^{-1})	0.649	0.619	0.576	0.602
Refinement				
$R[F^2 > 2\sigma(F^2)]$, $wR(F^2)$, S	0.067, 0.223, 1.09	0.051, 0.152, 1.07	0.065, 0.199, 1.05	0.130, 0.385, 1.40
No. of reflections	17128	14038	20353	12470
No. of parameters	938	916	1283	671
No. of restraints	140	69	173	368
	$w = 1/[\sigma^2(F_o^2) + (0.151P)^2 + 2.6259P]$ where $P = (F_o^2 + 2F_c^2)/3$	$w = 1/[\sigma^2(F_o^2) + (0.0866P)^2 + 12.5155P]$ where $P = (F_o^2 + 2F_c^2)/3$	$w = 1/[\sigma^2(F_o^2) + (0.1403P)^2]$ where $P = (F_o^2 + 2F_c^2)/3$	$w = 1/[\sigma^2(F_o^2) + (0.2P)^2]$ where $P = (F_o^2 + 2F_c^2)/3$
$\Delta\rho_{max}$, $\Delta\rho_{min}$ (e Å^{-3})	0.61, -0.77	0.66, -0.31	0.34, -0.23	1.96, -1.63
Absolute structure	Flack x determined using 5786 quotients [(I+)-(I-	Flack x determined using 5536 quotients [(I+)-(I-	Flack x determined using 6551 quotients [(I+)-(I-	Flack x determined using 2096 quotients

	)]/[(I+)+(I-)] (Parsons, Flack and Wagner, Acta Cryst. B69 (2013) 249-259).	)]/[(I+)+(I-)] (Parsons, Flack and Wagner, Acta Cryst. B69 (2013) 249-259).	)]/[(I+)+(I-)] (Parsons, Flack and Wagner, Acta Cryst. B69 (2013) 249-259).	[(I+)-(I-)]/[(I+)+(I-)] (Parsons, Flack and Wagner, Acta Cryst. B69 (2013) 249-259).
Absolute structure paramete r	0.5 (5)	0.502 (6)	0.12 (3)	0.49 (13)

Computer programs: *CrysAlis PRO* 1.171.40.84a (Rigaku OD, 2020), *CrysAlis PRO* 1.171.39.46 (Rigaku OD, 2018), *CrysAlis PRO* 1.171.42.36a (Rigaku OD, 2021), *SHELXT* 2014/5 (Sheldrick, 2014), *SHELXD* (Sheldrick, 2008), *SHELXT* 2018/2 (Sheldrick, 2018), *SHELXL* 2019/2 (Sheldrick, 2015), Olex2 1.5 (Dolomanov *et al.*, 2009).

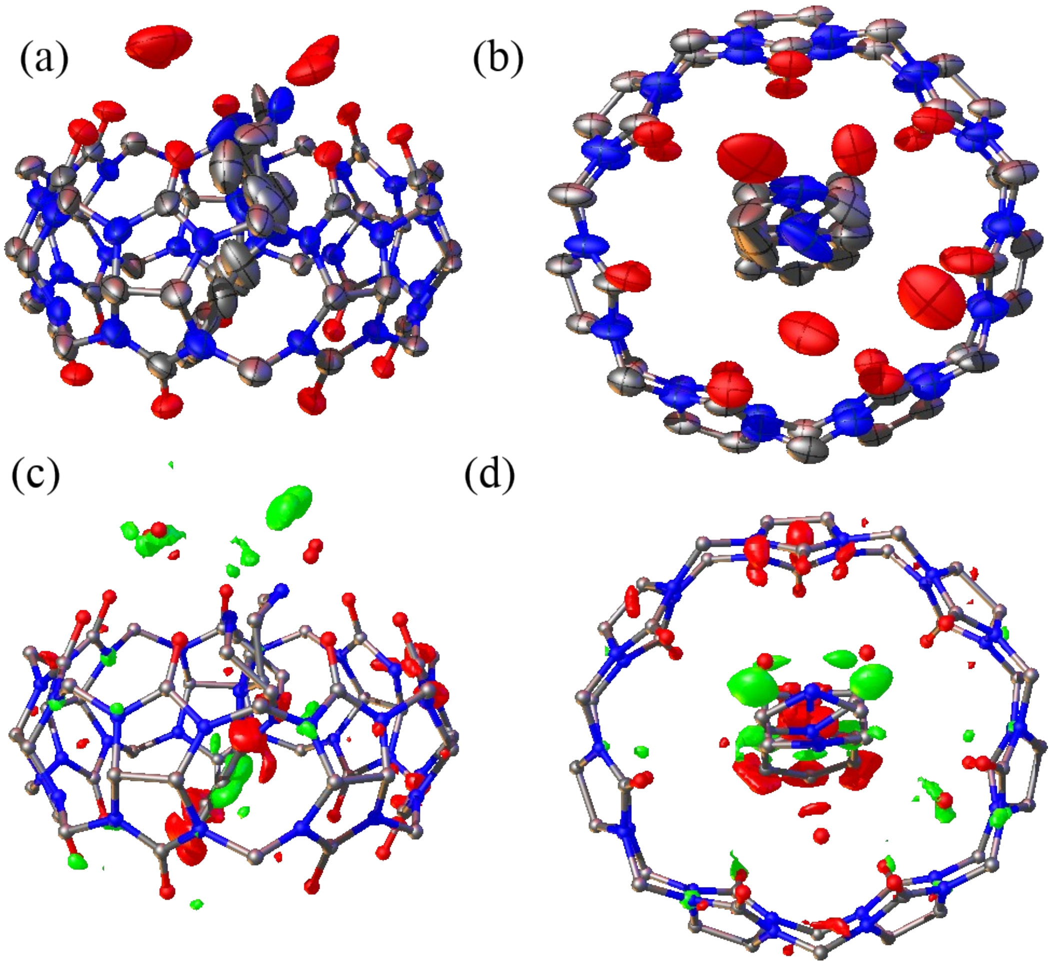

Fig. S14 Asymmetric unit of **1** shown in two orientations. (a,c) side view; (b,d) top view. (a,b) Refined model with anisotropic displacement parameters (ADPs) for non-H atoms (H atoms omitted for clarity). (c,d) Residual electron-density (difference-Fourier) maps for the same orientations, contoured at ±0.25 e Å^{-3} (green, +0.25 e Å^{-3}; red, −0.25 e Å^{-3}).

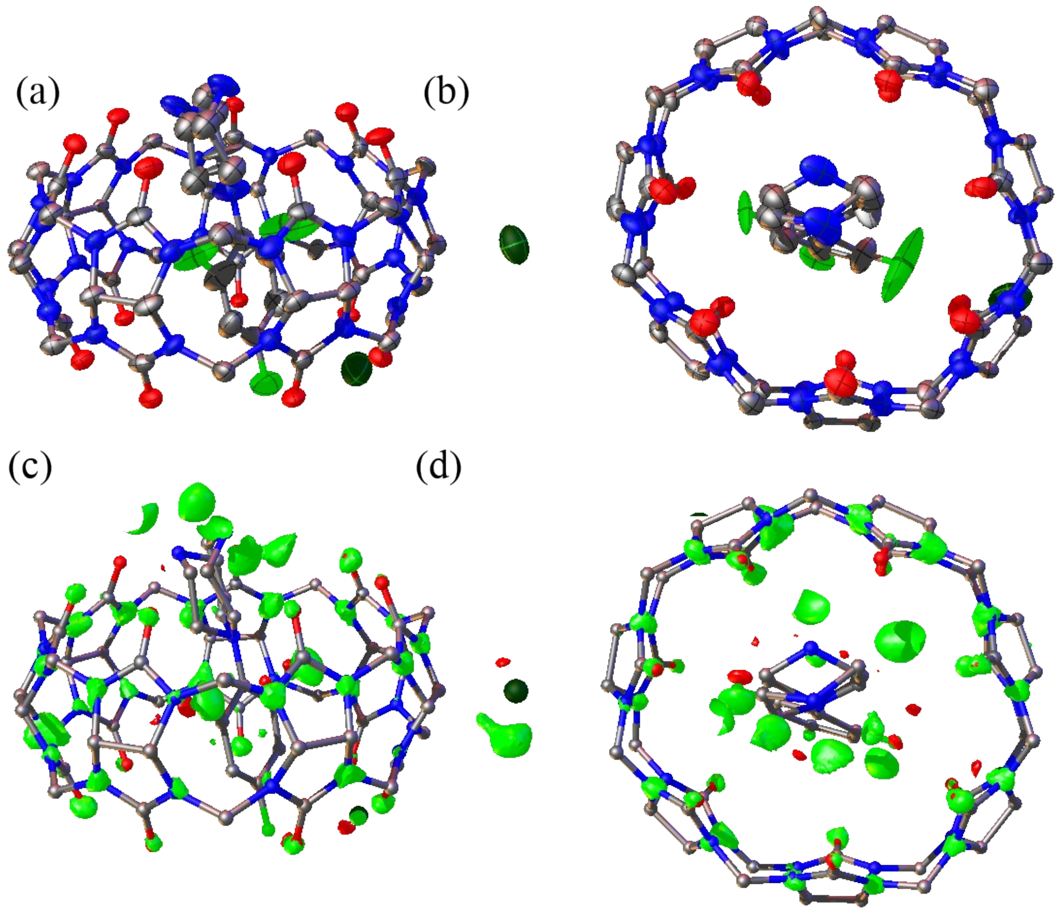

Fig. S15 Asymmetric unit of **2** shown in two orientations. (a,c) side view; (b,d) top view. (a,b) Refined model with anisotropic displacement parameters (ADPs) for non-H atoms (H atoms omitted for clarity). (c,d) Residual electron-density (difference-Fourier) maps for the same orientations, contoured at ±0.20 e $Å^{-3}$ (green, +0.20 e $Å^{-3}$; red, −0.20 e $Å^{-3}$).

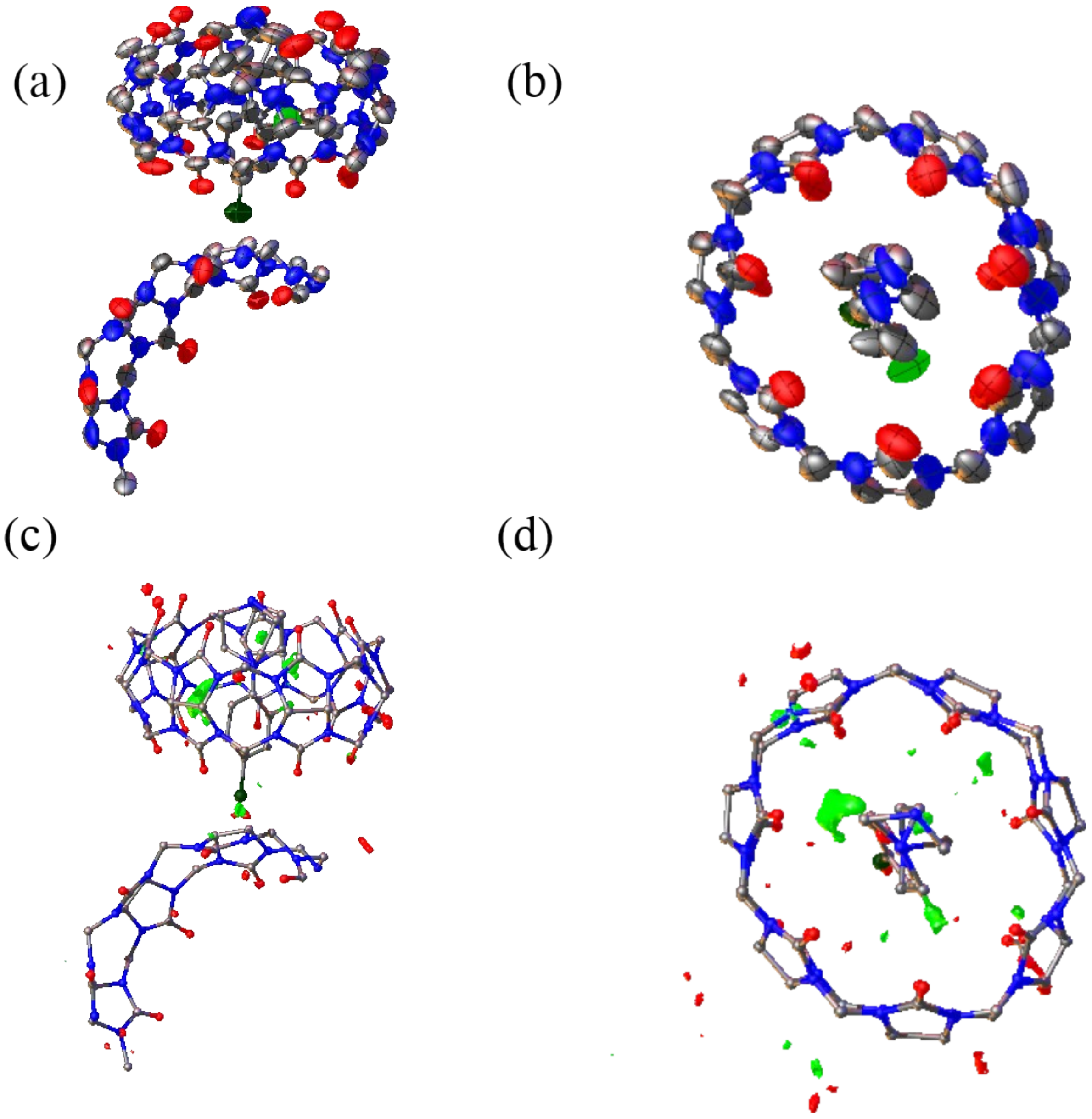

Fig. S16 Asymmetric unit of **3** shown in two orientations. (a,c) side view; (b,d) top view. (a,b) Refined model with anisotropic displacement parameters (ADPs) for non-H atoms (H atoms omitted for clarity). (c,d) Residual electron-density (difference-Fourier) maps for the same orientations, contoured at ±0.20 e Å^{-3} (green, +0.20 e Å^{-3}; red, −0.20 e Å^{-3}).

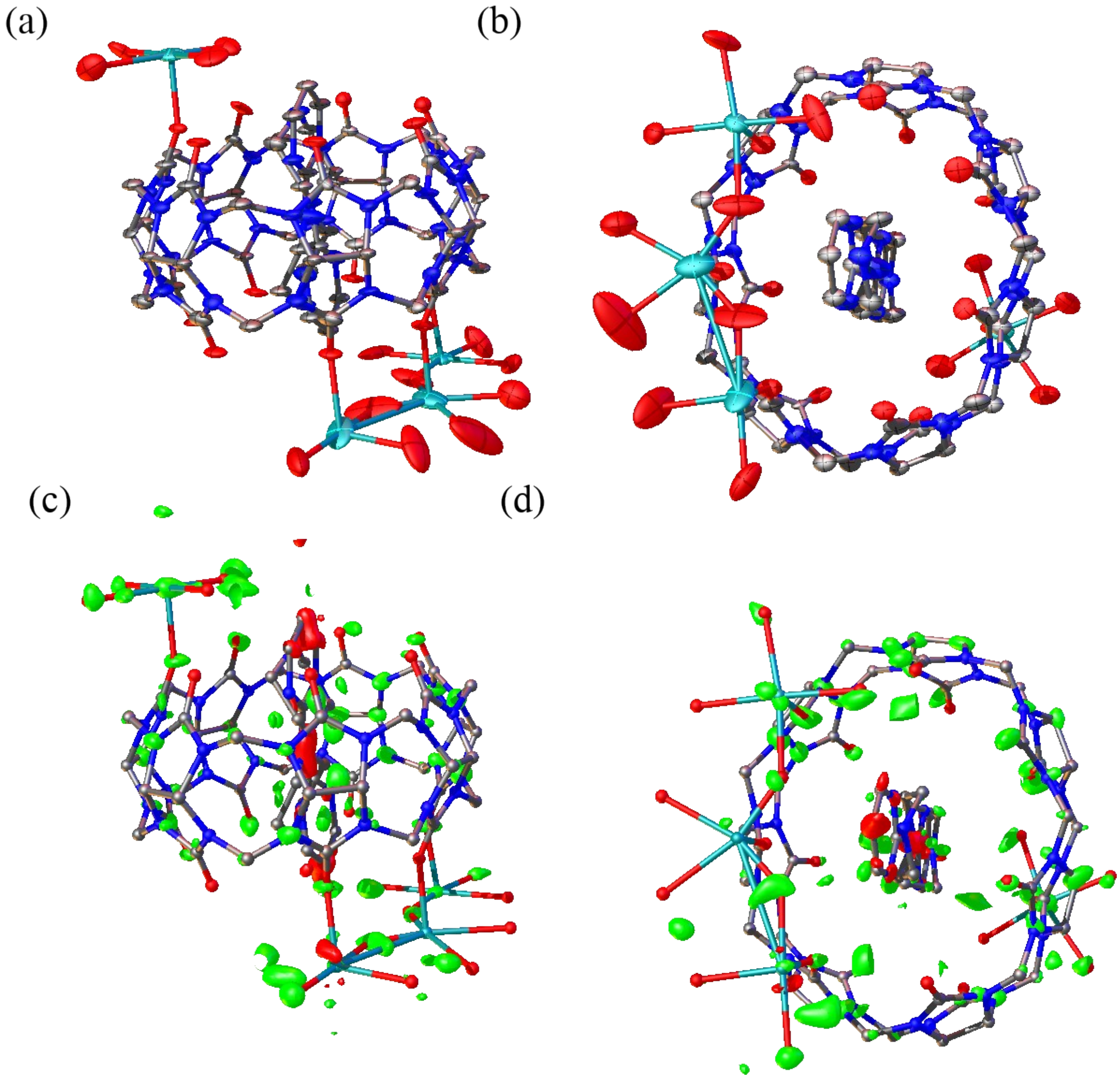

Fig. S17 Asymmetric unit of **4** shown in two orientations. (a,c) side view; (b,d) top view. (a,b) Refined model with anisotropic displacement parameters (ADPs) for non-H atoms (H atoms omitted for clarity). (c,d) Residual electron-density (difference-Fourier) maps for the same orientations, contoured at ±0.80 e Å^{-3} (green, +0.80 e Å^{-3}; red, −0.80 e Å^{-3}).

Table S4 Interaction energies calculated with different density functionals, basis sets and dielectric constants (DC)

	**Functional**	**M062X**	**TPSS0**	**TPSS0**	**WB97M-V**
	**Basis set**	**cc-pvDZ**	**cc-pvTZ**	**cc-pvTZ**	**cc-pvTZ**
**Complex**	**DC** / **Conformer**	**Water**	**Gas**	**Water**	**Water**

	Functional	M062X	TPSS0	TPSS0	WB97M-V
	Basis set	cc-pvDZ	cc-pvTZ	cc-pvTZ	cc-pvTZ
Complex	DC / Conformer	Water	Gas	Water	Water
CB[7]·**1a**	1	-169.0	-228.3	-24.0	-195.8
CB[7]·**1a**	2	-158.2	-270.7	-28.4	-187.2
CB[7]·**1b**	-	-155.2	-268.1	-25.9	-187.3
CB[7]·**1h**	1	-176.5	-274.2	-34.2	-205.2
CB[7]·**1h**	2	-177.9	-277.6	-33.4	-206.3
CB[7]·**1i**	1	-178.8	-278.2	-34.2	-209.3
CB[7]·**1i**	2	-168.4	-271.3	-29.2	-198.0
CB[7]·**2a**	1	-153.1	-283.2	-30.7	-181.0
CB[7]·**2a**	2	-163.2	-281.3	-35.8	-190.6

Table S5 This table presents the various energy terms applicable to a given complex. The energy values for the observed disorder in the position of the ligand in the crystal structure are provided in Table 5. ΔE_{Int} is the interaction energy, ΔE_{Disp} is the SAPT dispersion energy, T is the temperature, ΔS_{APR} is the entropy obtained from APR method, ΔE_{Def} is the deformation energy for the host and guest, ΔG_{Exp} is the experimental Gibbs free energy obtained from ITC studies, ΔG_{Calcd} is the Hostaš's Gibbs free energy. All the energy values are in [kj/mol] except T is in [K] and ΔS in [kJ/mol·K].

Complex	ΔEInt	ΔEDisp	ΔGSolv	-T·ΔSAPR	ΔEDef(Host)	ΔEDef(Guest)	ΔGExp	ΔGCalcd
CB[7]·**1a**	-169.0	-180.5	268.6	-64.1	130.1	10.1	-27.5	-65.7
CB[7]·**1b**	-155.2	-174.6	281.3	-18.2	137.0	14.7	-23.2	-27.9
CB[7]·**1h**	-179.0	-178.2	262.5	-49.1	144.0	17.1	-33.6	-73.7
CB[7]·**1i**	-178.8	-182.1	265.4	-71.6	136.7	14.6	-32.9	-104.3
CB[7]·**2a**	-163.2	-158.3	267.7	-5.8	145.5	20.0	-21.4	-21.9

Table S6 All SAPT energy components. All units are in [kJ/mol]

Complex	Conformer	$\Delta E_{Electrostatics}$	$\Delta E_{Exchange}$	$\Delta E_{Induction}$	$\Delta E_{Dispersion}$	ΔE_{Total}
CB[7]·**1a**	1	-256.10	126.61	-70.05	-180.43	-379.97
CB[7]·**1a**	2	-304.30	124.91	-72.56	-169.57	-421.51
CB[7]·**1b**	-	-298.23	133.77	-80.82	-174.59	-419.87
CB[7]·**1h**	1	-304.26	118.87	-69.20	-173.89	-428.49
CB[7]·**1h**	2	-310.72	126.53	-72.46	-177.76	-434.41
CB[7]·**1i**	1	-310.05	127.49	-74.64	-181.31	-438.52
CB[7]·**1i**	2	-306.79	135.05	-76.92	-178.68	-427.35
CB[7]·**1j**	-	-300.89	122.84	-64.96	-201.59	-444.60
CB[7]·**2a**	1	-314.10	104.87	-62.38	-154.42	-426.03
CB[7]·**2a**	2	-307.64	102.91	-63.18	-157.67	-425.58

Table S7 Thermodynamic parameters for bonding reaction: ΔG is the Gibbs free energy [kJ / mol], ΔH is the enthalpy [kJ / mol], T is temperature of 298K, and ΔS is the entropy [kJ / mol·K] obtained using APR approach.

Complex	Conformer	ΔG	ΔH	-T·ΔS
CB[7]·**1a**	1	-180.2	-220.8	40.6
CB[7]·**1a**	2	-290.2	-225.8	-64.4
CB[7]·**1b**	-	-240.0	-221.8	-18.2
CB[7]·**1h**	1	-323.7	-271.3	-52.4
CB[7]·**1h**	2	-313.8	-273.9	-39.9
CB[7]·**1i**	1	-347.0	-272.6	-74.4
CB[7]·**1i**	2	-328.1	-274.4	-53.7
CB[7]·**2a**	1	-220.4	-201.5	-18.9
CB[7]·**2a**	2	-215.5	-214.9	-0.6

Table S8 All these parameters were obtained with molecular dynamics simulations by using APR method. ΔG is the Gibbs free energy [kJ / mol], ΔH is the enthalpy [kJ / mol], T is temperature [K] and ΔS is the entropy [kJ / mol·K].

Complex	ΔG_{APR}	ΔH_{APR}	$-T\cdot\Delta S_{APR}$
CB[7]·**1a**	-288.9	-224.7	-64.2
CB[7]·**1b**	-240.0	-221.8	-18.2
CB[7]·**1h**	-322.5	-272.7	-49.8
CB[7]·**1i**	-345.7	-273.5	-72.3
CB[7]·**2a**	-219.9	-211.0	-9.0

Table S9 Geometrical parameters, QTAIM properties at the bond critical points (in a.u. x 10^3), $E(r_c)$ is in kJ/mol, and classification of all inter- and intramolecular hydrogen bonds. GC is geometric classification, EC corresponds to energy classification and HC stands for Hayashi classification. The number in square brackets corresponds to the glycolurill unit in CB7.

Complex	Conformer	Bond	∠(X–H···X)	d(Å)	$\rho(r_c)$	$\nabla^2\rho(r_c)$	$V(r_c)$	$H(r_c)$	$E(r_c)$	GC	EC	HC
CB7·1a	1	[O1]3···HC3'	144.9	2.434	9.3	35.1	-5.4	1.7	5.2	Weak	Weak	pCS

Complex	Conformer	Bond	∠(X–H⋯X)	d(Å)	$\rho(r_c)$	$\nabla^2\rho(r_c)$	$V(r_c)$	$H(r_c)$	$E(r_c)$	GC	EC	HC
CB7·1a	1	[O1]7⋯HC5’	144.9	2.434	9.3	35.0	-5.3	1.7	5.2	Weak	Weak	pCS
CB7·1a	1	[O2]1⋯HeC5	104.6	2.664	7.9	31.3	-4.7	1.6	4.8	Weak	Weak	pCS
CB7·1a	1	[O2]2⋯HeC3	104.4	2.665	7.9	31.4	-4.7	1.6	4.8	Weak	Weak	pCS
CB7·1a	1	[O2]3⋯HeC3	154.7	2.344	11.3	42.9	-6.6	2.0	6.2	Weak	Weak	pCS
CB7·1a	1	[O2]4⋯HaC2	115.8	2.768	5.8	21.1	-3.2	1.1	3.7	Weak	Weak	pCS
CB7·1a	1	[O2]5⋯HaC2	144.5	2.871	4.2	14.1	-2.2	0.7	2.9	Weak	Weak	pCS
CB7·1a	1	[O2]5⋯HaC6	144.4	2.873	4.1	14.1	-2.1	0.7	2.9	Weak	Weak	pCS
CB7·1a	1	[O2]6⋯HeC6	115.8	2.768	5.8	21.1	-3.2	1.1	3.7	Weak	Weak	pCS
CB7·1a	1	[O2]7⋯HeC5	154.6	2.344	11.3	42.8	-6.6	2.0	6.2	Weak	Weak	pCS
CB7·1a	1	C2’H⋯HC2	-	1.913	16.3	58.9	-10.4	2.2	8.9	-	Weak	pCS
CB7·1a	1	C6’H⋯HeC6	-	1.913	16.2	58.9	-10.4	2.2	8.9	-	Weak	pCS
CB7·1a	2	[N4]5⋯HC6’	114.7	3.885	0.9	3.1	-0.3	0.2	1.6	Weak	Weak	pCS
CB7·1a	2	[N6]2⋯HC2’	124.5	3.627	1.2	4.5	-0.5	0.3	1.7	Weak	Weak	pCS
CB7·1a	2	[N6]3⋯HC2’	126.5	3.544	1.4	5.4	-0.6	0.4	1.8	Weak	Weak	pCS
CB7·1a	2	[N8]1⋯HaC2	161.6	3.168	2.6	8.9	-1.2	0.5	2.2	Weak	Weak	pCS
CB7·1a	2	[N8]6⋯HC6’	127.8	3.538	1.4	5.5	-0.6	0.4	1.8	Weak	Weak	pCS
CB7·1a	2	[O1]2⋯HC3’	118.0	2.790	5.4	18.9	-2.9	0.9	3.4	Weak	Weak	pCS
CB7·1a	2	[O1]3⋯HC3’	152.3	2.464	8.7	31.9	-4.9	1.5	4.9	Weak	Weak	pCS
CB7·1a	2	[O1]5⋯HC5’	150.4	2.537	7.5	27.0	-4.2	1.3	4.4	Weak	Weak	pCS
CB7·1a	2	[O1]6⋯HC5’	118.4	2.739	5.9	21.0	-3.2	1.0	3.7	Weak	Weak	pCS
CB7·1a	2	[O2]1⋯HaC6	123.4	2.929	4.2	15.2	-2.2	0.8	2.9	Weak	Weak	pCS
CB7·1a	2	[O2]2⋯HaC2	109.2	2.892	4.9	18.8	-2.7	1.0	3.3	Weak	Weak	pCS
CB7·1a	2	[O2]2⋯HeC3	135.5	2.446	9.0	35.3	-5.2	1.8	5.1	Weak	Weak	pCS
CB7·1a	2	[O2]3⋯HeC2	127.7	2.787	5.0	17.9	-2.6	0.9	3.3	Weak	Weak	pCS
CB7·1a	2	[O2]3⋯HeC3	110.5	2.899	4.0	15.9	-2.2	0.9	3.0	Weak	Weak	pCS
CB7·1a	2	[O2]4⋯HaC3	136.2	2.598	6.5	24.1	-3.6	1.2	4.0	Weak	Weak	pCS
CB7·1a	2	[O2]5⋯HaC5	141.6	2.542	7.1	26.9	-4.0	1.4	4.3	Weak	Weak	pCS
CB7·1a	2	[O2]6⋯HeC5	106.3	2.818	5.0	20.2	-2.9	1.1	3.4	Weak	Weak	pCS
CB7·1a	2	[O2]6⋯HeC6	126.8	2.707	6.0	21.6	-3.2	1.1	3.7	Weak	Weak	pCS
CB7·1a	2	[O2]7⋯HaC6	108.4	2.958	4.3	16.8	-2.3	0.9	3.1	Weak	Weak	pCS
CB7·1a	2	[O2]7⋯HeC5	137.8	2.455	8.7	34.0	-5.0	1.7	5.0	Weak	Weak	pCS
CB7·1a	2	C2’H⋯HC2	-	1.946	16.5	62.5	-11.2	2.2	9.5	-	Weak	pCS
CB7·1a	2	C6’H⋯HeC6	-	1.933	16.6	62.3	-11.2	2.2	9.4	-	Weak	pCS
CB7·1b	-	[C7]6⋯HeC6	138.2	3.114	2.3	9.9	-1.1	0.7	2.2	Weak	Weak	pCS

Complex	Conformer	Bond	∠(X–H···X)	d(Å)	$\rho(r_c)$	$\nabla^2\rho(r_c)$	$V(r_c)$	$H(r_c)$	$E(r_c)$	GC	EC	HC
CB7·1b	-	[N2]2···HaC2	118.2	3.591	1.4	4.8	-0.5	0.3	1.8	Weak	Weak	pCS
CB7·1b	-	[N4]2···HaC2	157.7	3.550	1.3	4.4	-0.5	0.3	1.8	Weak	Weak	pCS
CB7·1b	-	[N4]6···HeC6	162.9	3.622	1.1	3.8	-0.4	0.3	1.7	Weak	Weak	pCS
CB7·1b	-	[N6]2···HeC2	142.7	2.950	4.5	14.8	-2.1	0.8	2.9	Weak	Weak	pCS
CB7·1b	-	[N6]7···HaC6	169.8	3.086	3.1	10.7	-1.4	0.6	2.4	Weak	Weak	pCS
CB7·1b	-	[N8]7···HaC6	136.0	3.144	2.9	10.2	-1.3	0.6	2.4	Weak	Weak	pCS
CB7·1b	-	[O1]3···HC3’	124.2	2.552	7.7	29.5	-4.4	1.5	4.5	Weak	Weak	pCS
CB7·1b	-	[O1]4···HC3’	126.5	2.766	5.0	17.8	-2.7	0.9	3.3	Weak	Weak	pCS
CB7·1b	-	[O1]7···HC5’	138.9	2.501	7.9	30.0	-4.4	1.5	4.6	Weak	Weak	pCS
CB7·1b	-	[O1]7···HC6’	113.0	3.125	2.9	12.2	-1.5	0.8	2.5	Weak	Weak	pCS
CB7·1b	-	[O2]2···HeC3	143.7	2.390	10.3	39.2	-6.0	1.9	5.7	Weak	Weak	pCS
CB7·1b	-	[O2]3···HeC3	115.1	2.611	7.3	27.9	-4.2	1.4	4.4	Weak	Weak	pCS
CB7·1b	-	[O2]4···HaC3	160.2	2.434	8.9	33.4	-5.1	1.6	5.0	Weak	Weak	pCS
CB7·1b	-	[O2]5···HaC5	157.7	2.545	7.2	25.8	-3.9	1.3	4.2	Weak	Weak	pCS
CB7·1b	-	[O2]6···HeC5	114.0	2.859	4.5	16.5	-2.4	0.9	3.1	Weak	Weak	pCS
CB7·1b	-	[O2]7···HeC5	144.9	2.316	11.7	46.5	-7.0	2.3	6.5	Weak	Weak	pCS
CB7·1h	1	[N6]7···HaC6	148.1	3.551	1.4	4.5	-0.5	0.3	1.8	Weak	Weak	pCS
CB7·1h	1	[N8]2···HeC2	127.9	3.523	1.5	6.0	-0.6	0.4	1.8	Weak	Weak	pCS
CB7·1h	1	[N8]6···HeC6	154.8	3.605	1.1	3.9	-0.4	0.3	1.7	Weak	Weak	pCS
CB7·1h	1	[O1]3···HC3’	133.6	2.852	3.8	13.8	-2.0	0.7	2.8	Weak	Weak	pCS
CB7·1h	1	[O1]6···HC5’	119.1	2.565	7.8	30.1	-4.5	1.5	4.6	Weak	Weak	pCS
CB7·1h	1	[O1]7···HC5’	134.4	2.544	7.5	27.8	-4.2	1.4	4.4	Weak	Weak	pCS
CB7·1h	1	[O2]1···HaC2	126.8	2.800	5.8	21.0	-3.1	1.1	3.6	Weak	Weak	pCS
CB7·1h	1	[O2]2···HeC3	156.6	2.405	9.6	36.2	-5.5	1.8	5.4	Weak	Weak	pCS
CB7·1h	1	[O2]3···HaC3	104.1	2.861	5.1	20.6	-2.9	1.1	3.5	Weak	Weak	pCS
CB7·1h	1	[O2]4···HaC3	163.8	2.561	7.1	24.9	-3.8	1.2	4.2	Weak	Weak	pCS
CB7·1h	1	[O2]4···HaC5	145.3	3.043	2.8	9.6	-1.4	0.5	2.4	Weak	Weak	pCS
CB7·1h	1	[O2]5···HaC5	126.8	2.700	5.8	20.5	-3.1	1.0	3.6	Weak	Weak	pCS
CB7·1h	1	[O2]6···HeC5	133.0	2.509	8.0	30.3	-4.5	1.5	4.6	Weak	Weak	pCS
CB7·1h	1	[O2]7···HaC6	120.2	2.741	6.3	22.9	-3.4	1.2	3.8	Weak	Weak	pCS
CB7·1h	1	[O2]7···HeC5	119.6	2.566	7.8	30.1	-4.5	1.5	4.6	Weak	Weak	pCS
CB7·1h	2	[N2]5···HeC6	146.4	3.551	1.3	4.5	-0.5	0.3	1.8	Weak	Weak	pCS
CB7·1h	2	[N4]2···HaC2	120.6	3.633	1.3	4.4	-0.5	0.3	1.7	Weak	Weak	pCS
CB7·1h	2	[N4]6···HeC6	155.2	3.551	1.3	4.6	-0.5	0.3	1.8	Weak	Weak	pCS

Complex	Conformer	Bond	∠(X–H···X)	d(Å)	ρ(r_c)	$\nabla^2\rho(r_c)$	V(r_c)	H(r_c)	E(r_c)	GC	EC	HC
CB7·1h	2	[N6]2···HaC2	118.5	3.550	1.4	5.3	-0.6	0.4	1.8	Weak	Weak	pCS
CB7·1h	2	[N8]2···HeC2	122.3	3.539	1.4	5.3	-0.6	0.4	1.8	Weak	Weak	pCS
CB7·1h	2	[O1]3···HC3’	134.1	2.680	5.4	20.0	-3.0	1.0	3.5	Weak	Weak	pCS
CB7·1h	2	[O1]4···HC3’	113.3	2.797	5.3	19.4	-2.8	1.0	3.4	Weak	Weak	pCS
CB7·1h	2	[O1]6···HC5’	115.3	2.603	7.5	28.9	-4.3	1.5	4.5	Weak	Weak	pCS
CB7·1h	2	[O1]7···HC5’	134.8	2.696	5.4	19.4	-2.9	1.0	3.5	Weak	Weak	pCS
CB7·1h	2	[O2]1···HaC2	122.2	2.954	4.3	15.8	-2.2	0.9	3.0	Weak	Weak	pCS
CB7·1h	2	[O2]2···HeC3	159.7	2.393	9.9	37.1	-5.7	1.8	5.5	Weak	Weak	pCS
CB7·1h	2	[O2]3···HaC3	104.4	2.939	4.3	17.2	-2.3	1.0	3.1	Weak	Weak	pCS
CB7·1h	2	[O2]4···HaC3	166.6	2.370	10.1	38.5	-5.9	1.9	5.6	Weak	Weak	pCS
CB7·1h	2	[O2]5···HaC5	127.9	2.517	8.4	31.2	-4.7	1.5	4.8	Weak	Weak	pCS
CB7·1h	2	[O2]6···HeC5	128.9	2.585	7.0	26.0	-3.9	1.3	4.2	Weak	Weak	pCS
CB7·1h	2	[O2]6···HeC6	112.3	3.129	3.1	12.9	-1.6	0.8	2.5	Weak	Weak	pCS
CB7·1h	2	[O2]7···HaC6	115.5	2.876	4.8	17.9	-2.6	0.9	3.2	Weak	Weak	pCS
CB7·1h	2	[O2]7···HeC5	126.4	2.495	8.5	33.2	-4.9	1.7	4.9	Weak	Weak	pCS
CB7·1i	1	[N4]2···HaC2	122.8	3.592	1.4	4.6	-0.5	0.3	1.8	Weak	Weak	pCS
CB7·1i	1	[N4]6···HeC6	155.6	3.550	1.3	4.6	-0.5	0.3	1.8	Weak	Weak	pCS
CB7·1i	1	[N6]2···HaC2	120.6	3.552	1.4	5.3	-0.6	0.4	1.8	Weak	Weak	pCS
CB7·1i	1	[N8]2···HeC2	120.4	3.549	1.4	5.2	-0.6	0.4	1.8	Weak	Weak	pCS
CB7·1i	1	[O1]3···HC3’	135.5	2.667	5.5	20.4	-3.0	1.0	3.6	Weak	Weak	pCS
CB7·1i	1	[O1]4···HC3’	113.7	2.776	5.5	20.1	-2.9	1.0	3.5	Weak	Weak	pCS
CB7·1i	1	[O1]6···HC5’	115.8	2.587	7.7	29.8	-4.4	1.5	4.6	Weak	Weak	pCS
CB7·1i	1	[O1]7···HC5’	135.5	2.720	5.1	18.4	-2.8	0.9	3.4	Weak	Weak	pCS
CB7·1i	1	[O2]1···HaC2	122.0	3.012	3.8	14.2	-1.9	0.8	2.8	Weak	Weak	pCS
CB7·1i	1	[O2]2···HeC3	160.4	2.405	9.7	36.0	-5.5	1.8	5.4	Weak	Weak	pCS
CB7·1i	1	[O2]4···HaC3	166.9	2.378	10.0	37.8	-5.8	1.8	5.6	Weak	Weak	pCS
CB7·1i	1	[O2]5···HaC5	130.9	2.513	8.3	30.8	-4.7	1.5	4.7	Weak	Weak	pCS
CB7·1i	1	[O2]6···HeC5	127.5	2.579	7.2	26.7	-4.0	1.3	4.3	Weak	Weak	pCS
CB7·1i	1	[O2]6···HeC6	112.7	3.117	3.2	13.1	-1.6	0.8	2.6	Weak	Weak	pCS
CB7·1i	1	[O2]7···HaC6	113.9	2.927	4.4	16.8	-2.4	0.9	3.1	Weak	Weak	pCS
CB7·1i	1	[O2]7···HeC5	128.9	2.459	9.1	35.6	-5.3	1.8	5.2	Weak	Weak	pCS
CB7·1i	2	[C7]3···HeC2	130.5	3.136	2.2	9.5	-1.1	0.7	2.1	Weak	Weak	pCS
CB7·1i	2	[N2]6···HeC6	150.9	3.548	1.3	4.6	-0.5	0.3	1.8	Weak	Weak	pCS
CB7·1i	2	[N4]3···HeC2	163.3	3.548	1.2	4.5	-0.5	0.3	1.7	Weak	Weak	pCS

Complex	Conformer	Bond	∠(X–H···X)	d(Å)	ρ(r_c)	∇^2ρ(r_c)	V(r_c)	H(r_c)	E(r_c)	GC	EC	HC
CB7·1i	2	[N8]7···HaC6	152.3	2.750	6.7	22.3	-3.3	1.2	3.7	Weak	Weak	pCS
CB7·1i	2	[O1]1···HC5’	108.1	2.898	4.7	17.3	-2.4	0.9	3.1	Weak	Weak	pCS
CB7·1i	2	[O1]2···HC5’	132.6	2.647	5.9	21.9	-3.3	1.1	3.7	Weak	Weak	pCS
CB7·1i	2	[O1]2···HC6’	116.7	3.053	3.1	12.2	-1.6	0.7	2.5	Weak	Weak	pCS
CB7·1i	2	[O1]5···HC3’	130.5	2.733	5.0	18.3	-2.7	0.9	3.3	Weak	Weak	pCS
CB7·1i	2	[O1]6···HC3’	122.6	2.579	7.3	27.8	-4.1	1.4	4.3	Weak	Weak	pCS
CB7·1i	2	[O2]1···HaC2	127.3	2.855	5.0	17.9	-2.6	0.9	3.2	Weak	Weak	pCS
CB7·1i	2	[O2]2···HeC3	152.5	2.259	13.1	51.8	-8.1	2.4	7.2	Weak	Weak	pCS
CB7·1i	2	[O2]3···HeC3	106.9	2.783	5.7	22.7	-3.2	1.2	3.7	Weak	Weak	pCS
CB7·1i	2	[O2]4···HaC3	174.1	2.884	3.6	12.1	-1.8	0.6	2.7	Weak	Weak	pCS
CB7·1i	2	[O2]5···HaC5	155.6	2.764	4.6	15.6	-2.4	0.8	3.1	Weak	Weak	pCS
CB7·1i	2	[O2]6···HeC5	123.6	2.470	9.2	35.7	-5.3	1.8	5.2	Weak	Weak	pCS
CB7·1i	2	[O2]7···HeC5	135.9	2.423	9.9	37.6	-5.7	1.8	5.5	Weak	Weak	pCS
CB7·2a	1	[N4]4···HeC2	149.6	3.500	1.4	5.2	-0.6	0.4	1.8	Weak	Weak	pCS
CB7·2a	1	[N6]7···HeC6	120.9	3.551	1.4	5.6	-0.6	0.4	1.8	Weak	Weak	pCS
CB7·2a	1	[O1]2···HC3’	110.3	3.004	3.6	13.2	-1.8	0.7	2.7	Weak	Weak	pCS
CB7·2a	1	[O1]3···HC3’	146.3	2.402	9.6	37.0	-5.6	1.8	5.4	Weak	Weak	pCS
CB7·2a	1	[O1]6···HC5’	142.4	2.483	8.3	30.9	-4.7	1.5	4.7	Weak	Weak	pCS
CB7·2a	1	[O1]7···HC5’	116.2	2.675	6.4	24.1	-3.6	1.2	4.0	Weak	Weak	pCS
CB7·2a	1	[O2]1···HaC6	124.1	2.906	4.6	16.9	-2.4	0.9	3.1	Weak	Weak	pCS
CB7·2a	1	[O2]2···HaC2	115.6	2.897	4.9	18.7	-2.7	1.0	3.3	Weak	Weak	pCS
CB7·2a	1	[O2]2···HeC3	125.8	2.471	9.1	35.5	-5.3	1.8	5.2	Weak	Weak	pCS
CB7·2a	1	[O2]3···HeC3	130.2	2.538	7.7	28.9	-4.3	1.4	4.5	Weak	Weak	pCS
CB7·2a	1	[O2]4···HaC3	130.0	2.694	5.6	20.1	-3.1	1.0	3.6	Weak	Weak	pCS
CB7·2a	1	[O2]5···HaC3	145.7	3.106	2.5	8.6	-1.2	0.5	2.2	Weak	Weak	pCS
CB7·2a	1	[O2]5···HaC5	161.6	2.630	6.2	21.3	-3.3	1.0	3.8	Weak	Weak	pCS
CB7·2a	1	[O2]7···HeC5	153.2	2.400	9.7	36.7	-5.6	1.8	5.4	Weak	Weak	pCS
CB7·2a	2	[N6]2···HaC2	117.1	3.550	1.4	5.1	-0.6	0.3	1.8	Weak	Weak	pCS
CB7·2a	2	[O1]3···HC3’	134.8	2.660	5.8	21.0	-3.2	1.0	3.7	Weak	Weak	pCS
CB7·2a	2	[O1]4···HC3’	119.9	2.631	6.8	25.4	-3.8	1.3	4.1	Weak	Weak	pCS
CB7·2a	2	[O1]6···HC5’	111.6	2.674	6.8	25.7	-3.8	1.3	4.1	Weak	Weak	pCS
CB7·2a	2	[O1]7···HC5’	142.4	2.549	7.1	26.3	-4.0	1.3	4.2	Weak	Weak	pCS
CB7·2a	2	[O2]1···HaC2	120.7	2.985	4.2	16.0	-2.2	0.9	3.0	Weak	Weak	pCS
CB7·2a	2	[O2]2···HeC3	158.0	2.372	10.2	38.9	-5.9	1.9	5.7	Weak	Weak	pCS

Complex	Conformer	Bond	∠(X–H···X)	d(Å)	ρ(r_c)	$\nabla^2\rho(r_c)$	V(r_c)	H(r_c)	E(r_c)	GC	EC	HC
CB7·2a	2	[O2]3···HaC3	104.6	2.756	6.3	25.3	-3.6	1.3	4.0	Weak	Weak	pCS
CB7·2a	2	[O2]4···HaC3	161.1	2.557	7.3	25.5	-4.0	1.2	4.2	Weak	Weak	pCS
CB7·2a	2	[O2]4···HaC5	148.1	2.889	3.8	12.8	-1.9	0.6	2.8	Weak	Weak	pCS
CB7·2a	2	[O2]5···HaC5	121.4	2.625	6.9	25.4	-3.8	1.2	4.2	Weak	Weak	pCS
CB7·2a	2	[O2]6···HeC5	142.0	2.529	7.4	27.7	-4.1	1.4	4.4	Weak	Weak	pCS
CB7·2a	2	[O2]7···HaC6	118.7	3.028	3.9	15.4	-2.1	0.9	2.9	Weak	Weak	pCS
CB7·2a	2	[O2]7···HeC5	123.6	2.670	6.2	22.7	-3.4	1.1	3.9	Weak	Weak	pCS

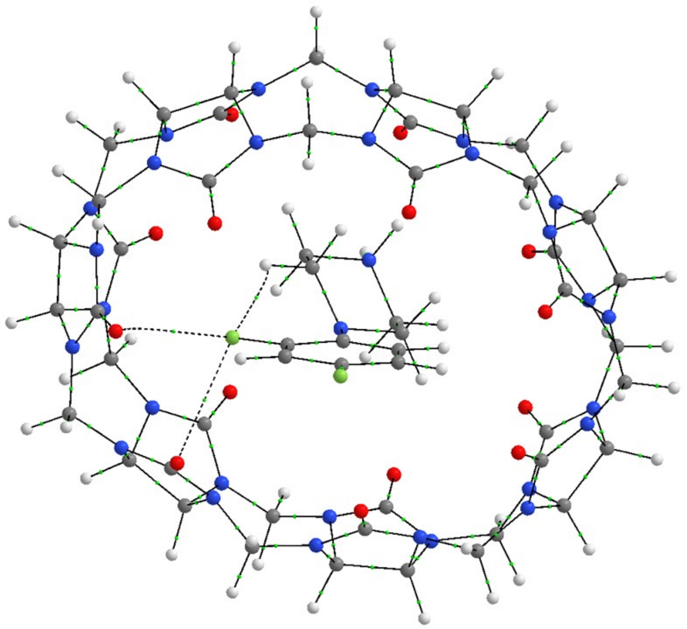

Fig. S18 Molecular graph for CB7·**1h** conformer 1. Dashed lines indicate a fluorine interaction. The small green dots indicate the bond critical points. White atoms are hydrogen, gray atoms are carbon, red atoms are oxygen, blue atoms are nitrogen, and green atoms are fluorine.

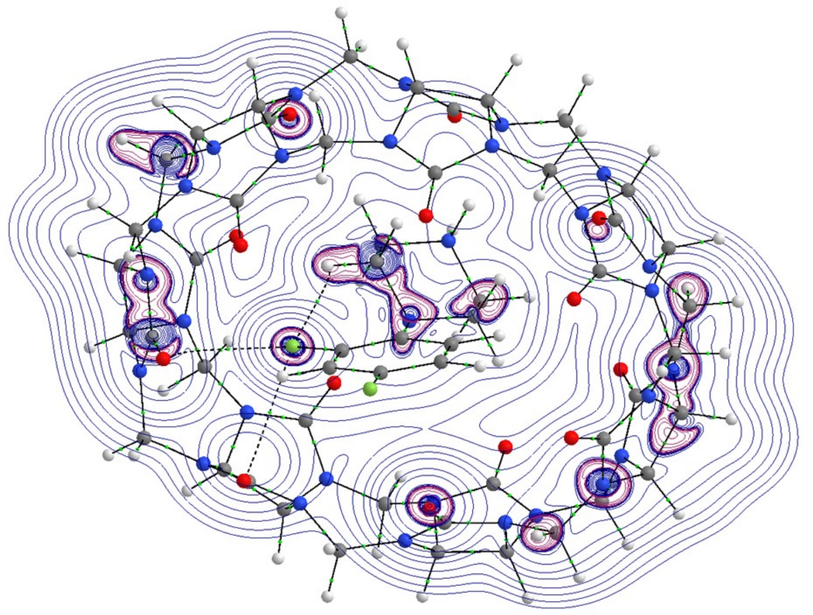

Fig. S19 Contour plot of the Laplacian of the electron density for CB7·**1h** conformer 1. The selected plane is formed between those atoms that belong to the intramolecular bond C2H···FC2'. Regions of charge-concentration and charge-depletion are respectively solid lines in red, $\nabla^2\rho(r_c) < 0$, and solid lines in blue, $\nabla^2\rho(r_c) > 0$. Bond paths between each atom pair of two nuclei painted in solid and dashed lines in atom color represent the shared- and closed-shell interactions, respectively, whereas the tiny spheres painted in green represent bond critical points.

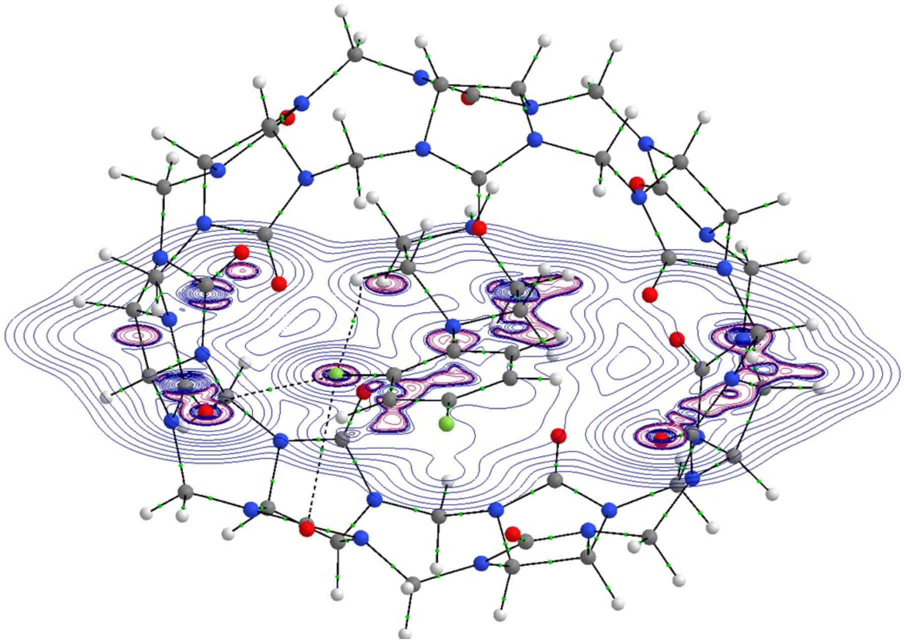

Fig. S20 Contour plot of the Laplacian of the electron density for CB7·**1h** conformer 1. The selected plane is formed between those atoms that belong to the intramolecular bond [O1]3···FC2'. Regions of

charge-concentration and charge-depletion are respectively solid lines in red, $\nabla^2\rho(r_c) < 0$, and solid lines in blue, $\nabla^2\rho(r_c) > 0$. Bond paths between each atom pair of two nuclei painted in solid and dashed lines in atom color represent the shared- and closed-shell interactions, respectively, whereas the tiny spheres painted in green represent bond critical points.

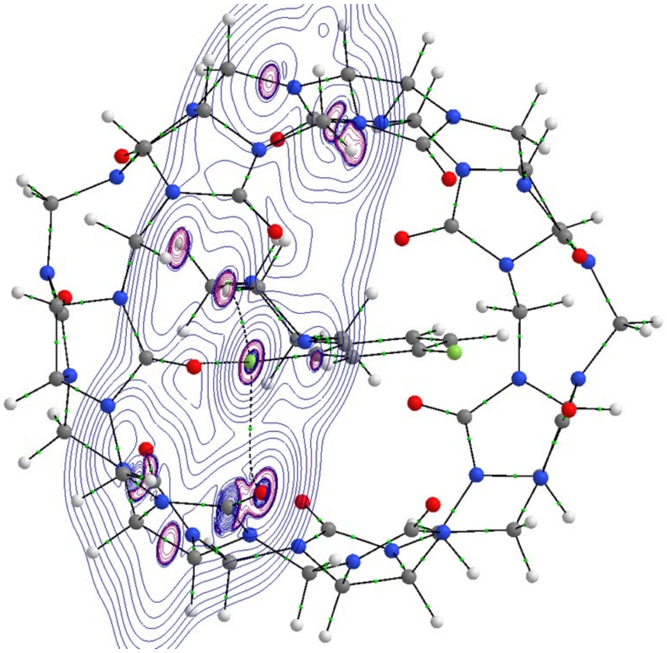

Fig. S21 Contour plot of the Laplacian of the electron density for CB7·**1h** conformer 1. The selected plane is formed between those atoms that belong to the intramolecular bond [O1]4···FC2'. Regions of charge-concentration and charge-depletion are respectively solid lines in red, $\nabla^2\rho(r_c) < 0$, and solid lines in blue, $\nabla^2\rho(r_c) > 0$. Bond paths between each atom pair of two nuclei painted in solid and dashed lines in atom color represent the shared- and closed-shell interactions, respectively, whereas the tiny spheres painted in green represent bond critical points.

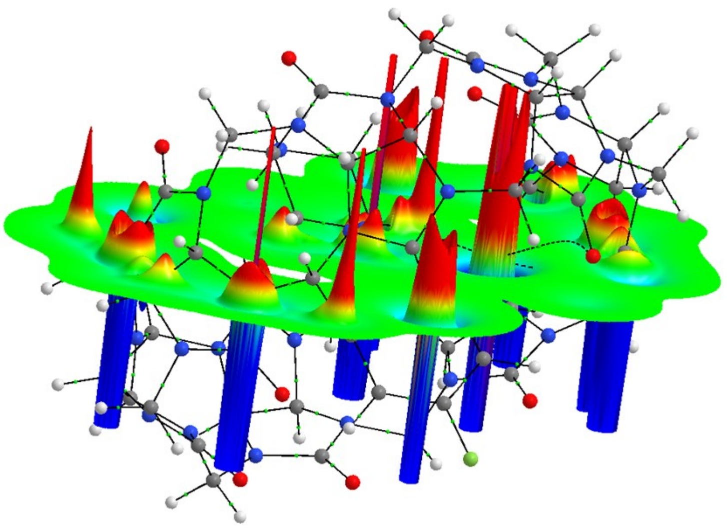

Fig. S22 Relief maps of the Laplacian of the density for conformer 1 of CB7·**1h** conformer 1. The selected plane is formed between those atoms that belong to the intramolecular bond C2H···FC2’. The color ranges are: blue, more positive than 1; cyan, between 1 and 0.5; green, between −0.5 and 0.5; yellow, between −1 and −0.5; red, more negative than −1 au.

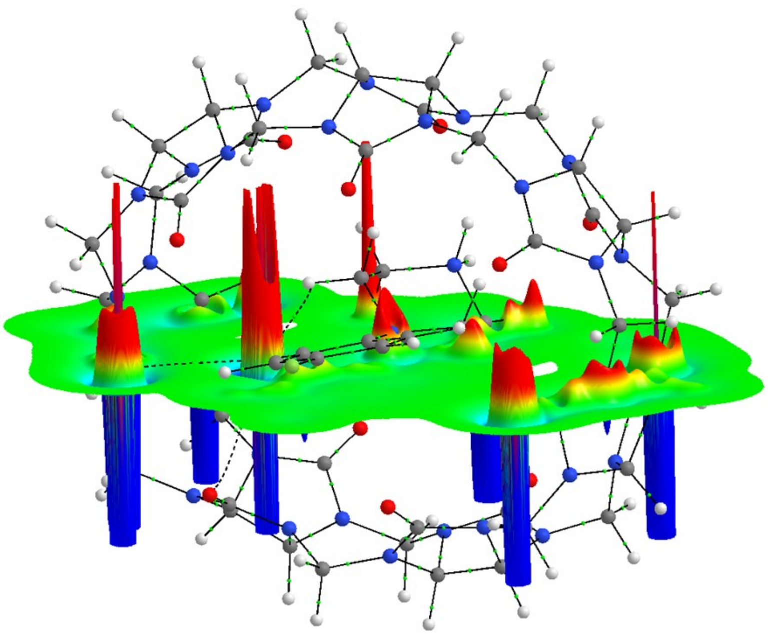

Fig. S23 Relief maps of the Laplacian of the density for conformer 1 of CB7·**1h** conformer 1. The selected plane is formed between those atoms that belong to the intramolecular bond [O1]3···FC2'. The color ranges are: blue, more positive than 1; cyan, between 1 and 0.5; green, between −0.5 and 0.5; yellow, between −1 and −0.5; red, more negative than −1 au.

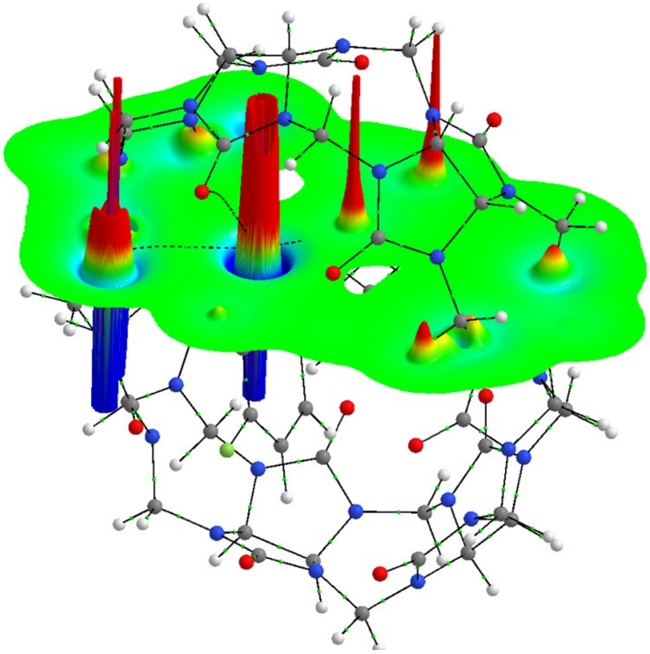

Fig. S24 Relief maps of the Laplacian of the density for conformer 1 of CB7·**1h** conformer 1. The selected plane is formed between those atoms that belong to the intramolecular bond [O1]4···FC2'. The color ranges are: blue, more positive than 1; cyan, between 1 and 0.5; green, between −0.5 and 0.5; yellow, between −1 and −0.5; red, more negative than −1 au.

Table S10 Geometrical parameters, QTAIM properties at the bond critical points (in a.u. x 10^3), $E(r_c)$ is in kJ/mol, and classification of n → π* interactions. EC corresponds to energy classification and HC stands for Hayashi classification. The number in square brackets corresponds to the glycolurill unit in CB7·

Complex	Conformer	Bond	d(Å)	$\rho(r_c)$	$\nabla^2\rho(r_c)$	$V(r_c)$	$H(r_c)$	$E(r_c)$	EC	HC
CB7·1a	1	[O1]1···C5'	3.471	3.8	12.3	-1.7	0.7	2.6	Weak	pCS
CB7·1a	1	[O1]2···C3'	3.472	3.8	12.3	-1.7	0.7	2.6	Weak	pCS
CB7·1a	1	[O1]5···C1'	3.706	2.9	9.9	-1.3	0.6	2.4	Weak	pCS
CB7·1a	2	[N8]1···C6'	4.621	0.7	2.1	-0.2	0.1	1.6	Weak	pCS
CB7·1a	2	[O1]1···C4'	3.796	2.3	6.6	-0.9	0.4	2.1	Weak	pCS
CB7·1a	2	[O1]4···C3'	3.324	4.9	16.9	-2.4	0.9	3.1	Weak	pCS
CB7·1a	2	[O1]5···C5'	3.345	4.8	16.3	-2.3	0.9	3.0	Weak	pCS
CB7·1b	-	[O1]1···C5'	3.440	4.0	14.6	-2.0	0.8	2.8	Weak	pCS
CB7·1b	-	[O1]2···C3'	3.554	3.5	11.8	-1.6	0.7	2.6	Weak	pCS

CB7·1b	-	[O1]5···C3’	3.422	4.2	13.3	-1.9	0.7	2.8	Weak	pCS
CB7·1b	-	[O1]6···C5’	3.622	1.1	3.8	-0.4	0.3	1.7	Weak	pCS
CB7·1h	1	[O1]1···C5’	3.398	4.4	14.2	-2.1	0.7	2.9	Weak	pCS
CB7·1h	1	[O1]2···C3’	3.294	5.1	18.6	-2.6	1.0	3.3	Weak	pCS
CB7·1h	1	[O1]4···C3’	3.386	4.4	16.5	-2.3	0.9	3.0	Weak	pCS
CB7·1h	1	[O1]5···C5’	3.404	4.6	15.4	-2.2	0.8	3.0	Weak	pCS
CB7·1h	2	[O1]1···C5’	3.466	3.7	12.0	-1.7	0.7	2.6	Weak	pCS
CB7·1h	2	[O1]2···C3’	3.341	4.5	15.3	-2.2	0.8	3.0	Weak	pCS
CB7·1h	2	[O1]5···C4’	3.380	4.6	17.0	-2.3	1.0	3.0	Weak	pCS
CB7·1i	1	[O1]1···C5’	3.455	3.7	12.2	-1.7	0.7	2.6	Weak	pCS
CB7·1i	1	[O1]2···C3’	3.322	4.6	15.8	-2.3	0.9	3.0	Weak	pCS
CB7·1i	1	[O1]5···C4’	3.373	4.7	17.4	-2.4	1.0	3.1	Weak	pCS
CB7·1i	2	[O1]1···C3’	3.396	4.9	17.5	-2.4	1.0	3.1	Weak	pCS
CB7·1i	2	[O1]3···C5’	3.291	5.2	17.7	-2.5	1.0	3.2	Weak	pCS
CB7·1i	2	[O1]4···C3’	3.401	4.2	14.0	-2.0	0.8	2.8	Weak	pCS
CB7·2a	1	[O1]1···C4’	3.848	2.0	6.2	-0.8	0.4	2.0	Weak	pCS
CB7·2a	1	[O1]4···C3’	3.172	5.8	21.7	-3.1	1.2	3.6	Weak	pCS
CB7·2a	1	[O1]5···C5’	3.251	4.9	18.1	-2.5	1.0	3.2	Weak	pCS
CB7·2a	2	[O1]1···C3’	3.457	3.3	11.6	-1.6	0.7	2.5	Weak	pCS
CB7·2a	2	[O1]4···C4’	3.438	4.6	15.3	-2.2	0.8	2.9	Weak	pCS
CB7·2a	2	[O1]7···C5’	3.387	3.7	13.7	-1.8	0.8	2.7	Weak	pCS

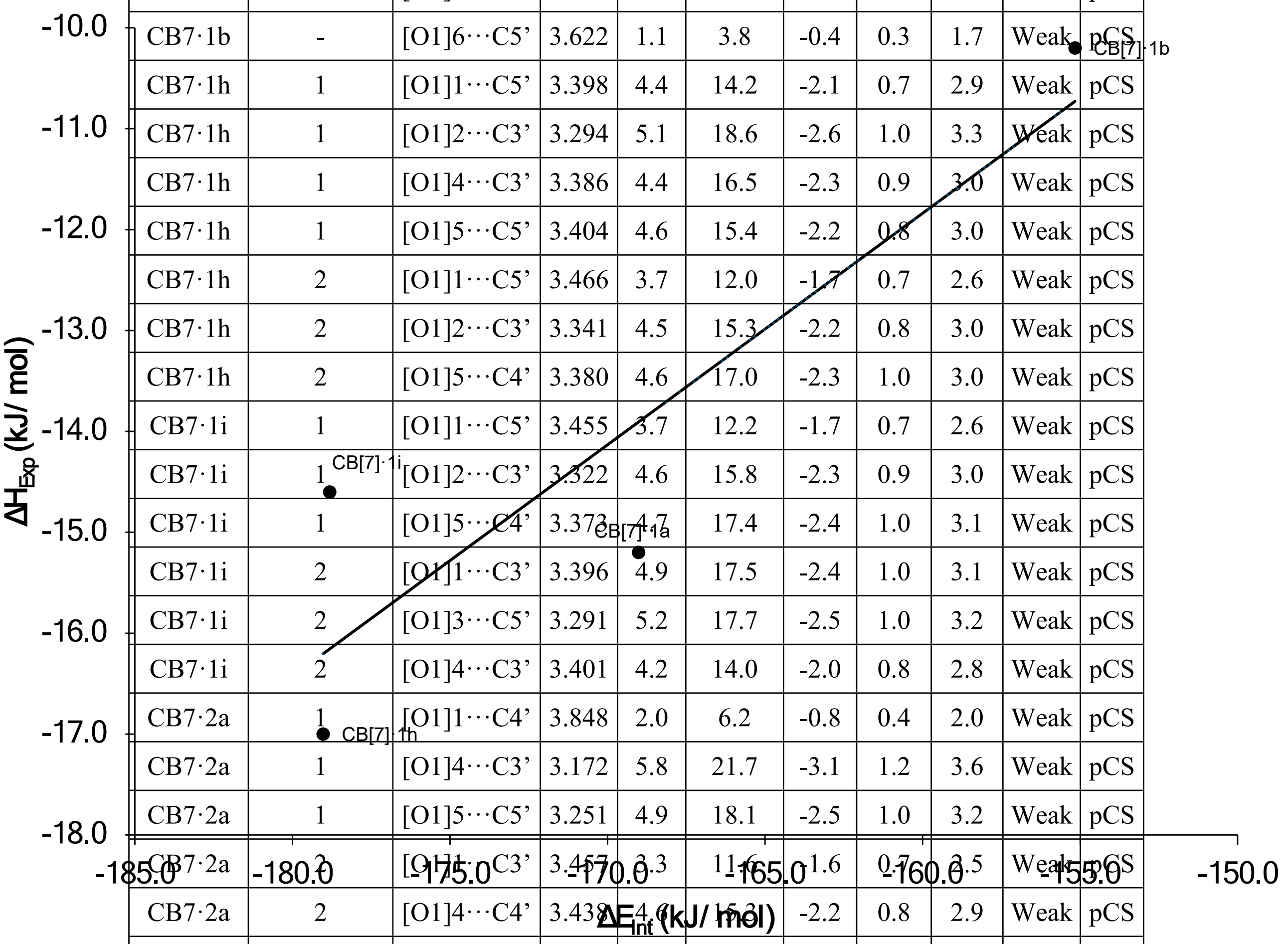

Fig. S25

Experimental enthalpy energy (Table 2) versus interaction energy. The equation for the adjusted tendency (Continuous line) is $y = 0.2295\ x + 24.8810$ and $R^2=0.80$.

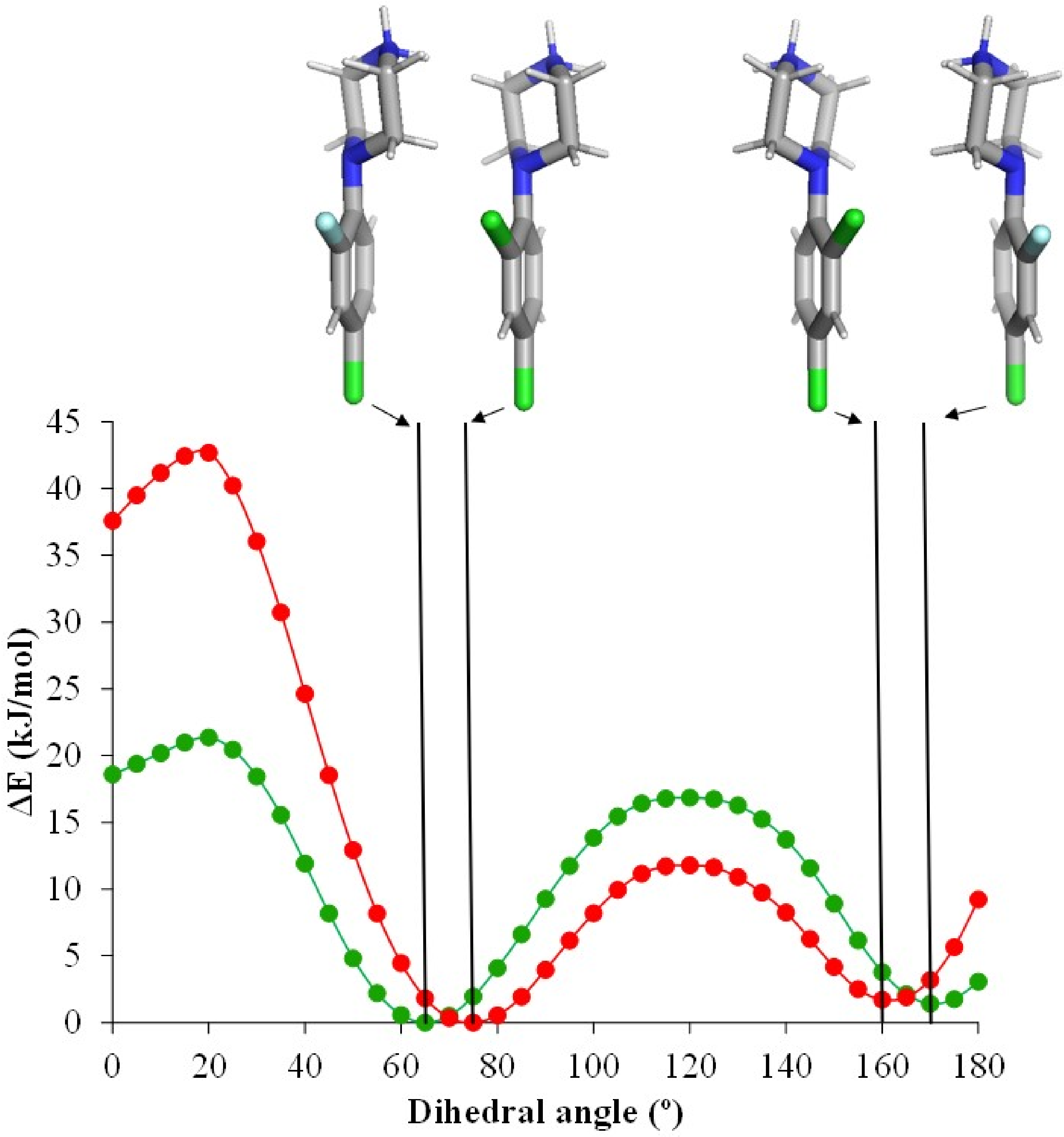

Fig. S26 Potential energy surfaces for the dihedral angle C2'-C1'-N1-C1 are shown for 1i in (●) and 1j in (●) with the structures at the minima overlaid.
